# Cavitation Fibrillation of Cellulose Fiber

**DOI:** 10.1021/acs.biomac.1c01309

**Published:** 2022-01-31

**Authors:** Jakob D. Redlinger-Pohn, Martin Petkovšek, Korneliya Gordeyeva, Mojca Zupanc, Alisa Gordeeva, Qilun Zhang, Matevž Dular, L. Daniel Söderberg

**Affiliations:** † Department of Fibre and Polymer Technology, KTH Royal Institute of Technology, Teknikringen 56−58, 114 28 Stockholm, Sweden; ‡ Treesearch, Teknikringen 38a, 114 28 Stockholm, Sweden; § Laboratory for Water and Turbine Machines, Faculty of Mechanical Engineering, University of Ljubljana, Aškerčeva 6, 1000 Ljubljana, Slovenia; ∥ Department of Materials and Environmental Chemistry, Stockholm University, Svante Arrhenius väg 16 C, 114 18 Stockholm, Sweden; ⊥ Laboratory of Organic Electronics, Linköping University, Campus Calla, Olaus Magnus väg 37, 583 30 Linköping, Sweden

## Abstract

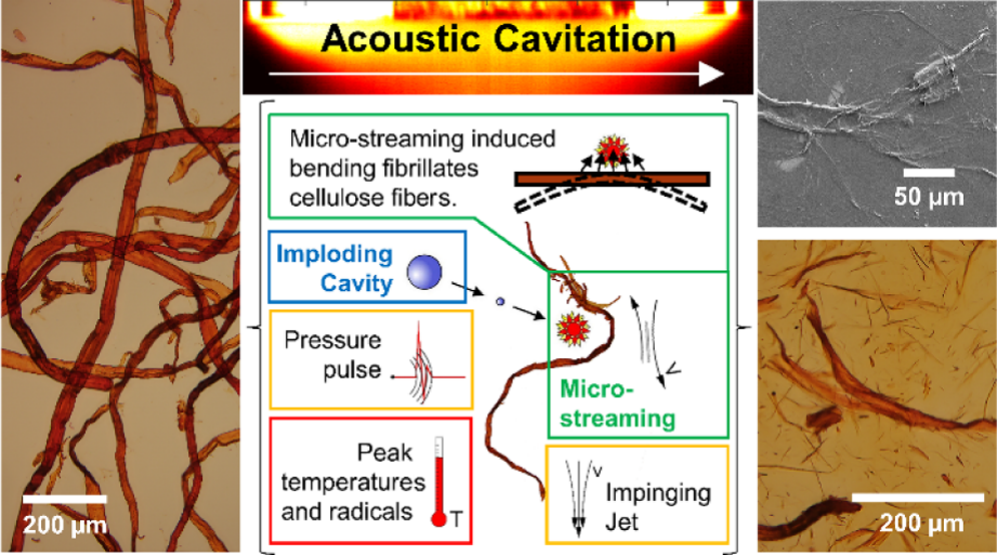

Cellulose fibrils are the structural backbone of plants and, if carefully liberated from biomass, a promising building block for a bio-based society. The mechanism of the mechanical release—fibrillation—is not yet understood, which hinders efficient production with the required reliable quality. One promising process for fine fibrillation and total fibrillation of cellulose is cavitation. In this study, we investigate the cavitation treatment of dissolving, enzymatically pretreated, and derivatized (TEMPO oxidized and carboxymethylated) cellulose fiber pulp by hydrodynamic and acoustic (i.e., sonication) cavitation. The derivatized fibers exhibited significant damage from the cavitation treatment, and sonication efficiently fibrillated the fibers into nanocellulose with an elementary fibril thickness. The breakage of cellulose fibers and fibrils depends on the number of cavitation treatment events. In assessing the damage to the fiber, we presume that microstreaming in the vicinity of imploding cavities breaks the fiber into fibrils, most likely by bending. A simple model showed the correlation between the fibrillation of the carboxymethylated cellulose (CMCe) fibers, the sonication power and time, and the relative size of the active zone below the sonication horn.

## 1 Introduction

Cellulose nanofibrils (CNFs) are receiving increasing interest as the building blocks of high-performance materials from renewable sources. A key characteristic is their slenderness, that is, a high-aspect ratio with thicknesses in the order of nanometers.^1^ Most commonly, CNFs are produced from wood fibers and their derivatives by mechanical treatment,^2−5^ such as homogenization and microfluidization, which are unfortunately limited in reducing the fibril thickness.^6^ Sonication is employed as the post-treatment,^7,8^ or the only treatment^9^ to produce CNFs at the cellulose fibril elementary scale.^10−12^ Unfortunately, sonication not only reduces the fibril thickness but also the fibril length,^9^ which is detrimental to any material design that utilizes CNF aggregation capabilities. Despite the widespread application of sonication processes in cellulose fibrillation, the process itself is little understood and observations are typically described only qualitatively. A mechanistical model and process understanding are missing, which complicates the reproduction and effectively jeopardizes the development of sustainable nanocellulose manufacturing processes at a larger scale. To build a mechanistical sound understanding, first the cavitation action (listed in the next paragraph) leading to cellulose fibrillation needs to be identified as the basis to describe how fibrillation processing parameters impact the final CNF quality. It is therefore that we present in this paper a holistic discussion of the cellulose fiber fibrillation by cavitation from which we deduce key insights to formulate scale-up strategies and enable a mechanistic-based optimization in future work.

Cavitation as a physical phenomenon describes the growth and collapse of small cavities, depictable as vaporous bubbles, within the liquid due to a local pressure drop^13^ from acoustic sound waves (acoustic cavitation, also referred to as sonication) or a local velocity increase (hydrodynamic cavitation). The cavities collapse rapidly once the local static pressure increases above the vapor pressure, giving rise to localized large hydrodynamic forces (in the form of pressure waves of several MPa,^14^ microjets with velocities >100 m/s,^15^ and high shear flow on a microscale, i.e., microstreaming) and peak temperatures of several 1000 K,^16^ causing water dissociation and the formation of hydroxy radicals (^•^OH). The number and size of the vapor cavities in hydrodynamic cavitation depends on the velocity and pressure conditions and can be characterized as (i) attached steady cavitation, (ii) developed unsteady cavitation, also known as cloud shedding, and (iii) supercavitation, where a single cavity fills a large volume of the flow tract.^13,17^ Acoustic cavitation can be roughly divided into ultrasonic baths and ultrasonic horns,^18^ where the sound waves are emitted by oscillating piezoelectric elements at >20 kHz (where ∼20 kHz is often reported for the cellulose treatment). Ultrasonic horns concentrate the cavities in a small region beneath an oscillating tip, hence being a focused treatment at high energy density. The number and size of the formed cavities scales nonlinearly with the oscillation amplitude.

Hydrodynamic and acoustic cavitation are known and employed for the modification of cellulose fiber properties while preserving the fiber shape, that is, internal fibrillation,^19−22^ and, especially acoustic cavitation, for the external fibrillation of cellulose fibers and the production of nanocellulose.^9,23−28^ For that, cohesive forces between the fibrils need to be overcome by external stressing. Higher treatment intensity and exposure time increase the degree of fibrillation and benefit the development of the cellulose fibers to smaller sizes.^24,29^ However, also the fibril length and cellulose crystallinity (i.e., the regions of the structured organization of the glucose polymer and its constituting glucose monomer) are decreasing, which is often regarded as quality degradation.^9,30,31^ Cavitation, especially acoustic cavitation, is also employed to intensify chemical modification.^16^ Surprisingly, the potential threat of cellulose chemical deterioration is often ignored by research that focuses on the mechanical aspects, although it is an important parameter to assess the all over quality of nanocellulose from cavitation treatment.

It is our impression that research on cellulose cavitation treatment focuses largely on application tests while ignoring to describe the process itself, which hinders the results’ interpretation. Exceptions include the work of Saito et al.,^30^ who discuss fibril shortening as axial tension breakage, that is, scission,^32^ and Zhou et al.,^33^ who demonstrate that the formation of kinks^34^ decreases with the processing time and decreasing fibril length. These damage types are not unique to the treatment of cellulose fibrils and have been, for example, discussed previously for carbon nanotubes (CNTs).^32,35,36^ Remarkable is the work of Pagani et al.,^35^ who modeled the reaction of CNTs in the vicinity of an imploding cavity. They documented that short CNTs are indeed suspected to break from scission, while long CNTs, however, break by bending. Bending failure was recently discussed by Redlinger-Pohn et al.^6^ as a leading mechanism in hyper-inertia fibrillation (i.e., microfluidization and homogenization), but it appears to us that it is not considered in cavitation fibrillation.

Another remark on the current state of cellulose sonication research is the aforementioned missing mechanistical discussion. This discussion needs a broad basis (e.g., several types of cellulose and treatment intensity) and needs to consider the changes in the cellulose biomacromolecules, that is, CNFs, in relation to the cavitation action.

It is therefore, that we start the investigation in this paper from a broad basis, subjecting dissolving pulp cellulose fibers, and fibers whose structures have been enzymatically^37,38^ and chemically weakened by derivatization (TEMPO oxidation^39,40^ and carboxymethylation^41,42^) to hydrodynamic and acoustic cavitation. The cases of extensive mechanical treatment with a successful fibrillation to the CNF were then further analyzed for their changes in the cellulose crystallinity and chemical composition. From the extensive analytical investigation, we finally derived a process model and present suggestions for the production of high-aspect ratio CNF by cavitation treatment.

## 2 Materials and Methods

The effect of hydrodynamic cavitation in a blow-through (BT) device and acoustic cavitation with an ultrasound horn (UH) was tested on four different types of pulp.

### 2.1 Cellulose Pulp and Sample Preparation

Dissolving cellulose fiber pulp (DCe, Domsjö, Sweden), enzymatic pretreated cellulose (ECe),^37,38^ and carboxymethylated cellulose (CMCe)^41^ at a total charge of SC_total_ ∼ 600 μeq/g were provided by RISE Bioeconomy (Sweden). TEMPO-mediated oxidized cellulose (TCe) was prepared from DCe following the protocol of Saito et al.^43^ to SC_total_ ∼ 600 μeq/g. ECe was mechanically pretreated by refining so that the initial fiber length was smaller compared to the other fibers. CMCe and TCe are easier to disperse due to the presence of polar negatively charged groups (carboxyl and carboxymethyl), which electrostatically repulse each other and help in fiber swelling, promoting fibrillation at lower energy input.^39−42^

The fiber concentration was guided by the necessity of bubble motion in batch ultrasonication, resulting in a dispersion, not a gel with the connectivity limit as the maximum.^44^ The mass-based concentration *C*_m_ for a fibril with a typical aspect ratio of 100 is calculated to ∼0.5%. Including a safety margin, we decided on *C*_m_ = 0.4%, which is also identical to the concentration used in our previous study of fibrillation by hyper-inertia flows.^6^ A subset of experiments were performed at a higher concentration of *C*_m_ = 2%, what is used in industrial homogenization.^45^ The dispersions of DCe, CMCe, and TCe were prepared from their flocculated pulp at *C*_m_ of 41.6, 22.2, and 15.6%, respectively. The pulps were diluted with deionized water and soaked overnight before being dispersed by mechanical stirring. ECe was already diluted at a *C*_m_ of 2% and further diluted with deionized water under mechanical stirring to 0.4%. At a dispersion concentration *C*_m_ of 0.4%, fibers settle and are redispersed before sampling for the treatment. The two cavitation processes used (described in the next section) differ in the volume of the treatable sample. 1500 g of the *C*_m_ = 0.4% dispersion (6 g dry fiber equivalent) were used for the treatment using the BT device and 100 g of the *C*_m_ = 0.4% dispersion (0.4 g dry fiber equivalent) were used for the treatment using the UH.

### 2.2 Cavitation Devices: BT Device and UH

**Figure 1:**
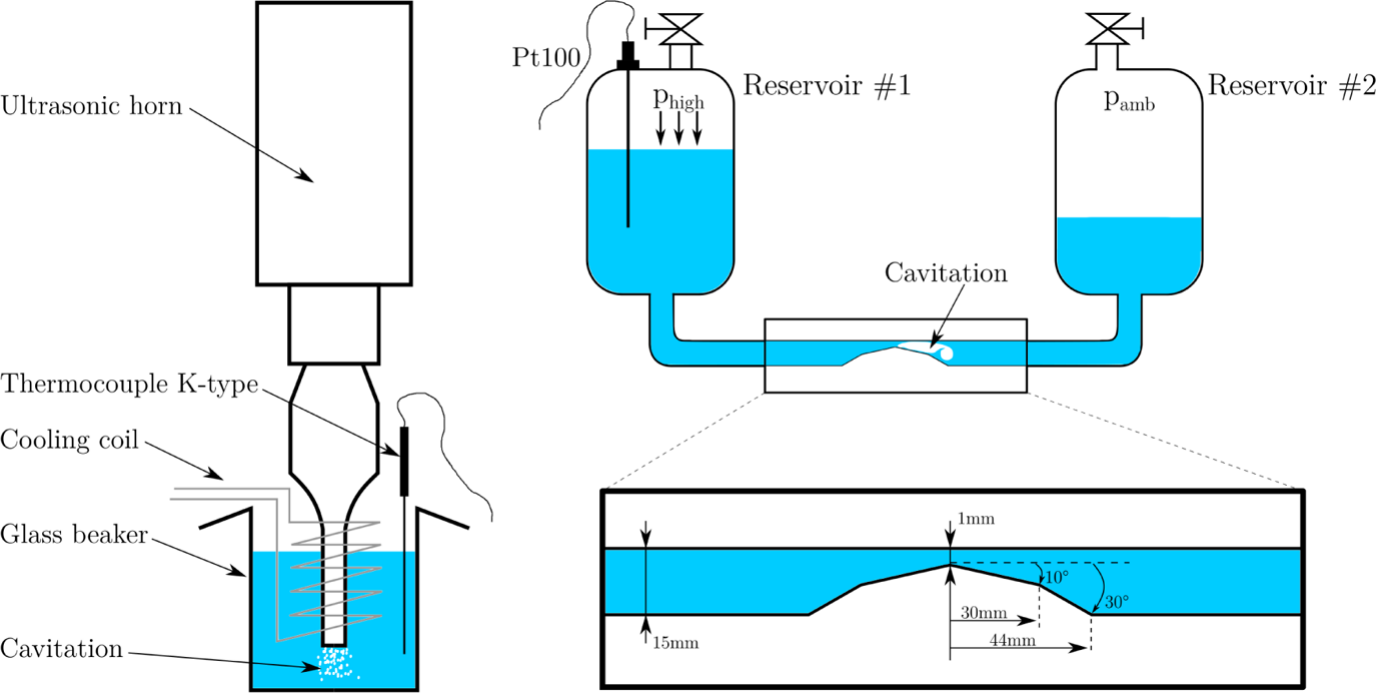
Cavitation test rigs. Left: UH. Right: BT. The close-up noted the dimension of the Venturi constriction-triggering cavitation.

Hydrodynamic cavitation was achieved using a BT device (Figure 1, right) consisting of two pressurize-able reservoir tanks of 2 L and a constriction of 1 mm by 5 mm in cross section.^46^ The investigated sample is oscillated between the tanks by applying pressurized air. The channel is optimized for fast pressure recuperation by having a double inclination (10 and 30°), which results in more intense cavitation bubble collapses.^47^ The applied air pressure, that is, drive pressure, sets the velocity and hence the cavitation intensity.^17^ We used 5 bar and 7 bar, where developed unsteady cavitation is achieved, resulting in high intensity cavitation cloud collapses, which are expected to have the biggest impact on the fibrillation of cellulose pulp samples. 7 bar is the maximum possible pressure used in the BT device, while 5 bar still provides enough energy input to achieve developed unsteady cavitation. The temperature was monitored using a resistance thermometer Pt100.

Ultrasonic homogenization (Figure 1, left) was performed using an ultrasonic horn (UH, ColeParmer 750W) having a tip diameter of 12.7 mm and an oscillation frequency of 20 kHz. For the experiments, the samples were filled into a 150 mL glass beaker with a diameter of 54 mm, resulting in a height of ca. 60 mm, including the UH positioned at the beaker center and a submerged heat exchanger coil to keep a temperature of 14 °C. The clearance of the UH tip to the beaker bottom was 34 mm. These parameters were not optimized but maintained constant throughout the experiments to guarantee comparability within this study. The cavitation intensity was controlled by setting the output power of the horn, which corresponds to the amplitude of the tip horn movement. In this study, 40% and 100% of the maximum horn power were used. The 5 bar in the case of a BT device and 40% in the case of an UH were chosen with the intention of operating in the same energy input range (for more details see chapter Section 3.1).

### 2.3 Case Settings and Case Overview

All types of pulp, DCe, ECe, CMCe, and TCe at a suspension concentration *C*_m_ of 0.4% and CMCe at a *C*_m_ of 2% were treated in the BT device with a differential pressure Δ*p* of 5 bar and with the UH at 40 and 100% power output. Additional experiments were performed with 0.4% CMCe in the BT device with a differential pressure of 7 bar and a longer treatment time with the UH. The fiber development was investigated for all samples. The fibril quality and chemical changes were only measured for a selected number of cases. The case settings and performed analytics are summarized in Table 1. The analytical methods and methods specific to sample preparation are detailed in the next section. The sample was stored in a refrigerator after the cavitation treatment until the analysis.

**Table 1:** Study Matrix of Cellulose Treatment by Cavitation and Performed Analytics

	fiber type	CMCe	CMCe	TCe	DCe	ECe
feed	concentration *C_m_* [%]	0.4	2	0.4	0.4	0.4
treatment	UH, 40% El. P.	yes	yes	yes	yes	yes
	time [min]	12.5	12.5	12.5	12.5	12.5
	UH, 100% El. P.	yes	yes	yes	yes	yes
	time [min]	5, 15, 30	5	5	5	5
	BT, Δp = 5 bar	yes	yes	yes	yes	yes
	time [min]	60	60, 105	60	60	60
	BT, Δp = 7 bar	yes	no	no	no	no
	time [min]	60				
fiber	length	yes	yes	yes	yes	yes
	LOM	yes	yes	yes	yes	yes
fibril	*NF*	yes	yes	yes	no	no
	*RSC*	yes	yes	yes	no	no
	SEM	yes	no	no	no	no
	AFM	yes	no	no	no	no
chemical	FTIR	yes	yes	yes	yes	yes
	XPS	yes	no	no	no	no
crystallinity	XRD	yes	no	yes	no	no

### 2.4 Measurement Methods

#### 2.4.1 Cavitation Characterization

We characterized the cavitation activity by high-speed imaging using a Photron Fastcam SA-Z at a frame rate of 75,000 fps and at a resolution of 1024 × 256 pixels. Illumination using a high-power LED lamp allowed a shutter time of 1 μs. The recording settings are explained in more detail in Petkovšek et al.^48^ The cavitation power was determined from the calorimetric measurements, tracking the temperature increase during the cavitation treatment. The BT cavitation device was externally insulated, while for acoustic cavitation, a thermo-insulated vessel was used instead of a glass beaker. Additionally, for the UH the consumed electrical power was measured using a power analyzer Norma 4000.

#### 2.4.2 Fiber Suspension Size Quality

The fiber length distribution was determined using a L&W Fiber Tester Plus (ABB, Sweden). The resolution limit is ca. 3 μm/pixel, hence missing the CNF fraction. We classified the fibers into length classes of [0.01, 0.5], [0.5, 1], [1, 2], and [2, 5] mm and compared their relative length-weighted contribution. The length distribution of all samples is presented in the Supporting Information. The CNF fraction or nanofraction (*NF*) was determined from size separation by sedimentation for 900 s in a centrifugal field of 1000 times the gravity *g*_0_. The top 46 mm from the suspension of 87 mm height in a centrifugation tube (VWR, SuperClear) was sampled. The suspension was diluted to 0.02%. Estimations (Supporting Information) indicate a sensitivity of the filament diameter on the centrifugation settings and suggest a filament thickness of *d* ≤ 200 nm for our settings. We determine the *NF* as a mass fraction of the solid material in the supernatant (*C*_supernatant_) to the mass fraction of the original suspension (*C*_m_) after drying at 160 °C
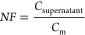
1The total charge *SC_total_* [μeq/g] of the fiber pulp was measured using conductometric titration.^49^ The surface charge *SC_susp_* [μeq/g] was determined by polyelectrolyte adsorption using streaming potential titration (Stabino, Colloid Metrix, Germany) following Wågberg et al.^42^ and compared to *SC_total_*
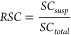
2

For all cellulose charges to be exposed in the suspension the relative surface charge (*RSC*) equals 1, which is the case for a fibrillation to the elementary fibril level. *RSC* is hence a measure of the specific surface area, respectively diameter, development.

#### 2.4.3 Fiber and Fibril Morphology

The fiber and fibril morphology were determined by three complimentary methods; light optical microscopy (LOM) (Olympus BX51, Olympus Corporation, Japan), scanning electron microscopy (SEM) (FEG SEM Hitachi S-4800, Hitachi High-Tech Corporation, Japan), and atomic force microscopy (AFM) (Multimode 8, Bruker, USA). LOM images were taken from a droplet of the suspension by adding a droplet of Safran red 1% in ethanol for staining. The dye adheres better to the charged fibers^50^ for which fibrils from CMCe and TCe were better visualized than from ECe and DCe. SEM images were taken of a diluted sample, following the preparation methods of Larsson et al.^51^ The diluted sample was filtered on the imaging substrate to ensure a good dispersion of the fibers and fibrils. For AFM imaging, the samples were diluted to 0.0025 or 0.005% and centrifuged at 2000*g*_0_ for 1 h to remove larger fragments. The supernatant containing the *NF*, namely nanocellulose fibrils (CNFs), was collected for further measurements. A mica substrate was freshly cleaved and functionalized using (3-aminopropyl) thriethoxysilane (99%, Sigma-Aldrich) by placing a 20 μL droplet on the cleaved side. After holding a droplet for 30 s, it was vigorously blown away by compressed air. Prior to sample casting, the CNF dispersion was dispersed using a Vortex Genie 2 (Scientific Industries Inc., USA) for 5 min. A 20 μL droplet of mixed dispersion was placed on functionalized mica and kept for 30 s until being blown away by compressed air. The substrate was left to dry overnight. Height images were collected for each sample in tapping mode in the air. At least 300 and 500 measurements were collected for diameters and lengths, respectively, using the softwares Nanoscope Analysis and ImageJ. Statistical analysis was performed using Origin 2021.

#### 2.4.4 Cellulose Crystallinity and Chemical Composition

The suspensions were frozen in liquid nitrogen and subsequently freeze-dried to remove all water for the measurement of the cellulose crystallinity and chemical composition using X-ray powder diffraction (XRD, Panalytical X′Pert PRO diffractometer, Malvern Panalytical, UK) and attenuated total reflection (ATR) Fourier-transform infrared (FTIR) spectroscopy (Spotlight 400 + Spectrum 100 FT-IR, Perkin-Elmer, UK), respectively. The calculations of the crystallinity index (*CI*) from XRD are presented in detail in the Supporting Information. We used two methods to calculate the *CI*: a deconvolution method using Cerro et al.’s^52^ amorphous model and comparing the peak areas *A*
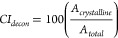
3and the more traditional but debated method of Segal et al.^53^ comparing the intensity of the highest peak (*I*_002_) and the local minimum *I*_min_ in the diffraction pattern 2θ range of 17–19°
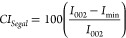
4

FTIR spectra were collected from five different locations of each dried suspension type (CMCe and TCe). ATR correction, background subtraction, and normalization on the band intensity located at 1030 or 1427 cm^–1^ were done for each spectrum. The normalized spectra of one type were averaged for further visualization.

For the quantitative analysis of the chemical degradation of cellulose upon different acoustic treatments of the CMCe pulp, X-ray photoelectron spectroscopy (XPS) using the Scienta-200 hemispherical analyzer and monochromatized Al Kα radiation of 1486.6 eV energy was used. All photoelectron spectroscopy measurements were carried out with a base pressure lower than 10^–9^ mbar. Dispersions were prepared in the similar way as AFM and sprayed with 20 pulses on silicia substrates cleaned in acetone and ethanol. Prior to spraying, each substrate was coated with Pt/Pd for 180 s, resulting in the formation of a thin conductive layer of ∼18 nm in thickness.

## 3 Fibrillation Results and Discussion

We first characterize the cavitation in the BT device and with the UH in Section 3.1. Following, we present the size development and fibrillation success in Section 3.2, and the change in crystallinity and chemistry in Section 3.3. In Section 3.4, we discuss our results, including conversion time scales of the UH batch treatment.

### 3.1 Characterization of the Cavitation and Cavitation Devices

**Figure 2:**
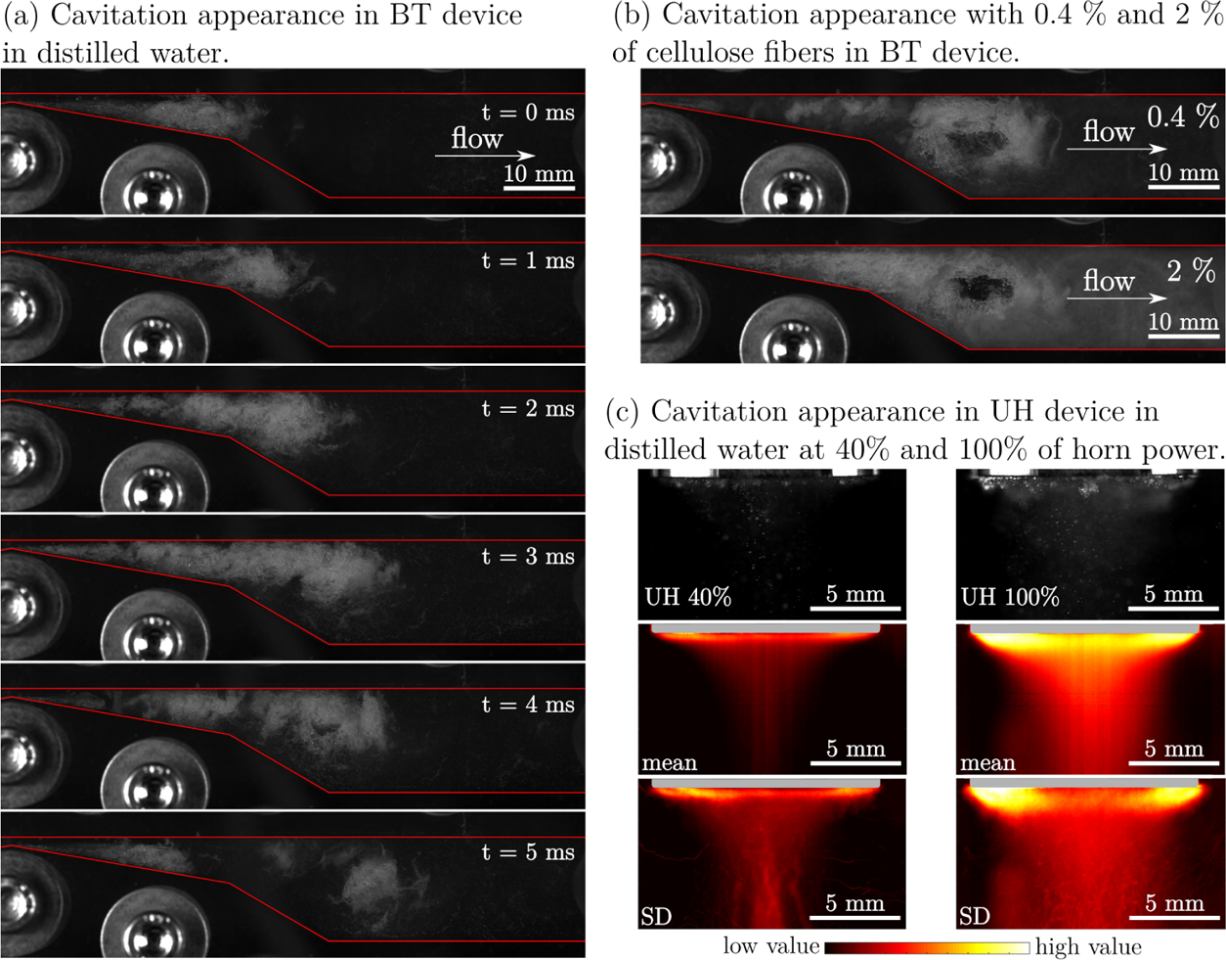
High-speed visualization of cavitation in the BT device at 5 bar pressure difference [(a) image sequence in distilled water and (b) instantaneous image of cavitation with addition of 0.4 and 2% of cellulose fibers] and the UH [(c) instantaneous image of cavitation—UH 40% and UH 100%, average appearance of cavitation—mean and standard deviation of cavitation appearance—SD].

We list the input power *P* in Table 2, the cavitation behavior in the BT device without and with fibers in Figure 2a,b and the cavitation active zone of the UH without fibers in Figure 2c. *P* was calculated from caloric measurements (Supporting Information). In the case of the UH, the presence of fibers decreased the suspension transparency and hindered the identification of cavitation bubbles. The frequency domain of the cavitation growth and collapse is presented in the Supporting Information, Figures S1 and S2.

**Table 2:** Power Input by Cavitation Treatment^a^

	Δ*p* (bar)	amplitude (%)	*P*_water_ [W]	*P*_FS,0.4%_ [W]	*P*_FS,2%_ [W]
BT5	5		13	12	15
BT7	7		21	19	
UH40		40	32	33	
UH100		100	115	112	

a The BT drive pressure Δ*p* [bar] and the UH amplitude as the percentage of the device’s maximum amplitude relate to the cavitation intensity. *P*_water_, *P*_FS,0.4%_, and *P*_FS,2%_ are the caloric cavitation powers for water, and the CMCe fibers suspension at *C*_m_ of 0.4 and 2% concentration, respectively.

In terms of the power input, we did not notice any significant difference between pure water *P*_water_ and the fiber suspension at *C*_m_ of 0.4% (*P*_FS,0.4%_) and 2% (*P*_FS,2%_). Also, the cavitation appearance in the BT device with fibers (Figure 2b) was comparable to water (Figure 2a), concluding that the presence of fibers did not alter the cavitation behavior significantly. With UH40, at 40% of the maximum amplitude, we aimed to match the power input from the BT device. The input power *P*_FS,0.4%_ of UH40 (33 W) is higher than for BT7 (19 W), but is in the same range. Different to hydrodynamic cavitation, the energy input in acoustic cavitation can be increased with the amplitude of the oscillating horn tip. The UH100 presents a treatment at the highest intensity.

The fiber treatment in the next section will be compared based on the specific energy input *E*, calculated from the input power *P* and the treatment time *t*. *E*_S_ is based on the sample mass *m*_sample_
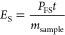
5

Aside from the cavitation power, the BT device and UH as used in this experiment differ in the treatment homogeneity and the bubble dynamics and size. In the BT device, all suspension passes through the cavitation zone (cavitation cloud volume estimated to 1.30 and 2.05 cm^3^ for 5 bar and 7 bar pressure difference, respectively) per cycle and is intensively mixed due to highly turbulent flow (Figure 2b). Differently, the UH is a batch process with an active zone^54,55^ below the horn and a dead zone elsewhere. The treatment uniformity, hence, depends on the suspension mixing, that is, the exchange between the active and dead zone. The active zone volume is estimated from visualization images (Figure 2c) and equals 0.060 and 0.129 cm^3^ for UH40 and UH100, respectively, which is a fraction of 0.6 × 10^–3^ and 1.29 × 10^–3^ of the total batch volume, respectively.

Frequency analysis based on visualization (Supporting Information, Figures S1 and S2) shows a distinct difference in cavitation event appearance between the BT and UH devices. In the case of the UH, main cavitation events occur at frequencies around 6 and 3 kHz for UH 40% and UH 100%, respectively, while in the case of the BT device, cavitation cloud shedding appears at much lower frequencies, around 200 Hz. In a BT device, the liquid accelerates passing through the channel’s constriction, causing the pressure to drop more gradually, resulting in longer cavity growth in terms of spatial and time domain. Bigger cavitation bubbles in the BT device are closely connected with cavitation dynamics; the largest frequency domains are up to a frequency of 200 Hz, which can be seen in Figure 2a as one 5 ms long cavitation cloud growth and detachment cycle. The ratio of cavitation events between the UH and BT devices speaks in favor of the UH having a 10 times higher number of cavitation events. When comparing maximal bubble size between the UH and BT devices, the BT device produces bubbles in a range of up to several 100 μm, while the individual bubble size in the UH does not exceed 100 μm (based on our visual observation^48^).

### 3.2 Fiber Size Development and Conversion into Nanocellulose

#### 3.2.1 Fiber Length Development and Fibrillation into Nanocellulose

**Figure 3:**
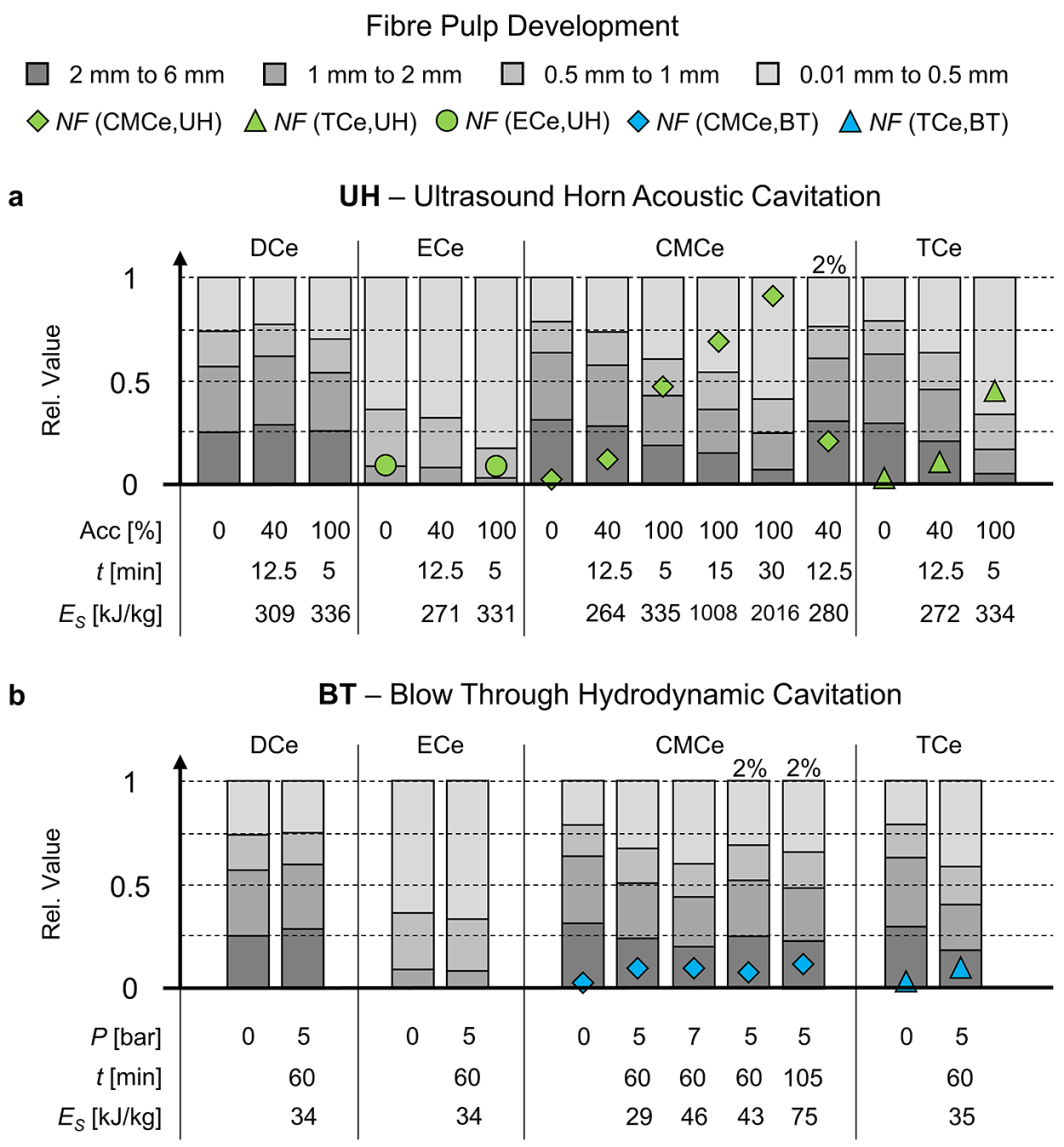
Summary of the fiber development, presented on hand of the fiber length contribution classified in long (2–6 mm), middle (1–2 mm), short (0.5–1 mm), and fiber fines (0.01–0.5 mm) fraction. The length classes are shown in gray with decreasing darkness, respectively. The corresponding fiber length distribution is provided in the Supporting Information. The *NF* (diamond, triangle, and sphere symbols) states the weight-based nanocellulose fraction. The treatment intensity *P* or Acc, treatment time *t,* and applied energy *E*_S_ are stated per case. a: summarizes UH cases and b: summarized BT cases.

**Figure 4:**
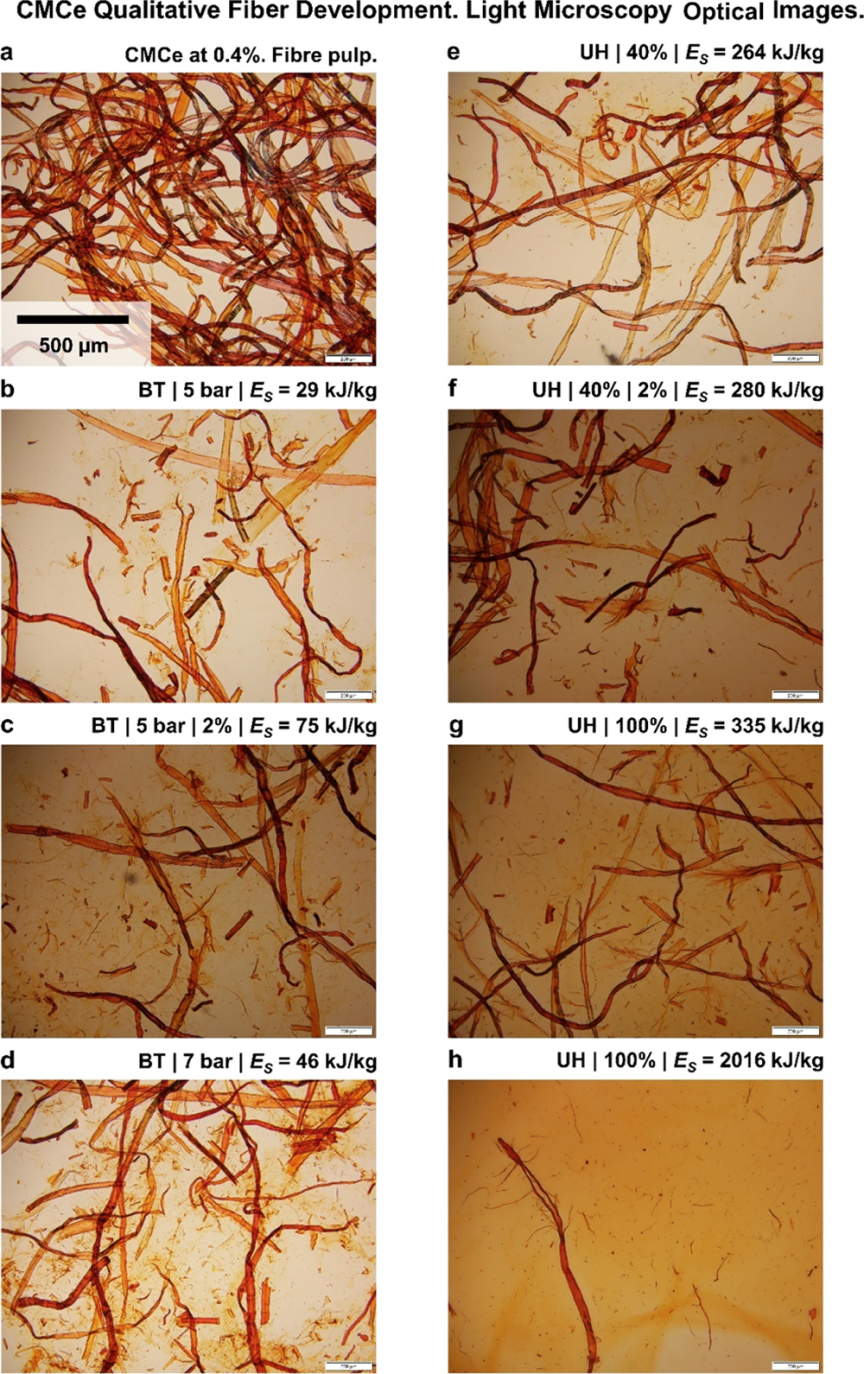
LOM images of CMCe and TCe pulps after the UH and BT treatments. The magnification is 10× for all the images shown. a: includes the scale bar of 500 μm, which is the same for all images.

In Figure 3 we summarize the fiber length development and conversion into nanocellulose from the UH (Figure 3a) and BT (Figure 3b) treatments. The nanocellulose fraction *NF* is given by the symbol (a diamond for CMCe, a triangle for TCe, and a circle for ECe). The *NF* after treatment was insignificant for DCe and ECe. The length distribution presents the composition of the fiber phase, that is, the fraction 1 – *NF*, classified into four length fractions. Three aspects are apparent from the comparison in Figure 3: (1) a clear benefit from weakening the fiber on an inter-fibril level by chemical modification (CMCe and TCe) resulting to an increase of the fine fraction (light gray) and the *NF*. DCe, which is the unmodified cellulose pulp, and ECe sustained the cavitation with the exception of UH100 on ECe, where the fine fraction increased but not the *NF*. The UH100 treatment of DCe was impacted by fibers stapled to the cooling coil, resulting in a poorer mixing of the fiber, whose extent and impact on the treatment could not be evaluated. Complementing LOM images of DCe, ECe, and TCe are presented in the Supporting Information, Figures S28–S48. (2) Results for *C*_m_ = 2% suspension after BT5 and UH40 treatments are comparable to the lower concentration of 0.4%, with a slightly higher *NF* for 2% after the UH40 treatment. Given the higher total mass, the process is more efficient for the fragmentation of the fiber into fiber fines and an initial fibrillation into nanocellulose. However, the UH100 treatment of the 2% suspension needed to be aborted after ∼3 min, when a fiber-containing gel formed that could not be mixed by the cavitation bubbles. Instead, we noticed vapor/smog rising from the sample, suggesting a strong local heating. We conclude that cavitation bubbles are not strong enough to yield a nanocellulose gel, for which the UH needs to operate at a lower concentration, where the connectivity threshold can be used for guidance.^44^ (3) UH40 is qualitatively comparable to BT5 and BT7, albeit its total energy input of UH40 is higher. Subtle differences for CMCe are a larger increase in the fine fraction for BT and the *NF* for UH40. We will compare the product quality from the UH and BT for CMCe next on hand of LOM images (Figure 4).

BT hydrodynamic treatment fragmented fibers in the axial and radial direction (Figure 4b–d) and produced coarse fibrils with a length in the order of 100 μm, for low and high concentrations. The treatment at a higher intensity (BT7, Figure 4d) was qualitatively comparable, albeit at an apparent stronger treatment and with a larger production of coarse fibrils. The BT process is comparable to the recently documented fibrillation in short microchannels.^6^ In microchannel fibrillation, fibers are initially fragmented by tension forces arising during the acceleration. We estimated the acceleration and velocities for BT (Supporting Information) and find these low compared to microchannel flows.^6^ Therefore, we can exclude fragmentation by tension for BT. The treatment effect in BT can be accounted to cavitation damaging, which appears inefficient for the fibrillation of cellulose fibers into CNFs.

The UH was efficient in developing the fiber into smaller fragments and nanocellulose (Figure 3a). After the UH40 and UH100 treatments, the suspension consists of untreated fibers and fiber fragments in the order of 100 μm and coarse fibrils, that is, material <100 μm (Figure 4e–h), which appears to be smaller than what was noted for BT (Figure 4b–d). The morphology of some fiber fragments resembles fiber ripped in half. Furthermore, only for UH100, long and coarse fibrils were observed that appeared as fragments from fibers that were sliced in an axial direction. That points not only toward a more intense treatment from increasing the sonication power but also to a change in the fiber fragmentation mechanism with the treatment intensity from UH40 to UH100. Continued UH100 treatment for 30 min, largely fibrillated all the CMCe (Figure 3a). Still, some fibers that are comparable to the pulp fibers can be found, which confirms for us as an inhomogeneous treatment suffering from the mixing within the batch. We will discuss that in more detail in Section 3.4.

#### 3.2.2 Fibril Quality Development

**Figure 5:**
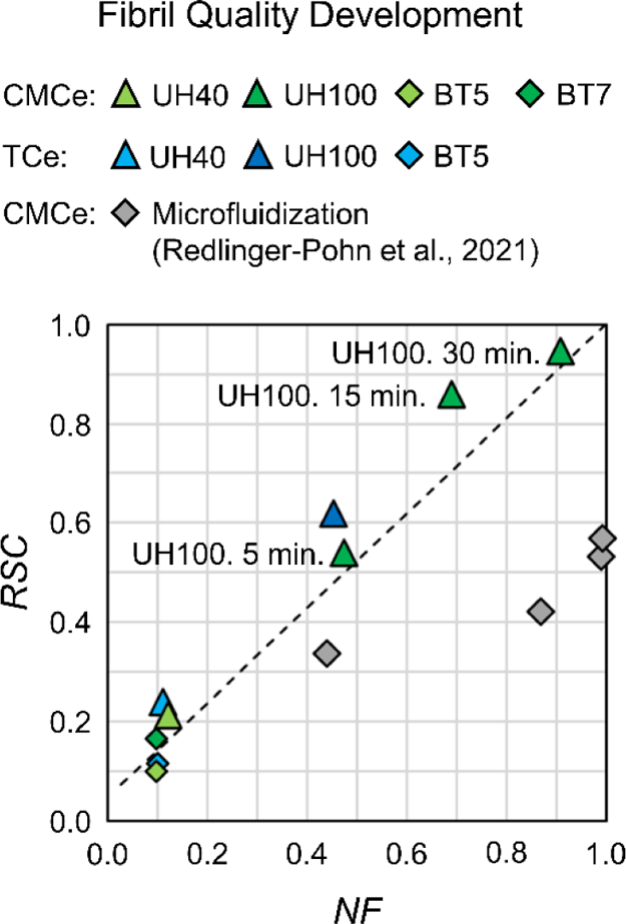
Fibril surface charge development *RSC* in comparison to the nanocellulose fraction development *NF*. The dashed lines represent a linear increase from the CMCe pulp value to the maximum development [1, 1]. Triangles are the UH treatment and diamonds are the BT treatment with CMCe in green and TCe in blue. Gray diamonds are microfluidization results from Redlinger-Pohn et al.^6^ in comparison.

**Figure 6:**
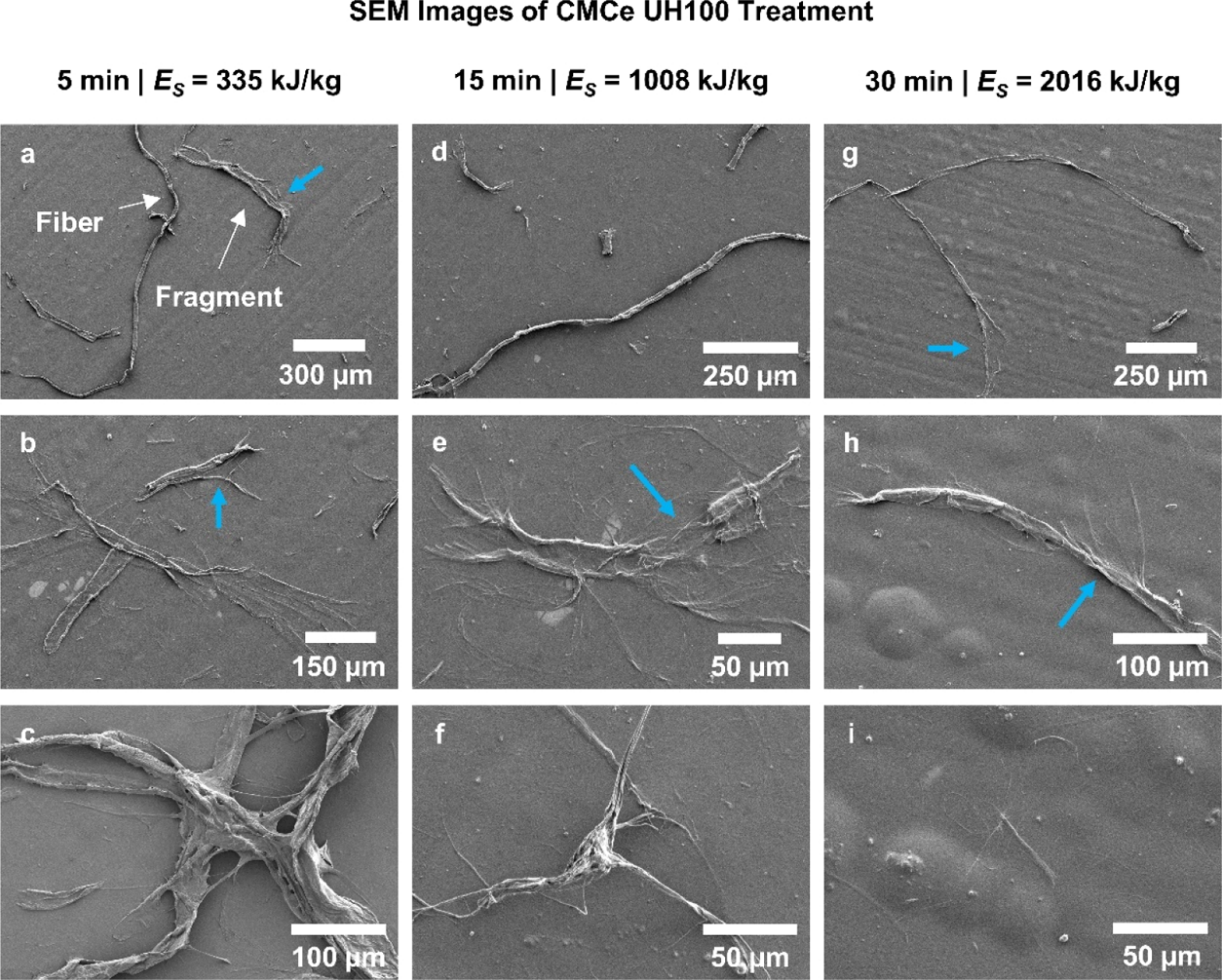
CMCe UH100 treatment SEM images highlight the fragmentation damage on the fiber and the morphology of the fragments and macrofibrils. (a–c) 5 min UH100 (*E*_S_ = 335 kJ/kg). (d–f) 15 min UH100 (*E*_S_ = 1008 kJ/kg). (g–i) 30 min UH100 (*E*_S_ = 2016 kJ/kg). More images per case are provided in the Supporting Information. The blue arrows mark the speculated bending damage discussed in Section 3.4.1.

In Figure 5 we compare the development of the nanocellulose mass fraction *NF* to the development of the measured surface charge *RSC*; both are bulk values describing the sample average. The *NF* is set by the centrifugation method with coarser fibrils, that is, diameters in the order of 100 μm included. *RSC* measures accessible charges, which are located at the elementary fibril surface for CMCe with a SC_total_ of 600 μeq/g.^56^ The linear increase of *RSC* with *NF* for the UH treatment can, hence, be explained by the efficient and direct fibrillation of the cellulose fiber and fiber fragments into nanocellulose at the elementary scale. That is unique for sonication treatment, and differs, for example, from microfluidization, for which Redlinger-Pohn et al.^6^ recently documented the limitation leading to the production of fibril aggregates, i.e., incomplete fibrillated cellulose fibers captured by *RSC* being below the *NF* (Figure 5, gray diamond). At intermediate *NF*, *RSC* for CMCe and TCe and UH are slightly above the linear increase (dashed line), corresponding to an opening of the cellulose structure in the remaining fibers and fragments that are not accepted by the *NF*. Fitting this argument, we have noted in the LOM images (Figure 4e–h, Supporting Information), branched and open CMCe fibers and fragments, which we give a closer investigation using SEM as shown below in Figure 6.

For BT5, the *NF* and *RSC* are in the order of the pulp material. Fragments and fines were observed using LOM (Figure 4b,d). These fragments are however too large to be counted in the *NF*, and also the increase in the accessible surface from fragmentation is small, for which *RSC* is small. Interestingly, *RSC* increases from BT5 to BT7, while the *NF* is nearly constant. LOM images show a larger quantity of fiber fragments for BT7 (Figure 4d) compared to BT5 (Figure 4b). These fragments hence need to be large, that is, thickness >200 nm, for which they are excluded from the *NF*, but in their total number they contribute to an increase in the total accessible surface area in the suspension (shown in the following section). This adds to the previous observation that hydrodynamic cavitation is poor for fibrillation, while it may be capable to some extend of fragmenting the fibers to smaller sizes, as also seen in the increase of the fine fraction (Figure 3b, CMCe and BT7).

#### 3.2.3 Development of the Fiber and Fibril Morphology

In Figure 6 (and Supporting Information Figures S49–S55), we present close-up SEM images of the CMCe fibers development from the UH100 treatment. For UH100 after 5 min (Figure 6a–c), the population of fragments is diverse, including fragments from radially broken fibers, that is, pieces with a cylindrical shape, axial broken fibers that appear as fragments from the cell wall, aside from nearly untreated fibers. Many of the fragments are bifurcating at the ends of larger strains and resemble the shape of tree branches that were broken from excessive bending. The number of fibers and the size of fiber fragments was greatly reduced after 15 min (Figure 6d–f), yet undamaged fibers were still present. The fragments, an example shown in Figure 6d, are branched with longer fibrils but still connected to the main body of the fragment. Figure 6f presents an extreme case of coarse fibril strains connected to a main body and splitting up toward their ends. It is an example of a particle being large enough to sediment in the centrifugal field for which it is not represented in the *NF* but having an accessible surface area that is large compared to a pulp fiber. Such particles are then measured to have a *RSC* > *NF*, which we believe results in the *RSC* overshoot documented in Figure 5, for an intermediate UH100 treatment after 15 min. The trend is continued and images after 30 min (Figure 6g–i) are qualitatively comparable; the number of fibers and fragments is reduced, yet untreated fibers are present. The fragments are generally smaller and more fibrillated and covered by a film of the CNF, which was *NF* = 0.9.

The presence of approximately 10% fibers and fragments after 30 min sonication can result from a material difference, that is, different strengths of the fiber pulp mixture, from the batch sonication or a combination of both. Observed remaining fibers exhibit some localized damage on their surface, but appeared mostly untreated (Figure 6a,d,g). Such resistance against mechanical damage at, for example, a first exposure to the cavitation treatment followed by a fragmentation and fibrillation at a consecutive cycle is little plausible. It is more likely that the decrease in the fibrillation rate and the presence of fibers after 30 min UH100 treatment results from the process settings, that is, a small active zone and the mixing of the fiber material between the batch and the active zone. We will discuss this further in Section 3.4.2.

**Figure 7:**
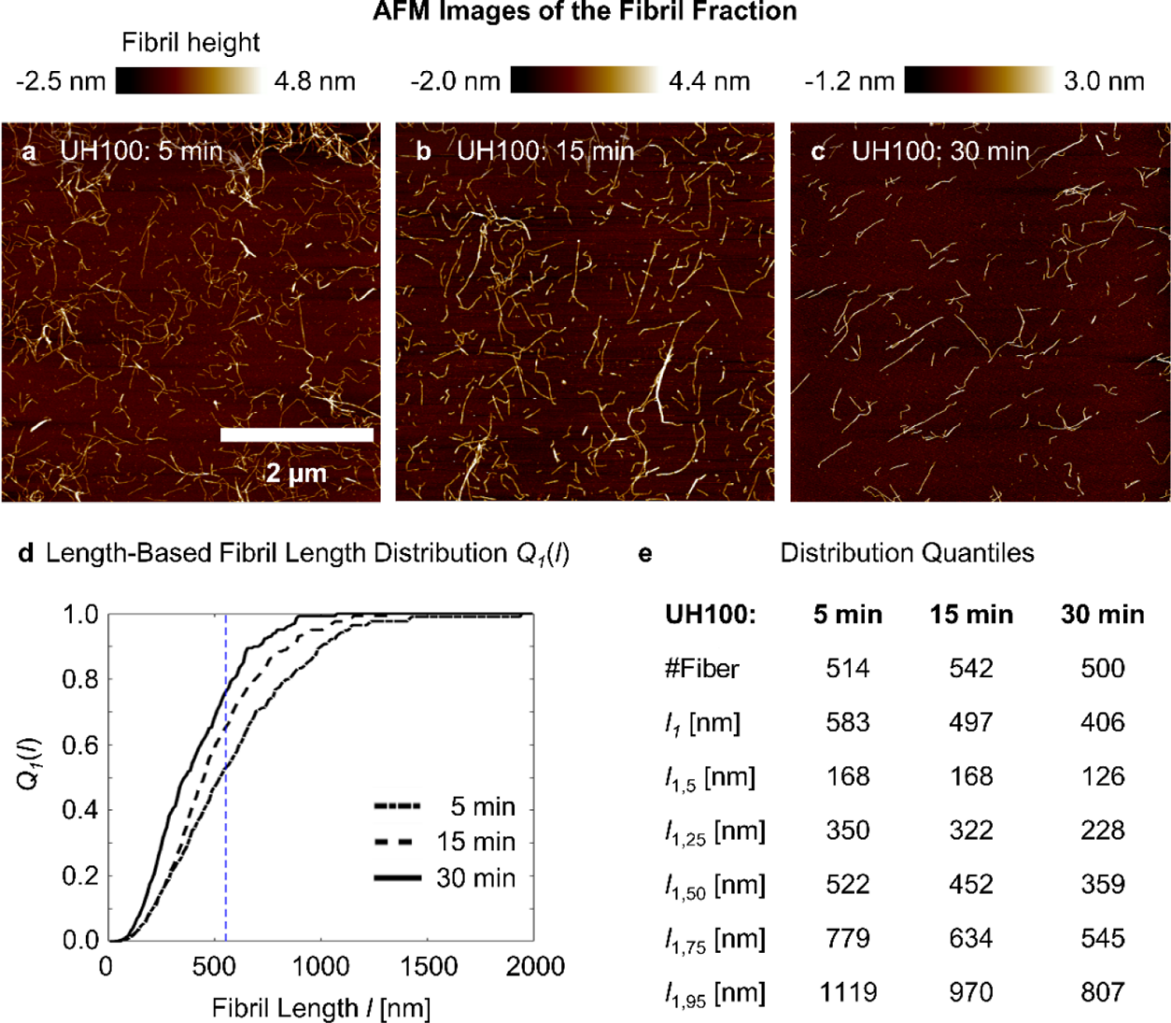
Fibril fraction morphology and length distribution from CMCe UH100 treatment. (a–c) Exemplarily AFM image after 5, 15, and 30 min treatment, respectively. (d) Length-based cumulative fibril length distribution. Marked as dash blue line is the fibril length *l* = 522 nm, which is the median length of UH100, 5 min. (e) Quantile of the length distribution, stated as the second number of the index. *l*_1_ is the mean length-based fibril length.

In Figure 7a–c we present the morphology of the fibril fraction of CMCe after UH100 treatment, separated from the coarser material by centrifugation at 2000*g*_0_ for 1 h. The AFM height images were used to determine the length and height distributions (details in the Supporting Information). Our observation shows that the morphology of the fibrils does not change significantly. After 5 to 30 min treatment, they appear individual, slender, and with kinks, a shape expected for the elongated CNF. In Figure 7d, we show the associated cumulative length-based fibril length distribution *Q*_1_(*l*) and highlight in Figure 7e the distribution characteristics, with *l*_1_ being the length-weighted mean fibril length and the index numbers being the distribution quantile. The fibril diameters are comparable for all the cases, with an arithmetic mean of ∼2.5 nm (Supporting Information, Figures S4–S6). That adds to our interpretation of Figure 5, of cellulose fibers and fragments being fibrillated to the elementary thickness scale. The mean fibril length *l*_1_ decreases with the sonication time, which agrees with previous observations.^9,30^

Interesting is a separate look at the shorter and longer fibril fractions. Initially, the fibril fraction contains very long fibrils at small number, which disappear with continued treatment. Furthermore, the fraction of the longer fibrils decreases, and, for example, the median length after 5 min, *l*_1,50_ = 522 nm, is approximately the 75% quantile after 30 min, that is, 25% of the fibrils are longer. The shorter fibril fraction initially changes little from 5 to 15 min, and decreases slightly from 15 to 30 min. It is important to reflect, that the fibril length distribution at every sampled time is the result of newly produced fibrils (increase of the *NF*) and the length reduction of existing fibrils. Once the fibers are fibrillated, that is, *NF* ∼ 1, only shortening of the existing fibrils takes place until reaching a limiting length. We find the population of fibrils with lengths of 100 nm increasing, but no further shortening, which is comparable to the findings of Zhou et al.^9^

### 3.3 Crystallinity and Chemical Structure

#### 3.3.1 Development of the Cellulose Crystallinity

**Figure 8:**
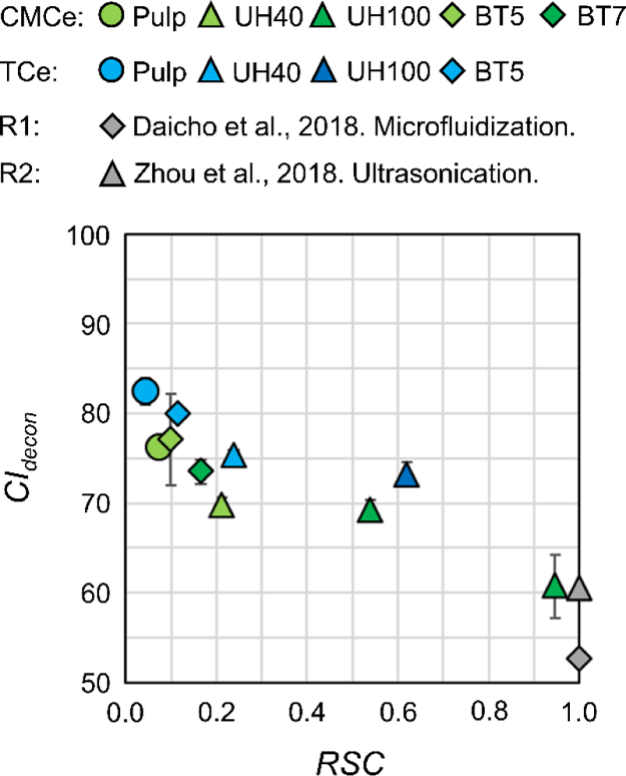
*CI* from convolution, *CI_decon_*, after Cerro et al.^52^ presented over *RSC*. Reference values from Daicho et al.^57^ (R1: gray diamond) and Zhou et al.^9^ (R2: gray triangle) for their cellulose fibrils.

In Figure 8, we present the *CI* calculated by deconvolution of the diffractogram following del Cerro et al.^52^ (*CI_decon_*) over *RSC*. The XRD diffractograms and method descriptions are provided in the Supporting Information as is the *CI* calculated with the method of Segal et al.^53^ (*CI_Segal_*) for comparison.

We find a negative correlation of the *CI* with the accessible surface, quantified by *RSC*, which is consistent with Daicho et al.,^57^ who accounted the decrease of TEMPO cellulose crystallinity to surface defects from fibrillation. This was recently confirmed by Mudedla et al.,^58^ who demonstrated that exposed charges locally affect the cellulose polymer structure in a fibril. The *CI* and *RSC* are both bulk values and hence reflect the mixture of cellulose fibers and cellulose fibrils, which are of elementary thickness after ultrasonication. We hence interpret the linear decrease of *CI_decon_* with *RSC* (Figure 8) as their codependence on the number of charged groups exposed to the environment, i.e., water, which increases with processing, i.e., ultrasonication, time. The final *CI_decon_* and *CI_Segal_* at a high degree of fibrillation,i.e., *RSC* ∼ 1, is comparable to the literature values of, for example, Zhou et al.,^9^ who also used ultrasonication, and Daicho et al.,^57^ who used homogenization techniques and the TEMPO-oxidized pulp at, however, a higher charge of 1400 μeq/g. This comparability of our *CI* to cellulose fibrils after noncavitating fibrillation suggests that the prevailing harsh conditions in ultrasonication, i.e., hot-spots of 1000 K and hydroxy radicals (^•^OH), did not affect the cellulose crystallinity significantly.

#### 3.3.2 Chemical Modification from Cavitation

**Figure 9:**
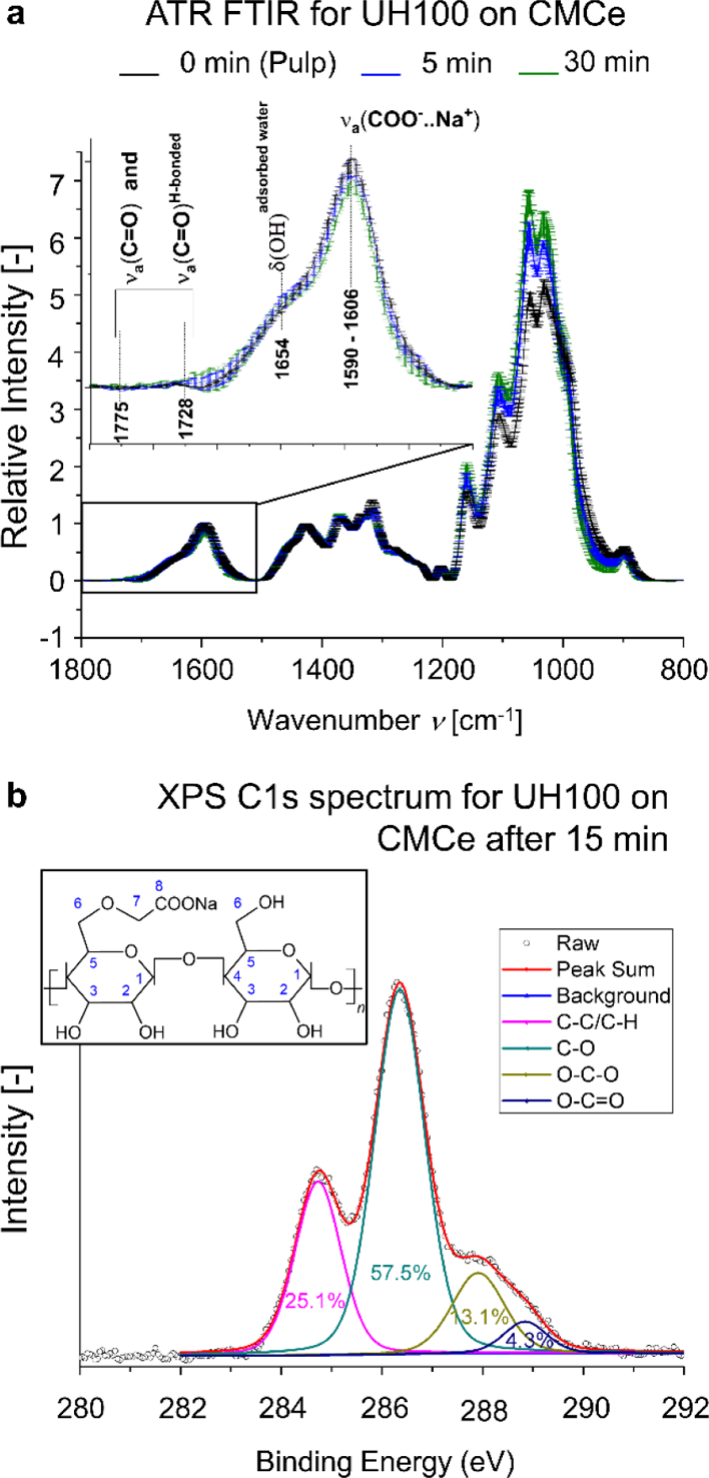
Polymer composition of CMCe after ultrasonication. (a) ATR FTIR spectra of the CMCe pulp before and after UH100 5 min and 30 min treatment. (b) Typical XPS C(1s) high resolution spectrum of the CMCe pulp after UH100 15 min treatment. Band deconvolution demonstrates the composition of corresponding C(1s) groups, which can be visualized from the formula of the CMCe monomer in the top left corner.

Possible chemical modifications, for example, oxidation, of the cellulosic pulps were probed with ATR FTIR and for CMCe also using XPS. The ATR FTIR spectra are presented in the Supporting Information (Figures S22 and S23, with the band vibrations explained and referenced in Table S3). The results for CMCe from the UH100 treatment are shown in Figure 9a. The CMCe polymer structure is shown in the insert of Figure 9b. Note that, the representation of every second glucose unit to be derivatized is an exaggeration for a concise representation. The first glucose polymer C^6^ hydroxyl group formed an ester with a carboxymethyl group. The second glucose polymer C^6^ hydroxyl group is native. An oxidation of this C^6^ hydroxyl group would result in the formation of carbonyl groups that appear at 1728 and 1775 cm^–1^ in the ATR FTIR spectra.^59,60^ The corresponding bands were not detected in the studied cellulose pulp independently of cavitation energy or duration (Figures 9a and S23) for which we can exclude an oxidation of the cellulose by radicals formed during cavitation for our cases.

With XPS, we studied the surface (i.e., a couple nanometers deep) of films formed with the CMCe dispersion before and after UH100 treatment. Representative high-resolution spectra are shown in Figure 9b (and Supporting Information, Figure S24), and the deconvolution of the bands for the carbon C(1s) signal is summarized in Table 3. CMCe can be described by four C(1s) band groups: (1) C–C and C–H, (2) C–O, (3) O–C–O and C=O, and (4) O–C=O.^61,62^ Unfortunately, no homogenous thin film could be produced from untreated CMCe and UH100 treatment for 5 min as these samples contained a larger fraction (i.e., 1-*NF*) of nonfibrillated material, which resulted in an artificial high signal of group (1). Furthermore, XPS is a surface technique probing only a few nanometers in depth, for which organic contamination and cellulose degradation from radiation can increase the contribution of group (1).^62^ Given the uncertainties of the group (1) contribution, we will not discuss implications from the absolute value but only the relative contribution of group (2) to the sum of group (3) and group (4).

**Table 3:** Band Area and Their Ratio for Different CMCe Groups for the C(1s) Signal from the Signal Deconvolution^a^

sample	ratio of the band area for C(1s) signal					fiber ends
CMCe, UH100	group (1) C–C, C–H	group (2) C–O	group (3) O–C–O	group (4) O–C=O	ratio (2)/((3)+(4))	*n*_End,rate_^*^
pulp	0.543	0.269	0.112	0.076	1.4	
5 min	0.526	0.320	0.153	0	2.1	1.21
15 min	0.251	0.575	0.131	0.043	3.3	1.93
30 min	0.195	0.624	0.169	0.012	3.5	1.50

a *n*_End, rate_^*^ is an estimated relative number of fiber ends calculated with the AFM measured length distribution (Supporting Information).

Group (2) represents C–OH (hydroxyl groups) and C–O–C. Group (3) consists mostly of O–C–O, which can represent C^1^ in the cellulose chain and C^8^ in the carboxylic group formed after carboxymethylation. The number of O–C–O groups at C^1^ could change during cavitation if hemiacetal groups at the chain ends, that is, C^1^, are oxidized. An aldehyde formed as an oxidation product at C^1^ would give a corresponding carbonyl vibration at FTIR, which we did not observe, as presented earlier. Group (4) was associated to the presence of small portion of protonated carboxylic groups, which may remain at neutral pH conditions at which acoustic cavitation was performed.^41^ Chen et al.^63^ and Zhou et al.^33^ recently published that sonication does not decarboxylate, for which the sum of group (3) and group (4) will remain constant. We hence can attribute the increase in the ratio of group (2) to the sum of group (3) and group (4) (Table 3) to the formation of hydroxyl groups at new chain ends from hydrolyzation of the β-glycosidic bonds between C^1^ and C^4^ (Supporting Information, Figure S25b). Interestingly, the relative contribution sum of group (1) and group (2) is constant ∼0.82 and the sum of group (3) and group (4) is constant ∼0.18, which is in the range of the estimated degree of substitution for the polymer chains at the fibril surface (Supporting Information) being 0.12–0.14, using the cellulose fibril cross-sectional model of Rosén et al.^64^

Breakage of the β-glycosidic bonds needs to happen during the formation of kinks, i.e., deformations of the fibril in the length direction,^34,65^ and breakage of cellulose fibrils into smaller fibrils (see the length distribution in Figure 7d), facilitated by hydroxyl radicals that form at the hot spot of an imploding cavity.^66,67^ The increase in the group (2) ratio is most prominent for early cavitation, i.e., until 15 min, and flattens out thereafter. Zhou et al.^33^ reported a reduction in the number of kinks, and the ceasing of the fibril shortening with the sonication time. For comparison, we calculated the relative increase of the fiber ends *n*_End,rate_^*^ with the processing time from our AFM data (Supporting Information), assuming an initial fibril length of ∼1100 nm (i.e., *l*_1,95_ at UH100 after 5 min, Figure 7e). *n*_End,rate_^*^ decreases after 15 min with processing time, and the qualitative agreement supports our argumentation of the ceasing formation of hydroxyl groups. The absolute change of *n*_End,rate_^*^ differs compared to the change of group (2), and we show that *n*_End,rate_^*^ depends on the assumed fibril length in the fiber (Supporting Information) and does not capture hydrolysis from kink formation at the early stages of sonication.

### 3.4 Discussion

#### 3.4.1 Cellulose Fiber Cavitation Fibrillation

**Figure 10:**
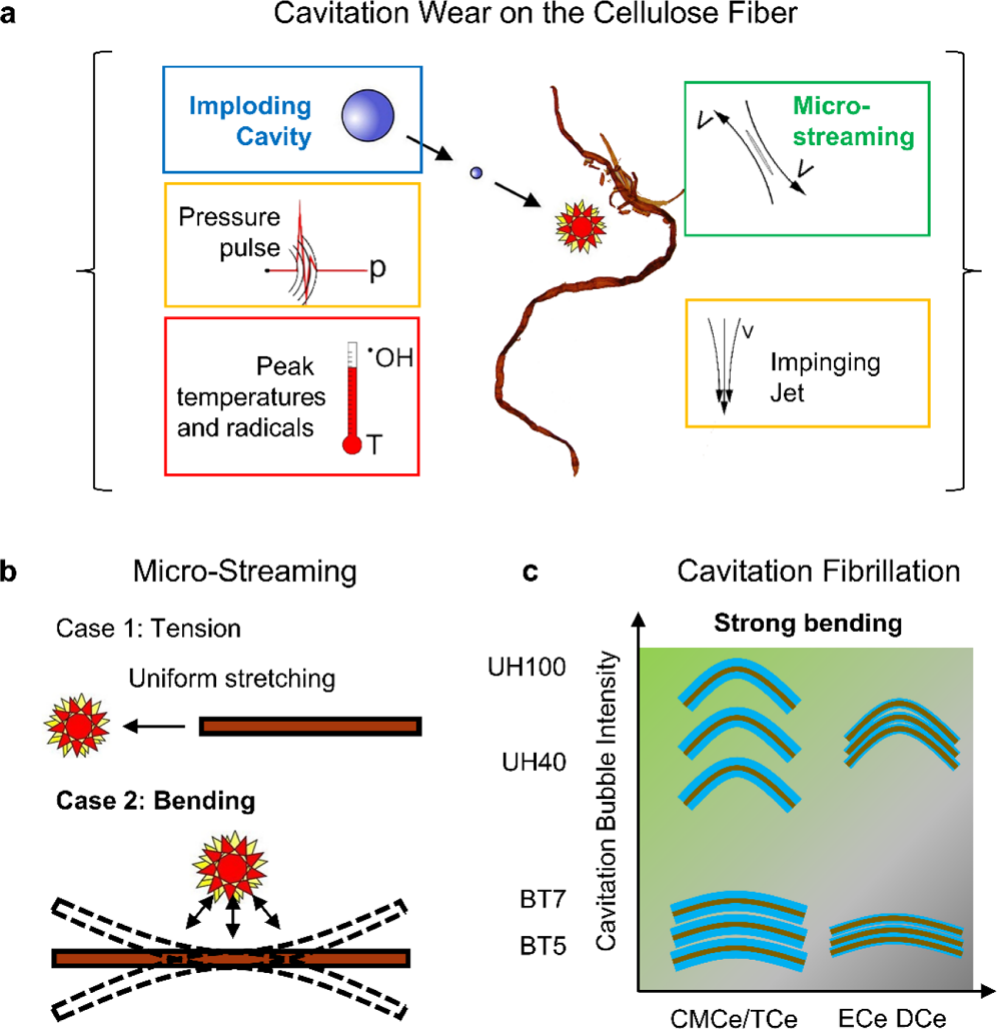
Cavitation fibrillation of the cellulose fiber. (a) Cavitation mechanism acting on the cellulose with microstreaming highlighted as most plausible action. (b) Microstreaming can stretch or bend the cellulose fiber and fibril.^35^ (c) Fibers are fibrillated at a large bubble intensity, that is, number and collapse frequency, and low inter-fibril cohesion, which is indicated by a thicker blue line.

Cavitation is the rapid phase change from liquid to vapor and back to liquid (vaporization and condensation process) at small scales, i.e., micrometer, creating a local harsh environment of >1000 K temperature and hydroxy radicals (^•^OH), pressure waves, high velocity impinging jets upon asymmetrical collapse of the cavity, and microstreaming, i.e., hydrodynamic drag, as illustrated in Figure 10a. High temperatures and radicals can degrade the glucose polymer,^68^ which we did not observe, aside from a possible hydrolysis that may assist (or result from) the cellulose fibril breakage. Local high temperatures and chemical radicals can hence be excluded as leading mechanisms in cellulose pulp fiber fibrillation by cavitation.

Mechanical effects such as pressure waves could induce local deformations and are discussed as damage sources for closed particles, i.e., microorganisms.^69−71^ Cellulose fibers are, however, open porous structures, with pits designed to transport fluid.^72^ The expected damage from pressure differences is an explosion rupture, differing from the dominant damage type observed (Figures 4, 6 and Supporting Information). Rapid pressure changes may be the cause of fiber modification,^19−21^ including local modifications of the open pores, i.e., pits,^73^ but they are regarded of minor importance for fiber fibrillation by cavitation.

Impinging jets would locally abrase the surface, forming pit holes,^74^ a form of damage that we did not observe on our fibers (Figures 4, 6 and Supporting Information). Furthermore, impinging jets are formed by asymmetrical bubble collapses,^74^ for example, the formation and collapse of a cavity at the fiber surface. Cavities are seeded by dissolved gas nuclei,^75^ which were shown by Ajersch and Pelton^76^ to form preferably in the bulk or on air-filled pockets, but not on the cellulose fiber hydrophilic surface. We hence speculate, that cavitation bubbles will mostly form in the bulk but not on the fiber for which a symmetric collapse is more likely. The effect of the surface tension on the cavitation bubble collapse is, however, largely unexplored. From the damage observed on the imaged fiber and the literature-based speculation, we expect impinging jet damage to be of minor importance in cellulose fiber fibrillation by cavitation.

Microstreaming describes localized hydrodynamic forces created by the fast fluid flow inward the collapsing or outward the growing cavity,^30,32,35^ the consequences of which can be stretching and bending based on the orientation of the bubble toward fibers and fibrils (Figure 10b). A recent numerical study on bubbles–liposome interaction^71^ identified stretching as one of the possible destruction mechanisms. For long, and hence high-aspect ratio, carbon nano-tubes (CNTs), Pagani et al.^35^ documented a tangential orientation to the growing cavity and bending into the cavity rather then re-orientation into the collapsing cavity. CNFs and CNTs are of comparable size and smaller than the cavitation bubble, which can reach diameters of several 100 μm. Differently, the cellulose fiber thickness is in the order of the cavity size and the fiber length exceeds the scale, for which several cavity growth and implosion events along the fiber axis are conceivable. It is hence unlikely that the cellulose fiber rotates or translates as a whole in response to a growing or collapsing cavity, but may react locally by bending within the shear field of a cavity, as we sketch in Figure 10b. Imaged fibers, captured in Figures 4 and 6, do exhibit damage expected from bending failure (imagine the ductile damage on a young tree’s branch from bending). For example, the fragment’s ends in Figure 6e are bifurcating, comparable to a successful fragmentation by bending, and the fragments in Figure 6b and fibers in Figure 6g depict localized damage from an unsuccessful fragmentation by bending.

At the CNF scale, understanding of the CNT fragmentation can be applied by considering the impact of the material stiffness and bubble collapse velocity, which may offset the length discrimination between bending and rotation in the collapsing cavity. Furthermore, cellulose fibrils are ductile materials, and bending may not initially lead to breakage but can induce deformation failure, such as delamination^6,77^ or the formation of kinks.^34^ Kinks are directional changes in the fibril axis direction, which were previously shown to result from processing,^34^ and the bending of fibrils was demonstrated to damage their structure on a molecular level.^65^ Recently, Zhou et al.^33^ tracked the number of kinks and the length of TEMPO-CNF with sonication exposure, finding both decreasing. Bending deformation in the flow field of the collapsing cavities is comparable to the bending deformation by turbulent eddies,^6,78,79^ at, however, differing intensities. Bending scales in microfluidization with the turbulent eddy size,^79^ which was estimated by Redlinger-Pohn et al.^6^ to ∼110 nm, whereas Pagani et al.^35^ report a curvature radius of 40 nm for the modeled CNT bend by an imploding cavity. Considering fibrillation as a bending failure, it is then not surprising that for the same material, sonication is more efficient to develop the fibril surface and thickness, than microfluidization (Figure 5). As a difference to the dilute case with individual CNT described by Pagani et al.,^35^ and which we estimated for fibers with a typical aspect ratio of 100 for *C*_m_ = 0.4%, the aspect ratio of the longer fibrils is higher, calculating for *l* = 800 nm (Figure 7e) and a thickness of 2.5 nm (Supporting Information) to 320. For that, the CNF interaction and the fixation of individual CNF within a network can be expected,^44^ reducing the chance of the CNF to orientate into the collapsing cavity and increasing the probability of bending-induced breakage.

From our analysis, we consider microstreaming as the leading mechanism for the fragmentation and fibrillation of cellulose fibers into CNFs by cavitation. Thereby, we suspect bending and delamination failure^6,77^ to be dominant in the initial cellulose fiber fibrillation. A direct observation was unfortunately prevented by the opaque nature of the suspension with fibers at *C*_m_ of 0.4% and cavities (vapor bubbles). As a follow-up, we suggest in future work a close-up study of the fiber fibrillation by single, small number of cavities which then allows an optical investigation. To detail on the fibril shortening mechanism, we suggest following Pagani et al.,^35^ who documented different rates in the CNT length reduction for bending and scission-induced breakage. The length of the long and initial (close as possible) monodisperse CNF^80^ can be tracked with sonication time.

#### 3.4.2 Cellulose Type and Cavitation Treatment

Although CMCe and TCe fiber pulps were well fibrillated by sonication, DCe and ECe did not fibrillate into CNFs, and hydrodynamic cavitation in the BT device was less efficient. The differing results can be well explained by considering the bending-induced delamination of cellulose fibers as the leading process. The intra-fiber fibril cohesion is stronger for nonderivatized fiber pulp of DCe and ECe^81,82^ and larger mechanical forces, i.e., stronger bending, would be needed to delaminate the fibrils within the cellulose fiber. In BT compared to UH, an order of magnitude fewer number of cavities collapse, result in a weaker treatment and mechanical forcing. Bending delamination is successful when the tension between the fibrils overcomes the cohesion, which is more likely for the case for charged fibers and the larger number and intensity of cavitation events which we indicate in Figure 10c.

#### 3.4.3 Effect of the Sonication Intensity

**Figure 11:**
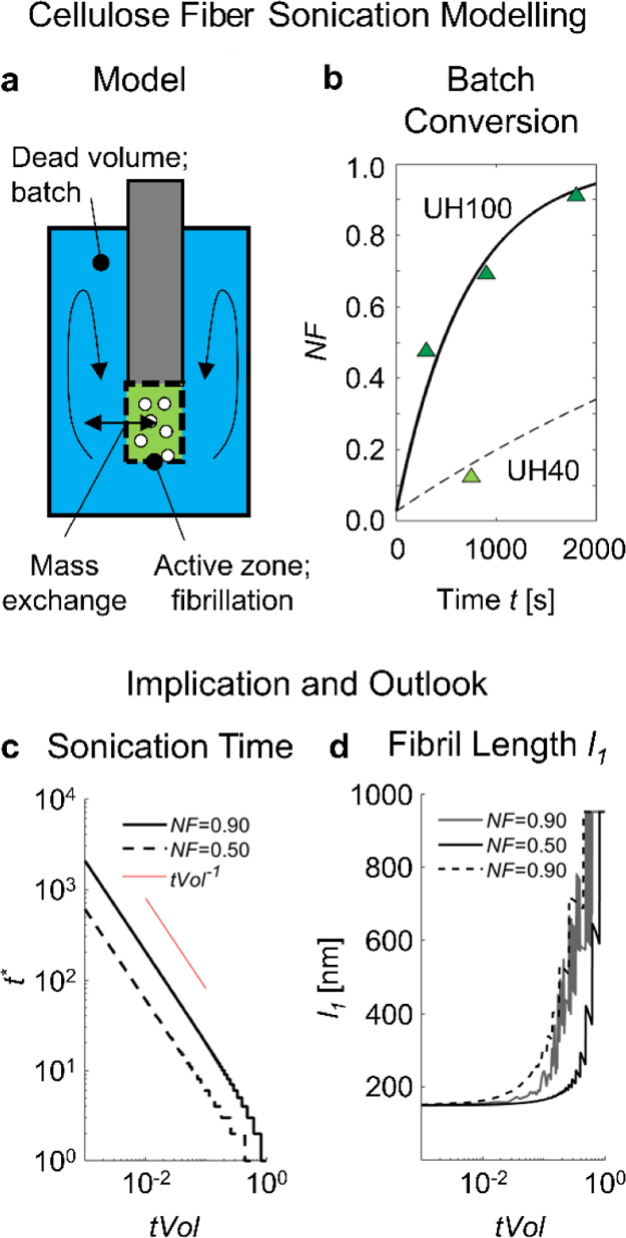
Cellulose sonication and scaling with the batch size. (a) Model of our fiber batch sonication with (b) comparing the model results to the experimental measurements. UH100 as a solid line and UH40 as dashed line. (c) Impact of the relative active volume *tVol* size on the normalized sonication time t* = *t*^.^*con* for two target nanocellulose fraction *NF*. (d) Associated development of the mean fiber length assuming a simple fiber shortening by breakage in half. Solid line: breakage at every cycle. Dashed line: breakage at every fourth cycle.

Pagani et al.^35^ documented that CNTs were affected by the cavity only within a certain distance, in their case, 250 μm. Statistically, the mean distance of fibers to the cavity decreases with an increasing cavity number and size, resulting from a higher sonotrode amplitude, given to operate below cavitation shielding.^83^ From that, we developed the model view (Figure 11a and more explained in the Supporting Information) of (i) fibrillation in an active zone below the sonotrode tip, i.e., the cavitation bubble volume identified in Figure 2, (ii) inert behavior elsewhere in the batch considered as dead zone, and (iii) free mixing of the suspension between the zones. The conversion of cellulose fibers to *NF* then scales with the number of successful cavitation fibrillation events per active volume area. The number of successful cavitation events was inaccessible to us. Instead, we tested our model assumptions by correlating results from UH100 to UH40 treatments. We first fitted a fiber-to-fibril conversion rate on to UH100, which resulted to 1.1 s^–1^. We then scaled this conversion rate to UH40 with the caloric power ratio, i.e., *P*_FS,0.4%_(UH40)/*P*_FS,0.4%_ (UH100) being 0.32 s^–1^. The model results for UH100 and UH40 compare well to the measurement results (Figure 11b).

From that agreement, we can deduce two hypotheses: first, we operated with UH40 to UH100 in a linear range with no significant regime change from an increase in amplitude. Second, the fibrillation time, sonication amplitude, and active zone ratio (batch size for the same cavitation settings) are directly correlated. That may be evident, but it appears to be often overlooked in the literature and can be summarized as follows: more fibrillation for a smaller batch size (i.e., sample volume), higher amplitude, and longer time. In Figure 11c, we show the dependence of the normalized cavitation time t* = *t*^.^*con*, which is the number of successful conversion cycles needed in the active zone, on the ratio of the active volume *tVol*. t* scales inversely with *tVol*, below *tVol* ∼ 0.1, which is ca. 1.3 mL for UH100. Hence, half the batch size results in half the conversion cycles to achieve the same degree of fibrillation, which is no gain for the throughput but for the preservation of the fibril length. As fibers and fibrils are mixed in the dispersion, a larger number of dispersion cycles does result in a larger number of fibril breakage events, especially when a complete conversion, i.e., a high nanofibril fraction *NF*, is aimed for. We qualitatively exemplify this in Figure 11d, assuming a shortening of fibrils into halves at every conversion cycle (solid) or every fourth (dashed). The oscillation at small *tVol* results from the arbitrary chosen homogenous breakage of the fibrils into two equally sized fibrils, which is a heuristic. The breakage type and breakage rate of CNFs are unknown and we encourage a dedicated follow-up study, for example, along the guiding work of CNT breakage by sonication.^35,36^ The qualitative impact of *tVol* is, however, clear. For producing short nanofibrils which are cellulose nanocrystal like^9^ by sonication, a large batch can be treated for a long time by cavitation exposure. For producing long fibrils by sonication, the batch size should be small or, in the limiting case, converging to a continuous treatment process where the flow rate is adjusted to the sonotrode active volume (Figure 2c). Alternatively, the cellulose suspension can be fibrillated to a lower *NF*, followed by a separation step of the fibrils from the remaining fibers and fragments, which are exclusively sonicated in the following step. That is also possible in one pot and is comparable to common particle comminution with (internal) recirculation of the larger particles. Surprisingly, Saito et al.^80^ employed such dedicated mechanical treatment to produce CNFs with length *l* > 2000 nm. The focus of their work was on the TEMPO oxidation of the cellulose fiber at neutral pH conditions and the effect of the processing conditions was not further discussed. Based on our findings, we argue that their chosen short fibrillation time with the separation and recycling of the remaining cellulose fibers and fragments was key to their success.

## 4 Conclusions

We studied the effect of cavitation on cellulose fibers with a focus on fibrillation into nanocellulose. We included three types of cellulose: DCe, ECe, and derivatized cellulose with an increased anionic charge of 600 μeq/g from TEMPO oxidation and carboxymethylation. We treated these fibers with hydrodynamic and acoustic cavitation. In our case, the intensive fibrillation of the cellulose fiber pulp into CNFs was achieved with sonication because more energy was introduced into the suspension. CMCe and TEMPO-oxidized cellulose were efficiently fibrillated to the elementary level, concluded from a linear increase in the mass-based nanosized fraction and the ratio of the surface accessible by polymeric counter ions from charge titration. The cellulose crystallinity decreased by the extend expected for fibrillation and also observed for the noncavitating treatment, i.e., microfluidization, concluding that cavitation had no significant negative impact on the cellulose crystallinity. Probed by ATR FTIR and XPS, we did not observe a significant change in the glucose-polymer chemistry.

The damage type observed using LOM and SEM resembled broken laminates, for example, a wood branch. From the observations, we conclude that microstreaming from the growth and collapse of cavities triggered the bending delamination of cellulose fibers, leading to their fibrillation. Ductile cellulose fibers and fibrils are probably rapidly bend toward the collapsing or outward of the growing cavity, which causes its full or partial breakage at the outer bend. Fibril bending as a dominating mechanism in fibrillation could also explain why sonication is efficient as microfluidzation or homogenization post-treatment; in sonication, a smaller bending curvature is achieved. Fibrillation by sonication in our study was, however, limited to the lower fiber pulp concentration, where it was successful at 0.4% mass-based concentration *C*_m_ but failed at 2% for CMCe. We speculate that sonication fibrillation is limited to the dilute regime, i.e., below the connectivity limit;^44^ CNF dispersion but not gel.

With a simple model derived from our understanding of microstreaming-based sonication fibrillation, we qualitatively demonstrate the importance of the batch size on the resulting fibril length for a given target *NF*. In large batches, short fibrils are to be expected. To produce long fibrils by sonication, we suggest a continuous sonication with an active zone adjusted flow rate and/or the separation of the long CNFs from the remaining fibers and fragments, which is possible for continuous and batch processing.

## Supporting Information Available

Estimation of tensile forces on the fiber in BT treatment; cavitation characterization (power and bubble collapse frequency); note on CNF Sedimentation; AFM fibril dimensions; fiber length distribution; details on the crystallization index analysis, ATR FTIR analysis and XPS analysis; estimation of the fiber ends development; modeling of the fiber batch sonication; LOM Images; and SEM Images (PDF)

## Notes

The following documents are referred to in the Supporting Information but not in the main document: Fan and Ahmadi,^84^ French and Santiago Cintrón,^85^ Nam et al.,^86^ French,^87^ Park et al.,^88^ Yao et al.,^89^ Wojdyr,^90^ Abidi et al.,^91^ Oh et al.,^92^ Lavoine,^93,94^ Maréchal and Chanzy,^95^ and Dassanayake et al.^96^
