# Supplementary material for: Cavitation Fibrillation of Cellulose Fiber
Source: Biomacromolecules. 2022 Jan 31;23(3):847–62. doi: 10.1021/acs.biomac.1c01309 (PMC8924874; doi:10.1021/acs.biomac.1c01309)
Supplement: Supplementary file 1 — bm1c01309_si_001.pdf [file bm1c01309_si_001.pdf]

Supporting Information

Cavitation Fibrillation of Cellulose Fibres

Jakob D. Redlinger-Pohn[1,2,], Martin Petkovšek[3,],

Korneliya Gordeyeva[1], Mojca Zupanc[3], Alisa Gordeeva[4], Qilun Zhang[5],

Matevž Dular[3], L. Daniel Söderberg[1,2]

Affiliations

[1]Department of Fibre and Polymer Technology, KTH Royal Institute of Technology, Sweden

[2]Treesearch, Sweden

[3]Faculty of Mechanical Engineering, University of Ljubljana, Slovenia

[4]Department of Materials and Environmental Chemistry, Stockholm University, Sweden

[5]Laboratory of Organic Electronics, Linköping University, Sweden

Supporting Information Content

The Supplement Information document consists of 52 pages, having 55 Figures S and 7 Table S. Figure S includes at the end of the document 21 Figure S consisting of 144 additional LOM images and 7 Figures S consisting of 117 additional SEM images.

Table of Content

1. Blow-Through Device Hydrodynamics 4

2. Cavitation Characterization 5

2.1. Caloric Power Input 5

2.2. Bubble Collapse Frequency 6

3. Material Analysis 8

3.1. Cellulose nanofibril (CNF) Separation by Centrifugation 8

3.2. AFM Measured Fibril Length and Diameter 10

3.3. Fibre Length Distribution Plots 11

3.4. Crystallinity Index *CI* of the Cellulose Samples 14

3.4.1. Motivation for two different Methods 14

3.4.2. Method Description 14

3.4.3. XRD Diffractogram and its Deconvolution 17

3.5. Attenuated Total Reflection Fourier Transform Infrared Spectroscopy (ATR FTIR) 22

3.5.1. ATR FTIR Spectra of the used cellulose fibre pulps 22

3.5.2. ATR FTIR Spectra after Ultrasonication UH 24

3.5.3. X-ray Photoelectron Spectroscopy (XPS) Spectra 25

3.5.4. Estimation of the Fibril Surface Polymer Degree of Substitution 25

3.5.5. Hydrolysis of the Glucose Polymer 26

3.5.6. Development of the Fibre Ends 26

4. Modelling of the Cellulose Fibre Fibrillation by Batch Sonication 29

5. Additional Images of The Fibre and Fibrils from Treatment..32

5.1. Light Optical Microscopy (LOM) Images 32

5.1.1. Dissolving cellulose fibre pulp (DCe) 32

5.1.2. Enzymatic Pre-Treated Cellulose (ECe)..34

5.1.3. Carboxymethylated Cellulose (CMCe)..36

5.1.4. TEMPO oxidized cellulose (TCe) 41

5.2. Scanning Electron Microscopy (SEM) Images 43

5.2.1. Carboxymethylated Cellulose (CMCe)..43

6. References in the Supplement Material 50

1. BLOW-THROUGH DEVICE HYDRODYNAMICS

The suspension in the blow-through device BT is accelerated in a converging nozzle. The upstream height H_{UP} = 15 mm and the height at the cavitation nozzle is H = 1 mm. The velocity translation is hence 15 and the convergence length L_{Nozzle} = 44 mm. The thickness is constant 5 mm. The nozzle geometry is presented in Figure 2 of the main document. The suspension mass is ca. 1500 g, and the volume approx. $1.5 \cdot 10^{-3}$ m³. The flow-through time is approx. 9 s. The mean upstream velocity U_{Up} and nozzle velocity U calculate hence to 2.22 m/s and 33.33 m/s, respectively. The acceleration a_{Nozzle} is calculated from the mean velocity:

$$a_{Nozzle} = \frac{\left(U - U_{Up}\right)}{t_{Nozzle}}, \text{ with} \quad \text{(SE1)}$$

$$t_{Nozzle} = \frac{L_{Nozzle}}{0.5\left(U + U_{Up}\right)}, \quad \text{(SE2)}$$

resulting to a_{Nozzle} = 1280 m/s². That is by orders of magnitude smaller than the acceleration into microchannels, recently reported by [1]. Following their argumentation, our acceleration and induced elongational stresses are too small for breaking the CMCe fibres (carboxymethylated fibres at a degree of substitution D.S. = 0.1) from exceeding the fibre tensile stress. The charge of the TCe fibres is comperable, and lower for the dissolving pulp fibres. We hence can expect that our fibres to not suffer fragmentation from elongational stress. However, deformations or structural damages can be induced.

2. Cavitation Characterization

2.1. Caloric Power Input

The caloric power was calculated from the adiabatic temperature increase with treatment time, respectively, energy input. The batch ultrasonication UH was performed in an insulated cup and the blow-through device BT was covered by insulating foam. The temperature was monitored with a resistance thermometer Pt100. The heat capacity of the fibre suspension (at a low mass concentration) was taken identical to water, taken as 4181 J/kg/K. Case details, and resulting caloric power of UH and BT are listed in Table S1 and Table S2 respectively.

Table S1: Caloric power of ultrasonication UH with the ColeParmer 750W sonotrode.

	UH40	UH40 0.4% CMCe	UH100	UH100 0.4% CMCe
ΔT [°C]	27.4	31.4	49.6	48.4
m [g]	499	449	499	499
E [kJ]	57.1	58.9	103.4	100.9
Δt [min]	30	30	15	15
P [W]	32	33	115	112

Table S2: Caloric power of hydrodynamic cavitation treatment in the blow-through device BT.

	BT 5 bar	BT 5 bar 0.4% CMCe	BT 5 bar 2% CMCe	BT 7 bar	BT 7 bar 0.4% CMCe
ΔT [°C]	11.4	10.6	9.3	18.2	16.7
m [g]	997	997	997	997	997
E [kJ]	47.5	44.2	53.8	75.9	69.6
Δt [min]	60	60	50	60	60
P [W]	13	12	13	21	19

2.2. Bubble Collapse Frequency

Bubble collapse frequency for the hydrodynamic cavitation in the blow-through device (BT) and acoustic cavitation with the sonotrode (UH).

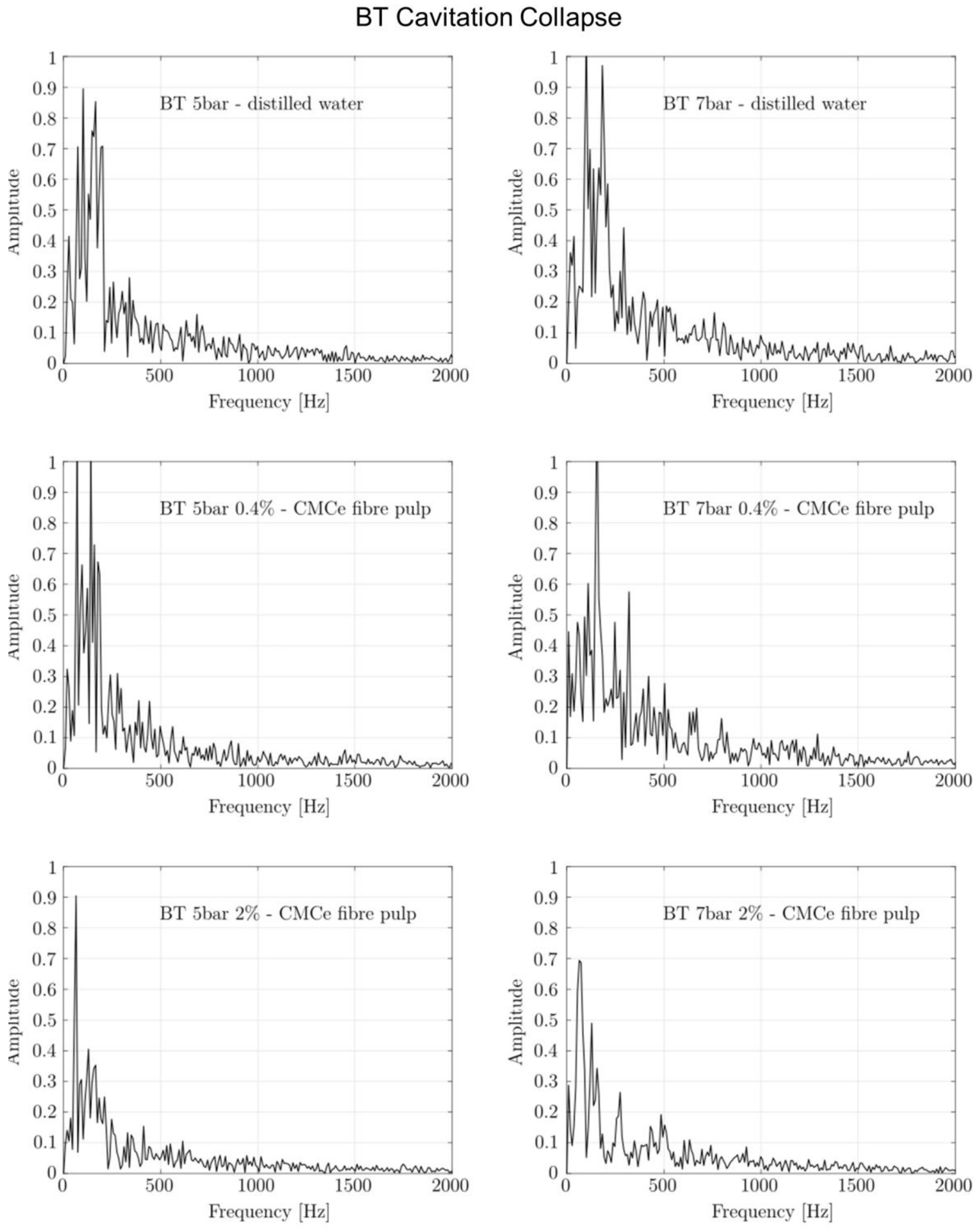

Figure S1: Bubble collapse frequency of BT for water, 0.2% CMCe fibre pulp concentration and 4% CMCe fibre pulp concentration.

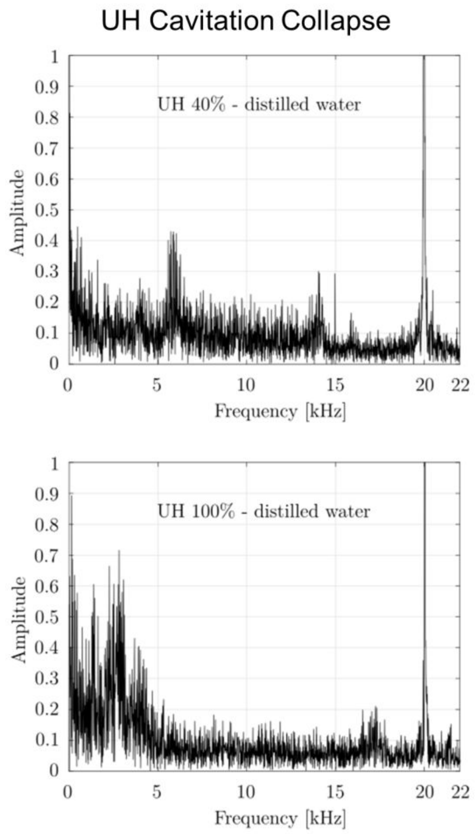

Figure S2: Bubble collapse frequency of UH for water at 40% amplitude/power (UH40) and 100% amplitude/power (UH100).

3. Material Analysis

3.1. Cellulose nanofibril (CNF) Separation by Centrifugation

The nanocellulose content *NF* and the morphology characterization by atomic force microscopy (AFM) include both a centrifugation preparation step. *NF* utilizes the size-exclusion from sedimentation in the centrifugal field as characterization means. *NF* is hence no absolute value but dependent on the centrifugation settings; sampling height (volume and tube cross-section), time and acceleration. For AFM, centrifugation is added as preparation step to exclude larger particles which could damage the AFM cantilever or deteriorate the measurement. For the interpretation of *NF* and AFM measurement we attain estimations of the accept fibril morphology by modeling is as an individual elongated particles settling in an undisturbed fluid, as it is done as first principal estimation in particle sedimentation.

The cut-off fibril size can be estimated from the terminal settling velocity $U_{sed,a}$, calculated from the force balance on the fibril:

$$0 = F_C - F_B - F_H = (\rho_F - \rho_L) V_F a_z - \eta\pi \frac{l}{2AR} c_d U_{sed,a}\,, \qquad \text{(SE3)}$$

F_C, F_B and F_H being the centrifugal, buoyancy and hydrodynamic drag force, respectively. ρ_F and ρ_L are the fibril and fluid/water density, respectively, taken as 1500 kg/m³ and 1000 kg/m³. V_F is the fibril volume, assuming cylindrical shape. η is the dynamic viscosity taken as 0.001 Pas. $U_{sed,a}$ is the terminal settling/sedimentation velocity for which we solve and c_d is the fibril drag coefficient, which scales with its aspect ratio *AR* (i.e. the ratio of the fibre length *l* to the fibre diameter *d*) and depends on the fibril orientation with respect to the direction of acceleration (for a detail explanation see [2]). The drag force coefficient of a fibril having cylindrical shape, orientated parallel ($c_{d,p}$) and normal ($c_{d,n}$) to the direction of acceleration is:

$$c_{d,p} = \frac{8\left(AR^2-1\right)}{\left[\left(2AR^2-1\right)\ln\left(AR+\left(AR^2-1\right)^{0.5}\right)\Big/\left(AR^2-1\right)\right]-AR}\,, \qquad \text{(SE4)}$$

$$c_{d,n} = \frac{16\left(AR^2 - 1\right)}{\left[\left(2AR^2 - 3\right)\ln\left(AR + \left(AR^2 - 1\right)^{0.5}\right) \Big/ \left(AR^2 - 1\right)\right] - AR}. \tag{SE5}$$

The centrifugation seetings are summarized in Table S3 and the modelled fibril-cut of size, estimated by comparing $U_{sed,a} \cdot t$ to hs, is presented in Figure S3a for *NF* and Figure S3b for AFM. Estimations are, that *NF* includes fibrls up to a diameter of 150 µm to 250 µm. In AFM, fibrils with a thickness up to ca. 20 nm will be measured.

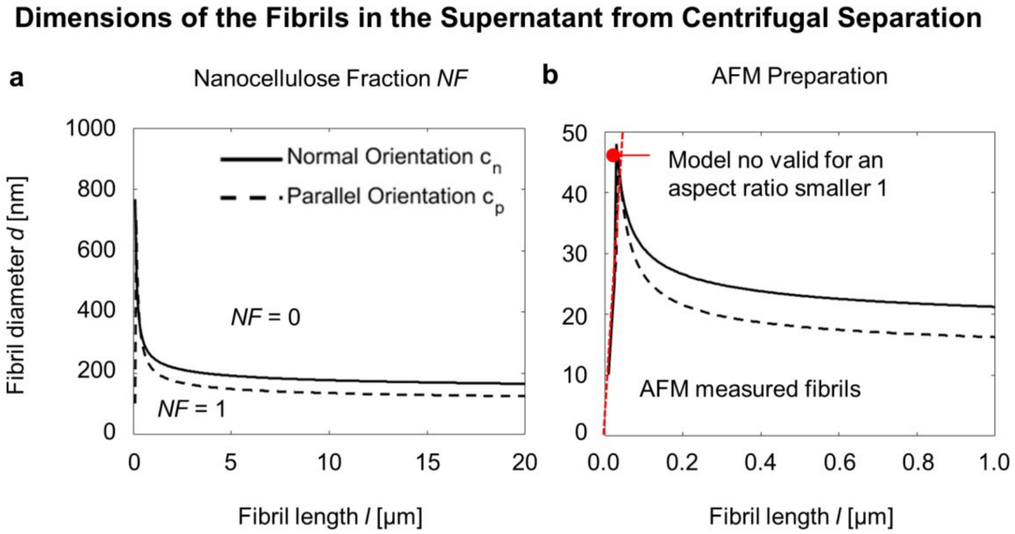

Figure S3: Fibril dimension in the accept from sedimentation separation by centrifugation.

Table S3: Centrifugation setting for *NF* and AFM sample preparation.

	Acceleration a_z [m/s²]	Time t [s]	Sampling height h_s [mm]	Concentration C [%]
NF	9,810	900	46	0.02%
AFM	19,620	3,600	5	0.01%

3.2. AFM Measured Fibril Length and Diameter

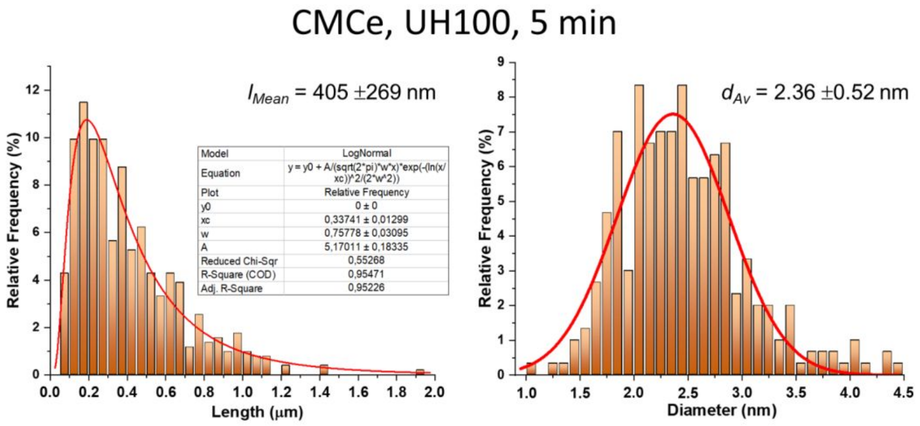

Figure S4: CMCe fibril dimension after UH100 for 5 min. Left: mean length l_{Mean} = 405±269 nm. Right: average diameter d_{Av} = 2.36±0.52 nm.

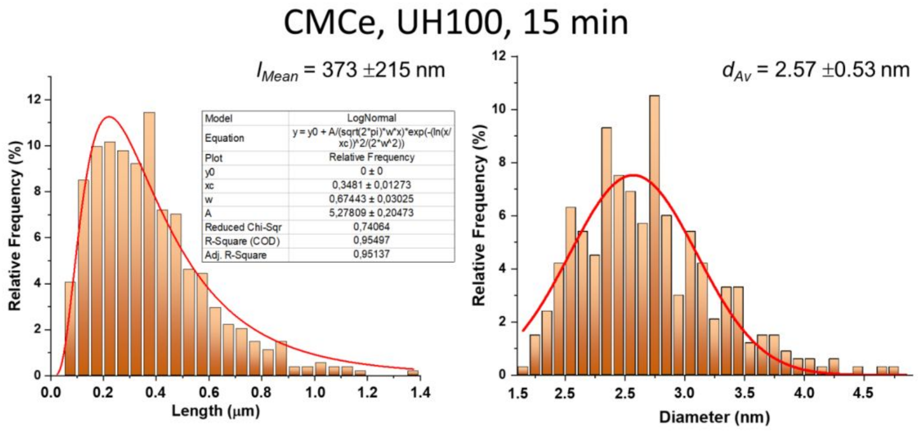

Figure S5: CMCe fibril dimension after UH100 for 15 min. Left: mean length l_{Mean} = 373±215 nm. Right: average diameter d_{Av} = 2.57±0.53 nm.

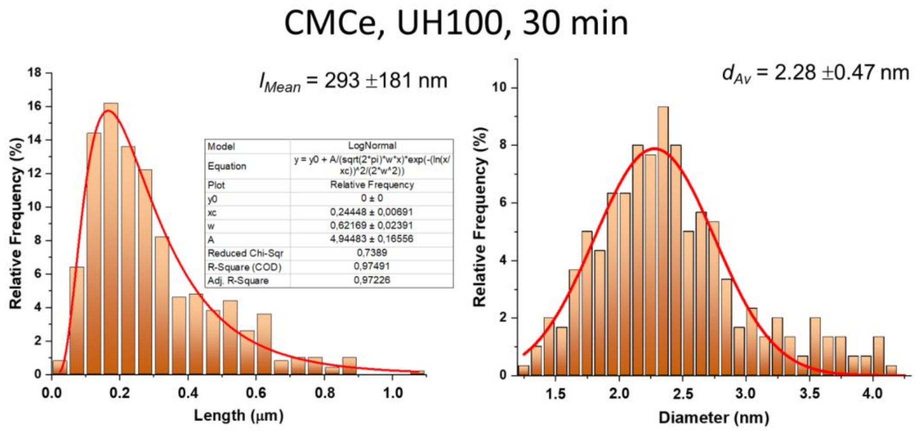

Figure S6: CMCe fibril dimension after UH100 for 30 min. Left: mean length l_{Mean} = 293±181 nm. Right: average diameter d_{Av} = 2.28±0.47 nm.

3.3. Fibre Length Distribution Plots

Fibre length distribution of the dissolving cellulose fibre DCe, enzymaticall treated cellulose fibre DCe, carboxymethylated cellulose fibre CMCe and TEMPO oxidized cellulose fibre TCe befor and after treatment by ultrasonication UH. The treatment intensity is stated in % of the maximum amplitude and treatment time. An additional case of UH40 and 4 min with negligible treatment is shown, which is not presented in the main document. The standard deviation is calculated from a duplicate measurement. The distribution is based on the fibre volume, i.e., ΔQ_3, calculated with the fibre length and diameter measured data assuming a cylindrical shape for what the volume-based distribution is identical with a length-based distribution, i.e., ΔQ_1.

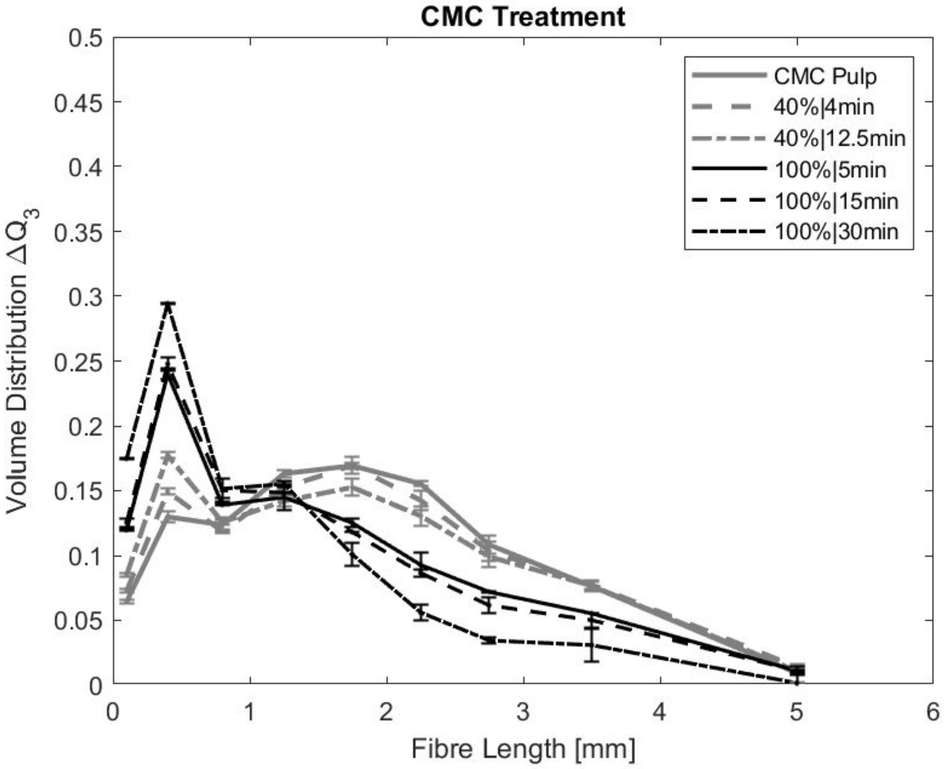

Figure S7: Volume-based fibre length distribution ΔQ_3 of CMCe for various ultrasound treatment intensities.

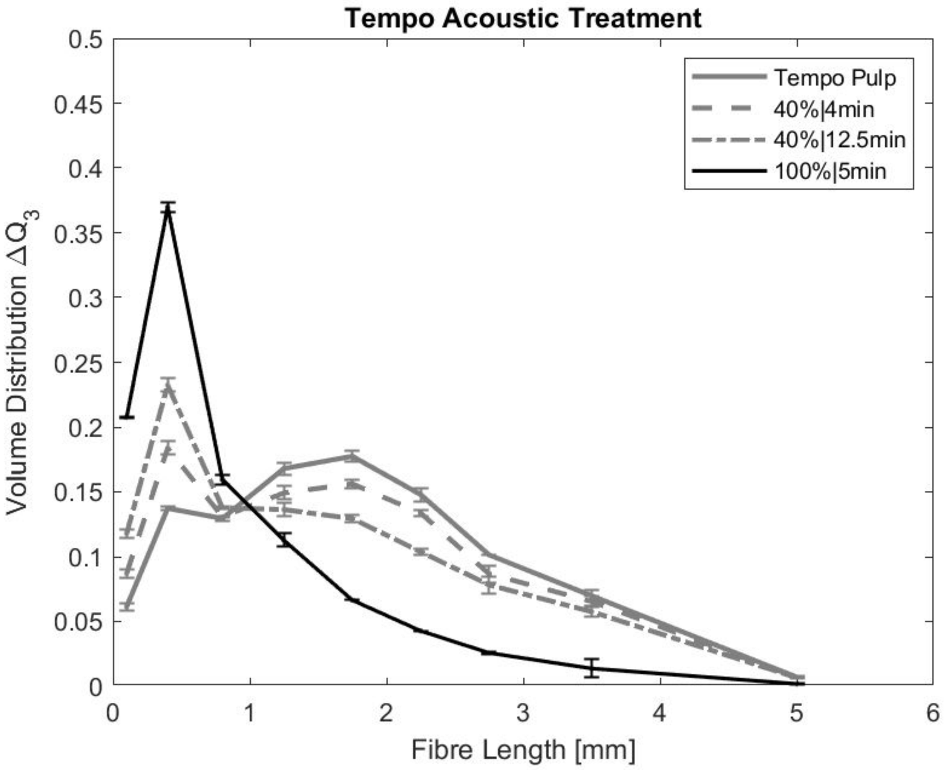

Figure S8: Volume-based fibre length distribution ΔQ_3 of TCe for various ultrasound treatment intensities.

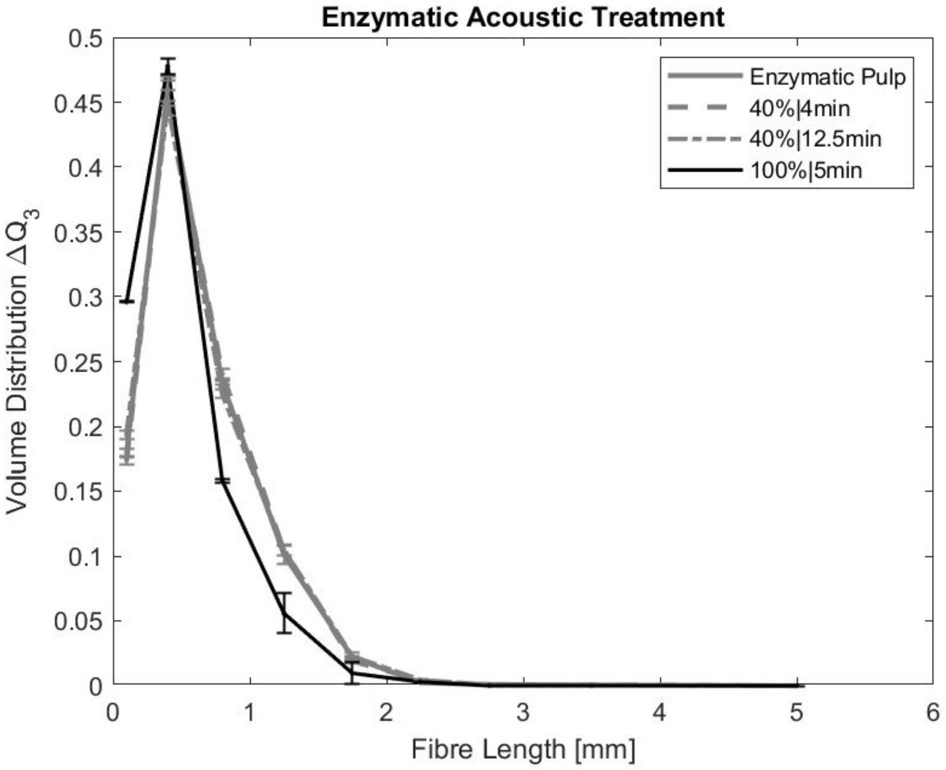

Figure S9: Volume-based fibre length distribution ΔQ_3 of ECe for various ultrasound treatment intensities

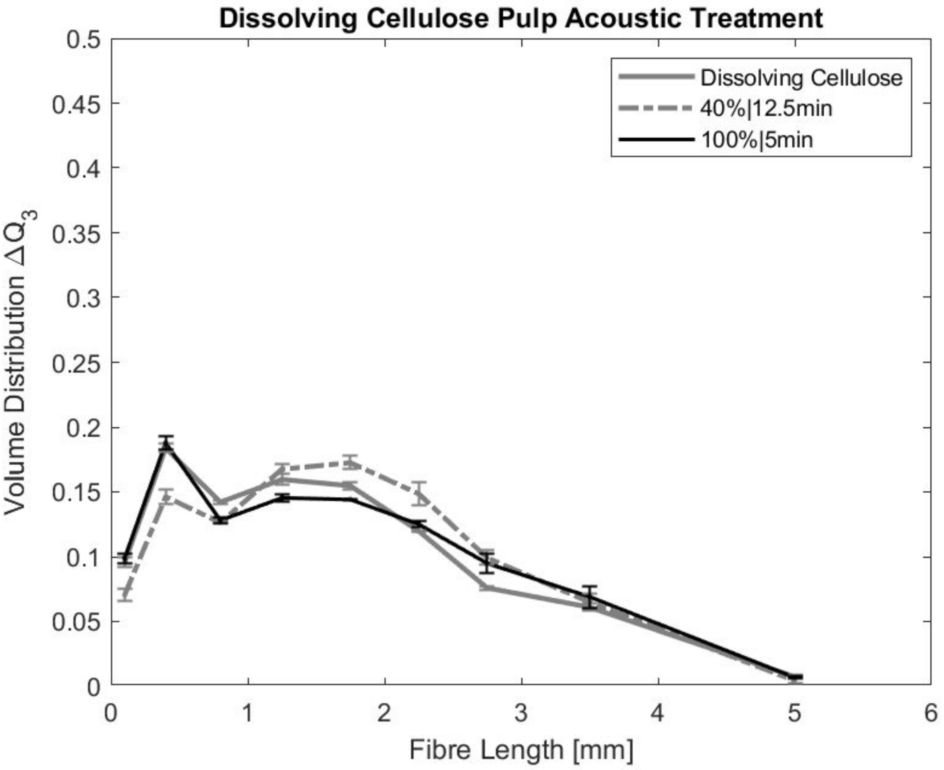

Figure S10: Volume-based fibre length distribution ΔQ_3 of DCe for various ultrasound treatment intensities

3.4. Crystallinity Index *CI* of the Cellulose Samples

3.4.1. Motivation for two different Methods

Cellulose crystallinity index (*CI*) is often calculated from 1D X-ray diffractograms using Segal Method [3]. However, in recent years the accuracy of the method was doubted [4–6], which has motivated the use of other methods, such as those based on deconvolution [7–9].

3.4.2. Method Description

X-ray diffraction (XRD) patterns were collected on a Panalytical X′Pert PRO diffractometer operated with Cu Kα radiation and in Bragg-Brentano diffraction geometry. Pressed foam samples with a mean thickness under 1mm were mounted on a Si wafer zero-background holder spinning at a constant rate of 30rpm. Diffraction patterns were measured in a 2θ range 5 – 50° with 0.0167° step size.

The *CI* was calculated by deconvolution of 1D X-ray diffractograms using Cerro et al.'s [8] amorphous model and pseudoVoight functions to fit the main crystalline peaks ((1-10), (110), (012), (200) and (004) of cellulose-Iβ (PDF 00-056-1718)). The amorphous model was defined as a linear combination of nine Gaussian functions (LCOF) which, during the fitting, were represented by single variable – height, corresponding to a specific total area of the LCOF [8]. Fitting was performed using FitYK software [10] with fitting workflow and variables as described in Table S4 [8]. The *CI* were calculated with eqn. (SE6), as the % ratio of the area of the crystalline signals to the total area:

$$CI_{decon} = 100\left(\frac{A_{crystalline}}{A_{total}}\right), \tag{SE6}$$

Additionally the *CI* were calculated using Segal's method [3] which is based on measuring the intensity of the highest peak (I002, crystalline peak (002) of cellulose-Iβ (PDF 00-056-1718)) and the local minimum in the 2θ range of 17-19° (I_{min}) as in eqn. (SE7):

$$CI_{Segal} = 100\left(\frac{I_{002} - I_{\min}}{I_{002}}\right). \tag{SE7}$$

All XRD diffractograms and the deconvolution are presented following in Figure S12 to Figure S21. *CI* are listed in Table S4 and presented in Figure S11 over *RSC*, i.e,, the suspension surface charge normalized by the total charge including two literature references: R1 from Daicho et al. [11] who fibrillated TEMPO oxidiced cellulose fibre by microlfuidization (no cavitation method) and determined CI with a deconvolution method. R2 from Zhou et al. [12] who fibrillated TEMPO oxidized cellulose fibre by ultrasonication (UH) and determined CI using Segal's method [3]. The trend of CI_{Segal} and CI_{decon} are comperable, where the values are in general higher for CI_{decon}, which we present in the main document in Figure 8.

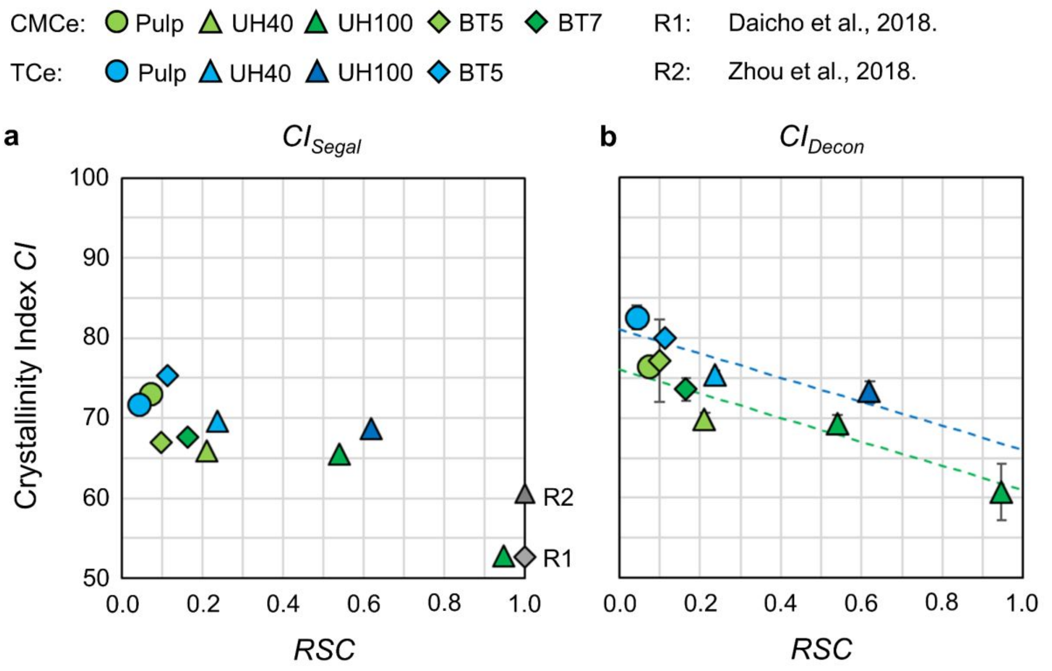

Figure S11: Crystallinity and chemistry development of the cellulose form cavitation treatment. a and b: crystallinity index CI_{Segal} and CI_{decon}, respectively, presented over *RSC*. R1 and R2 are reference value from Daicho et al. [11] and Zhou et al. [12], respectively for their cellulose fibrils.

Both indices, CI_{Segal} and CI_{decon} show a nearly linear decay with *RSC* (Figure S11). In section 3.2.2. of the main document we argue that the linear trend of *RSC* with *NF* results from an efficient fibrillation of cellulose fibres into the cellulose nanofibrils (CNF) at an elementary scale with all charges exposed. *CI* measured is a suspension value and hence mixture average of the cellulose fibre and CNF in the dispersion. The linear decay of CI is hence simply explained by a mixture of *CI* contribution from the cellulose fibre and *CI* contribution of the CNF. We fitted the decay for CI_{decon} (Figure S11b) and found a slope of -15 fitting TCe and CMCe, resulting to boundary values for TCe of $CI_{decon,TCe}(RSC{=}0) = 81$, $CI_{decon,TCe}(RSC{=}1) = 66$ and for CMCe of $CI_{decon,CMCe}(RSC{=}0) = 76$, $CI_{decon,CMCe}(RSC{=}0) = 61$.

Table S4: *CI* after Segal et al. [3] (CI_{Segal}) and the deconvolution method of Cerro et al. [8] (CI_{decon}) with the standard deviation and residuals of the fit to the data R^2_{decon}. The case description points to the XRD defractogram, Figure S12 to Figure S21. The symbols are used in the main document to identify the data point.

Pulp	Treatment	Symbol	CI_{Segal} [%]	CI_{decon} [%]	R^2_{decon}
CMCe	Pulp		69.8	76.3±0.3	0.9990
CMCe	BT5		66.5	77.1±5.1	0.9812
CMCe	BT7		65.7	73.5±1.4	0.9990
CMCe	UH40		68.8	69.8±0.8	0.9992
CMCe	UH100-5min		65.1	69.2±1.1	0.9992
CMCe	UH100-30min		52.0	60.7±3.5	0.9952
TCe	Pulp		74.0	82.5±1.5	0.9992
TCe	BT5		72.4	79.9±0.7	0.9993
TCe	UH40		72.1	75.3±0.6	0.9992
TCe	UH100-5min		67.6	73.2±1.3	0.9994

3.4.3. XRD Diffractogram and its Deconvolution

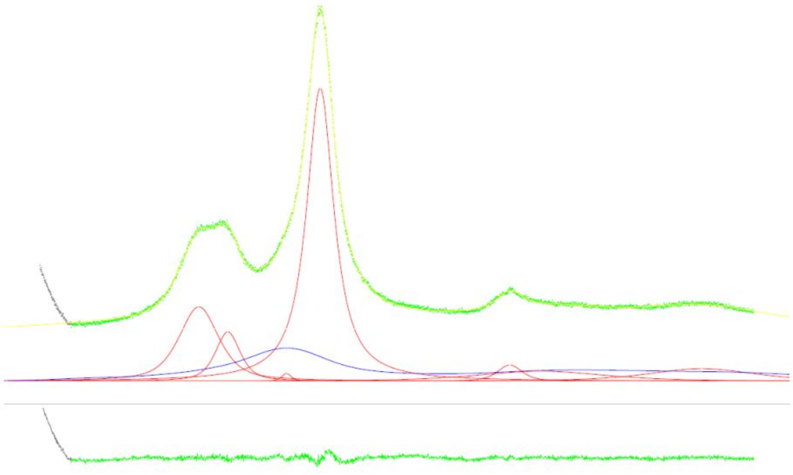

Figure S12: Deconvolution of XRD diffractograms for **CMCe Pulp** in Fityk. Data & functions: raw data (green), fitted data (yellow), amorphous function (blue), crystalline functions (red) and residual baseline error (green at the bottom of the figure).

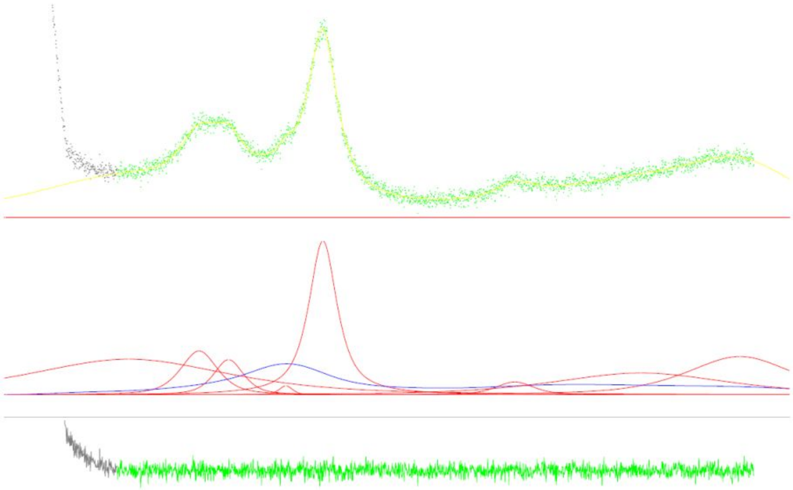

Figure S13: Deconvolution of XRD diffractograms for the **CMCe BT5** in Fityk. Data & functions: raw data (green), fitted data (yellow), amorphous function (blue), crystalline functions (red) and residual baseline error (green at the bottom of the figure).

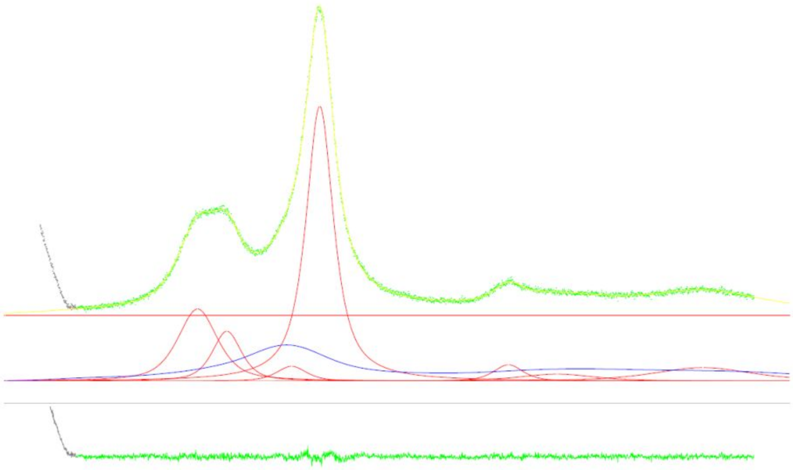

Figure S14: Deconvolution of XRD diffractograms for the **CMCe BT7** in Fityk. Data & functions: raw data (green), fitted data (yellow), amorphous function (blue), crystalline functions (red) and residual baseline error (green at the bottom of the figure).

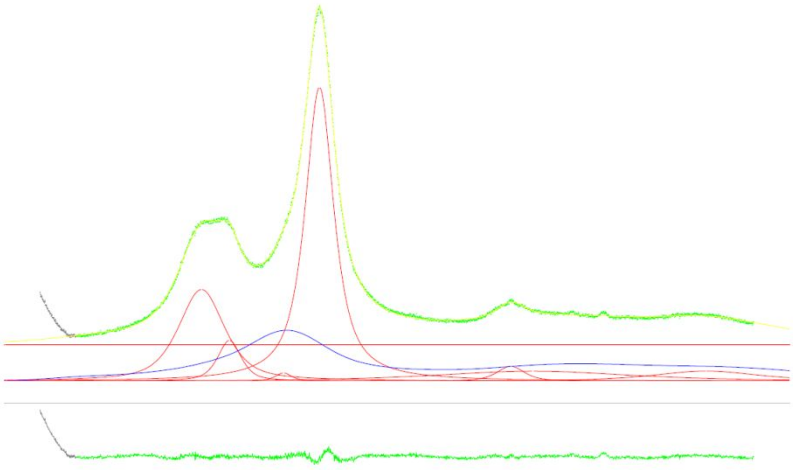

Figure S15: Deconvolution of XRD diffractograms for the **CMCe UH40** in Fityk. Data & functions: raw data (green), fitted data (yellow), amorphous function (blue), crystalline functions (red) and residual baseline error (green at the bottom of the figure).

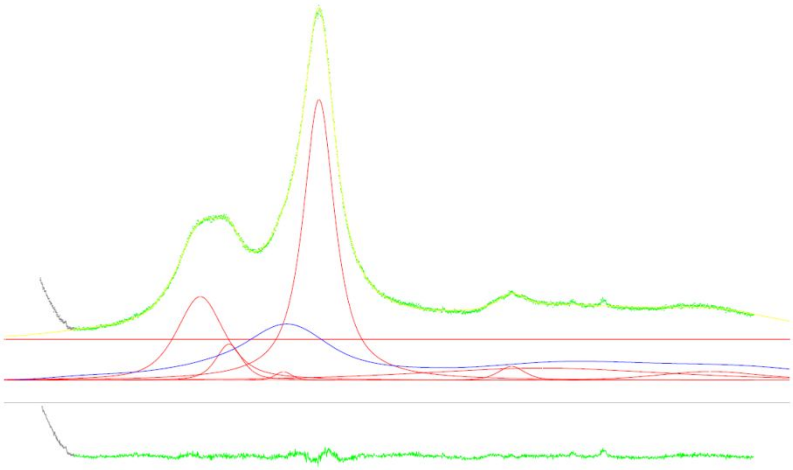

Figure S16: Deconvolution of XRD diffractograms for the **CMCe UH100-5min** in Fityk. Data & functions: raw data (green), fitted data (yellow), amorphous function (blue), crystalline functions (red) and residual baseline error (green at the bottom of the figure).

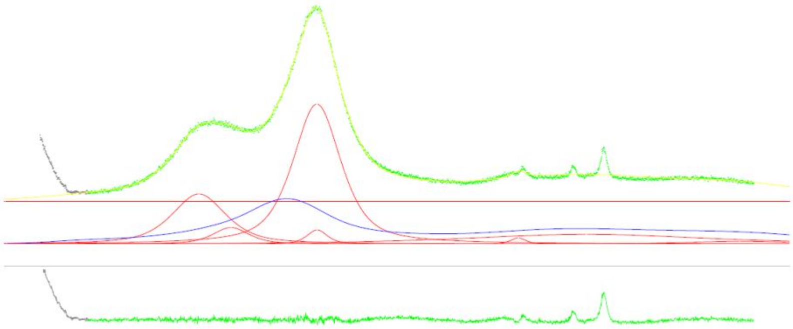

Figure S17: Deconvolution of XRD diffractograms for the **CMCe UH100-30min** in Fityk. Data & functions: raw data (green), fitted data (yellow), amorphous function (blue), crystalline functions (red) and residual baseline error (green at the bottom of the figure).

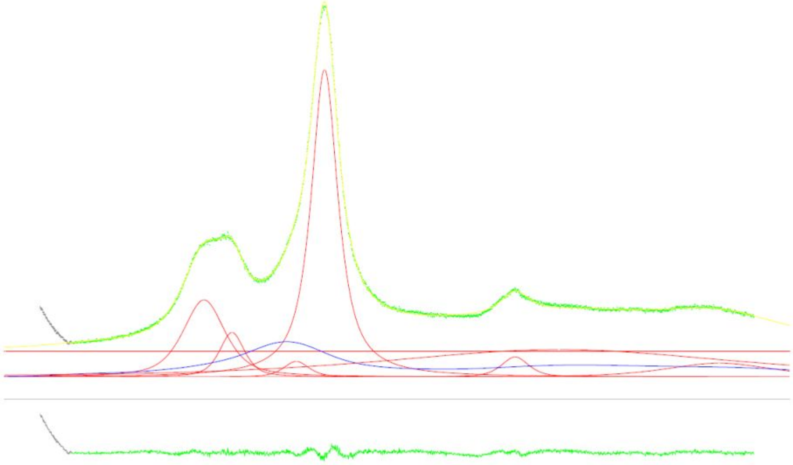

Figure S18: Deconvolution of XRD diffractograms for the **TCe Pulp** in Fityk. Data & functions: raw data (green), fitted data (yellow), amorphous function (blue), crystalline functions (red) and residual baseline error (green at the bottom of the figure).

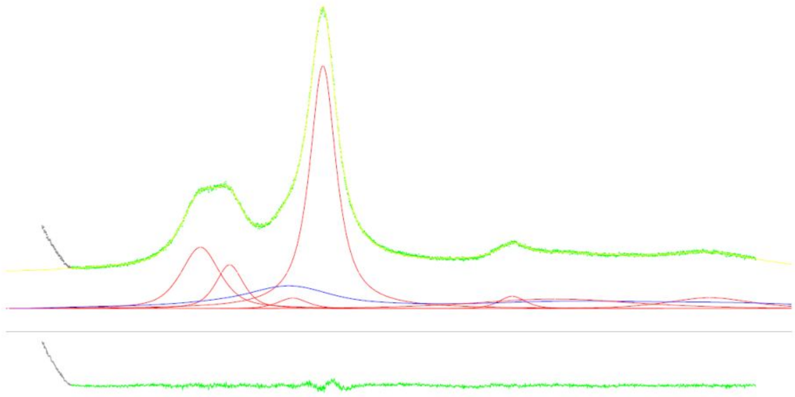

Figure S19: Deconvolution of XRD diffractograms for the **TCe BT5** in Fityk. Data & functions: raw data (green), fitted data (yellow), amorphous function (blue), crystalline functions (red) and residual baseline error (green at the bottom of the figure).

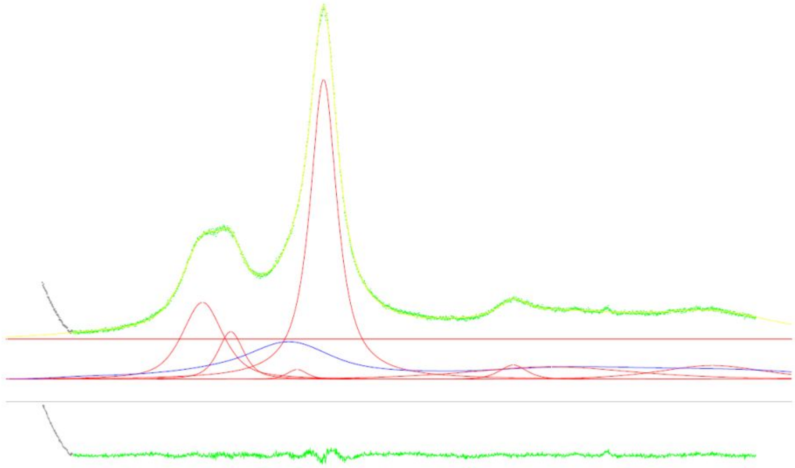

Figure S20: Deconvolution of XRD diffractograms for the **TCe UH40** in Fityk. Data & functions: raw data (green), fitted data (yellow), amorphous function (blue), crystalline functions (red) and residual baseline error (green at the bottom of the figure).

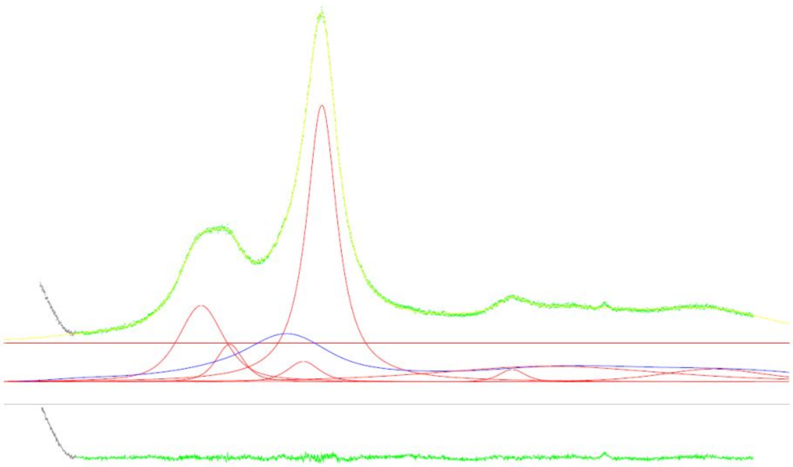

Figure S21: Deconvolution of XRD diffractograms for the **TCe UH100-5min** in Fityk. Data & functions: raw data (green), fitted data (yellow), amorphous function (blue), crystalline functions (red) and residual baseline error (green at the bottom of the figure).

3.5. Attenuated Total Reflection Fourier Transform Infrared Spectroscopy (ATR FTIR)

3.5.1. ATR FTIR Spectra of the used cellulose fibre pulps

In Figure S22, we show the ATR FTIR spectra of the cellulose fibre pulp used in this study, dissolving pulp DCe (from Domsjö), enzymatically treated cellulose ECe, carboxymethylated cellulose CMCe and TEMPO-oxidized cellulose TCe. DCe was the basis of CMCe and TCe. The increase of the -$COO^{-}Na^{+}$ relative intensity documents their successful modification. In Table S5 we document the individual chemical groups wavenumber in ATR FTIR taken from literature. In Figure S23 we present the ATR FTIR spectra after UH treatment (at 40% UH40 and 100% UH100) for DCe (a), ECe (b) and TCe (c). CMCe is presented in the main document Figure 9a.

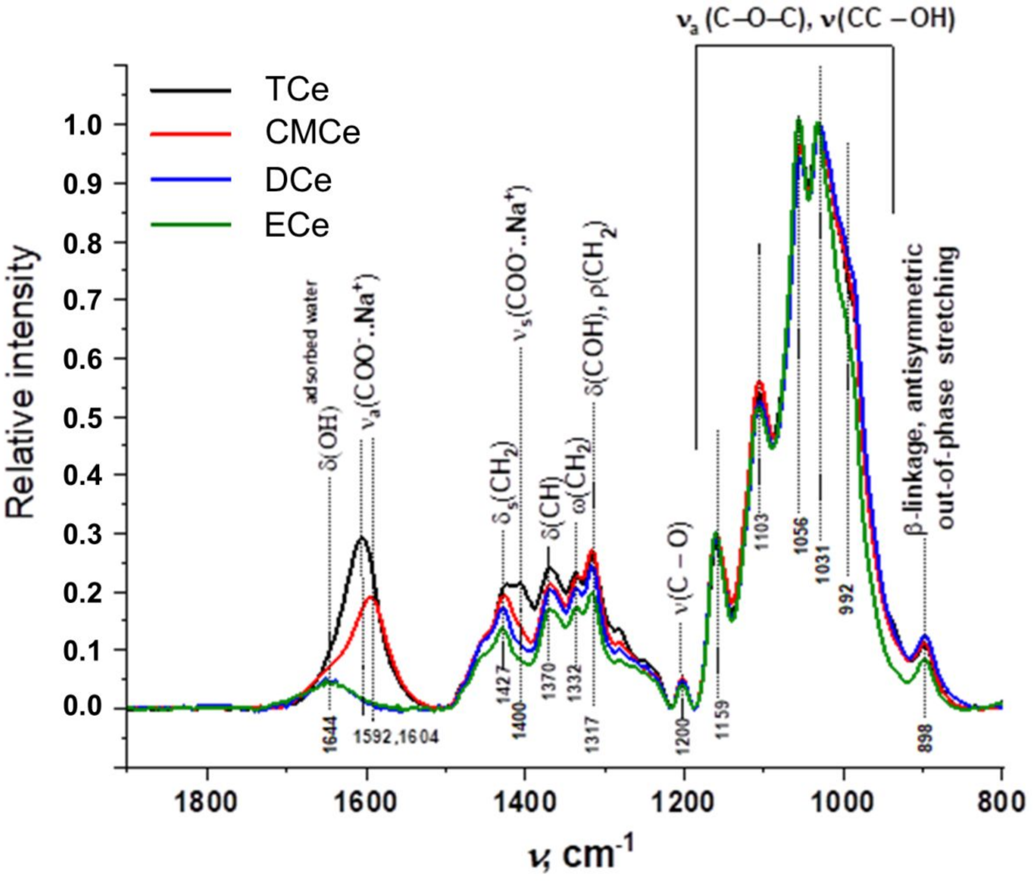

Figure S22: ATR FTIR spectra of Domsjö pulp (DCe, blue curve), Domsjö pulp after TEMPO mediated oxidation (TCe, black curve), Domsjö pulp after carboxymethylated oxidation (CMCe, red curve) and enzymatically treated pulp (ECe, green curve). All peaks are normalized on a band at 1030 cm^{-1}. y-axis labelling used comma instead of dots for decimals.

Table S5: Assignment of main vibrations in ATR FTIR spectra for different pulps.

Wavenumber, cm^{-1}	Assignment	Reference
1644	OH bending of adsorbed water	Abidi et al. [13], Oh et al. [14], Lichtenstein et al. [15]
1592 - 1604	COONa antisymmetric stretch	Lavoine et al. [16], Fujisawa et al. [17]
1427	CH_2 scissoring	Abidi et al. [13], Oh et al. [14]
1400	COONa symmetric stretch	Fujisawa et al. [17]
1370	CH bending	Abidi et al. [13], Oh et al. [14]
1332	CH_2 wagging, CO–H in-plane bending (?)	Abidi et al. [13], Maréchal et al. [18]
1317	CH_2 rocking, CO–H in-plane bending (?)	Abidi et al. [13], Maréchal et al. [18]
1200	C–O stretch, C–O–C symmetric stretch (?)	Abidi et al. [13], Maréchal et al. [18]
1159	C–O–C antisymmetric stretch	Abidi et al. [13], Lichtenstein et al. [15], Maréchal et al. [18], Dassanayake et al. [19]
1103	C–O–C antisymmetric in-plane stretch	Abidi et al. [13], Lichtenstein et al. [15], Dassanayake et al. [19]
1056	CC–OH stretching mode	Abidi et al. [13], Maréchal et al. [18], Dassanayake et al. [19]
1031	CC–OH stretching mode	Abidi et al. [13], Lichtenstein et al. [15], Maréchal et al. [18], Dassanayake et al. [19]
992	C–O and ring stretching modes	Abidi et al. [13], Maréchal et al. [18], Dassanayake et al. [19]
898	beta-linkages	Abidi et al. [13], Dassanayake et al. [19]

3.5.2. ATR FTIR Spectra after Ultrasonication UH

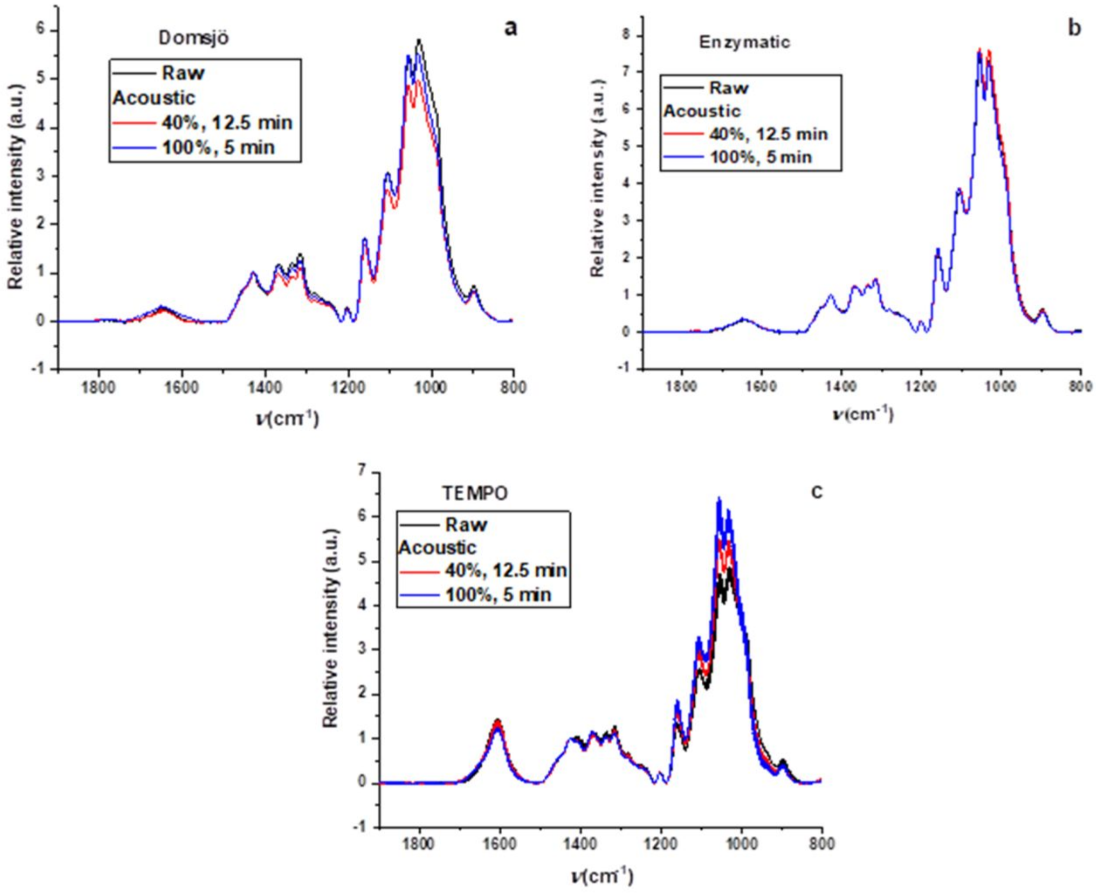

Figure S23: ATR FTIR spectra before and after ultrasonication treatment UH at 40% (UH40) and 100% (UH100) relative oscillation amplitude and different treatment time. a: DCe. b: ECe. c: TCe.

3.5.3. X-ray Photoelectron Spectroscopy (XPS) Spectra

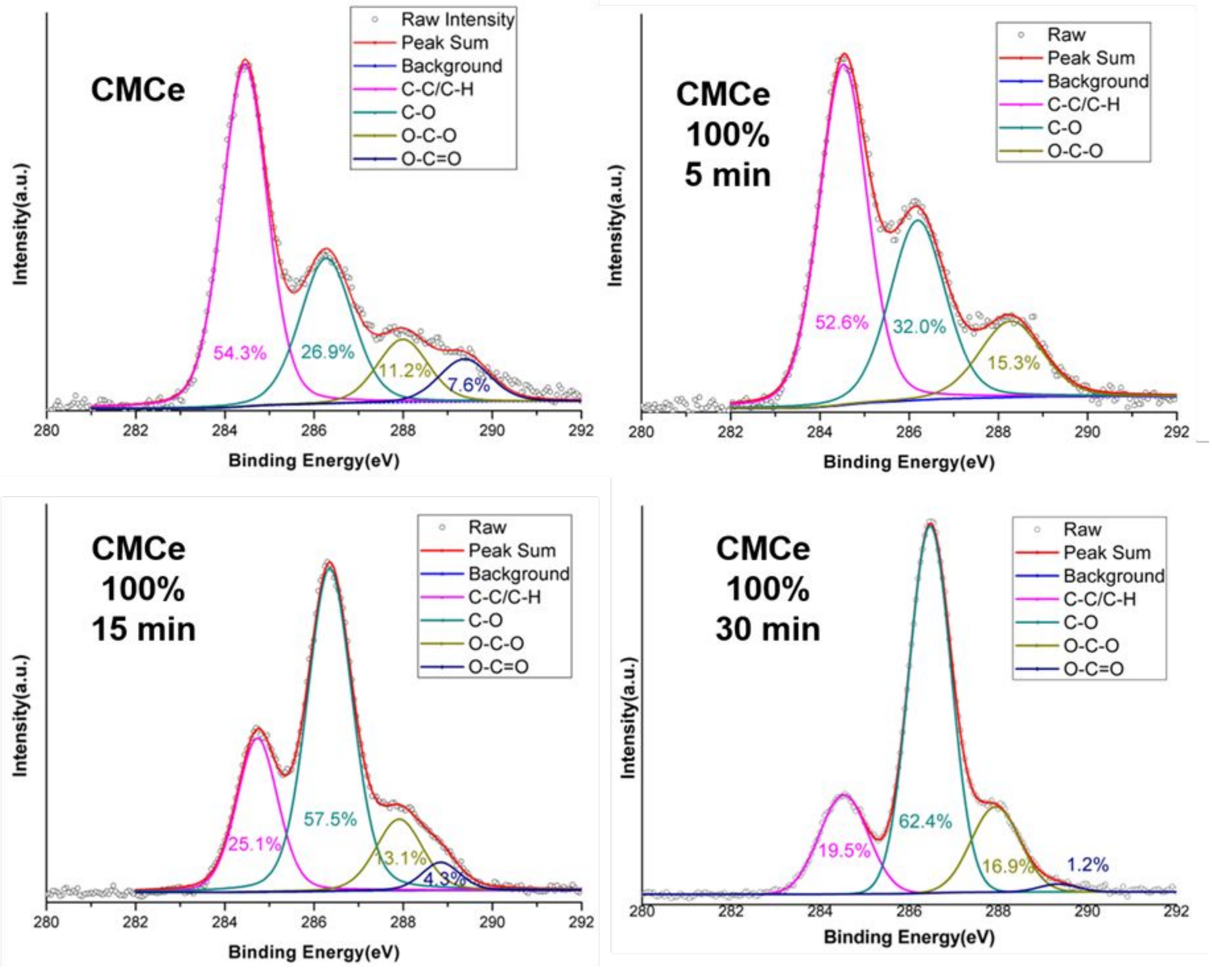

Figure S24: a XPS C(1s) spectra of CMCe untreated pulp (top left) and CMCe pulp treated by acoustic cavitation at 100% of amplitude after 5 min (top right), 15 min (bottom left) and 30 min (bottom right).

3.5.4. Estimation of the Fibril Surface Polymer Degree of Substitution

Hydroxyl groups of the glucose polymer accessible to the reagents are substituted during oxidation for what the carboxyl groups are to be expected localized at the fibril surface. The degree of substitution is however typically expressed as average over the total polymer chains of the fibril. In section 3.2.2. of the main document we show, that the accessible fibril surface charge is comparable to the total charge and conclude a fibrillation to the elementary scale what is backed by the AFM measurements shown in the main document section 3.2.3. The total charge of our CMCe was measured to 561 µeq/g what equals an average degree of substitution of 0.094. The actual degree of substitution assuming a preferred derivatization of the surface polymer chains can be estimated knowing (assuming) a ratio of the polymer chains at the surface to the total number of polymer chains per elementary fibril. The structure of the

elementary fibril is debated. Rosen et al. [20] discusses in a recent publication different shape of the elementary fibrils, all of them constituted of 18 polymer chains. In most of them, including the model which they identify as most probable, 14 polymer chains are at the surface of the fibril and 4 polymer chains are inside the fibril. In a considered model, 12 polymer chains are at the surface of the fibril and 6 polymer chains are inside the fibril. The corresponding degree of substitution for a preferred derivatization of the fibril surface polymer chain calculated by a linear extrapolation to 0.12 and 0.14, respectively.

3.5.5. *Hydrolysis of the Glucose Polymer*

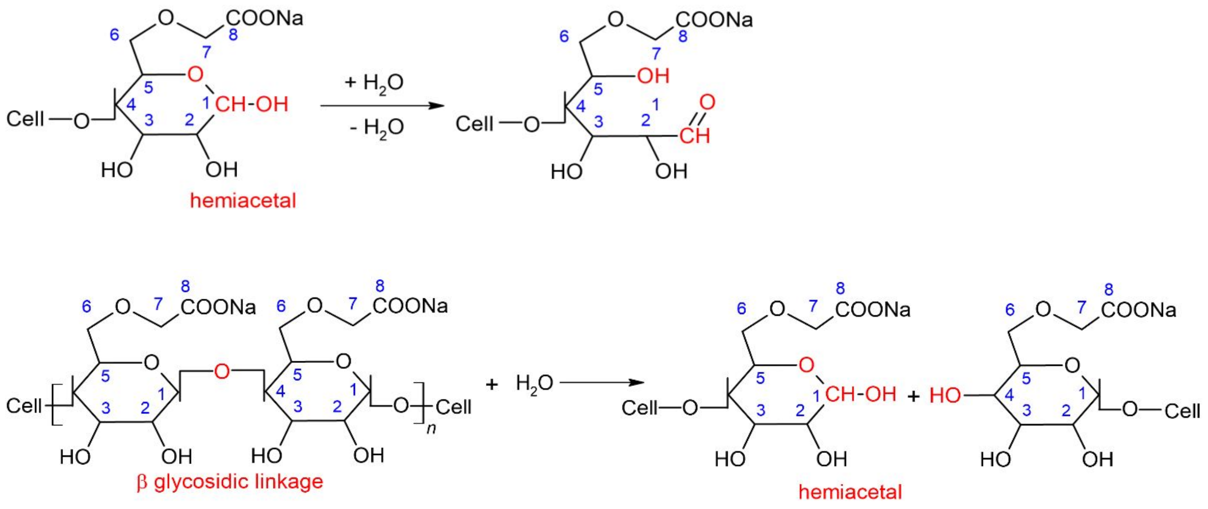

Figure S25: Chemical equation demonstrating. First row: hydrolysis of cellulose chain end. Second row: hydrolysis of glycosidic linkages in CMCe cellulose chains.

3.5.6. *Development of the Fibre Ends*

Our conclusion from the XPS measurement is an increase of ration C-O to the sum of O-C-O and O-C=O, consistent with the findings of Zhou et al. [21]. This increase levels off with the treatment time what we find consistent with reaching a limiting cellulose nanofibril (CNF) length shown in Figure 7d of the main document. To strengthen our argument we calculate the number of fibre ends per hypothetical cumulative of 1 mm as well as the relative number of fibre ends normalized with the value for pulp fibres. The elementary fibril in the CMCe fibre, $l_{Fibril,CMCe}$ can be in the order of several µm and could not be determined in this paper. Instead we take the $l_{1,95}$ value after CMCe UH100 5 min of ~ 1100 nm as hypothetical value for the following comparison in Table S6. The true value is likely higher and the estimated number of fibre ends for the pulp fibre would hence be lower. The number of fibre ends are calculated with the length-based distribution determined from the AFM images, shown in Figure 7d of the

main document. The calculation is sketched on hand of a simple case. Based on the fibre length, 20% of the fibres are in class 1 from 100 nm to 200 nm, with a class centre of 150 nm and 80% of the fibres are in class 2 from 200 nm to 400 nm with a class centre of 300 µm. Assuming 1 mm total length, the contribution of class 1 are 200 µm and the number of fibres is estimated as 200 µm / 150 nm to 1333, and the number of fibre ends is double, hence 2666. For class 2 the number of fibre ends is 5333. The total number of fibre ends in the dispersion is 8000.

The number of cumulative fibre ends for a hypothetical total length of 1 mm is reported as $N_{Ends,NF=1}$ in Table S6 and represent the expected number in case all cellulosic material is the dispersion is present as fibril which is not the case. The hypothetical number of fibre ends is calculated by weighting the dispersion with the nanofraction NF as $N_{Ends} = N_{Ends,NF=1}(\text{Case})\cdot NF+N_{Ends,NF=1}(\text{Pulp})\cdot(1-NF)$. n^*_{End} is the number of fibre ends normalized by the value for fibres, i.e., N_{Ends}(Pulp) and $n^*_{End,rate}$ is the relative increase with respect to the previous value, i.e., the slope of fibre ends increase.

Table S6: Estimated increase of the fibre ends from the fibril length distribution assuming a length of the elementary fibril in the CMCe fibre $l_{Fibril,CMCe}$ of 1100 nm.

Case	NF	$N_{Ends,NF=1}$	N_{Ends}	n^*_{End}	$n^*_{End,rate}$
Pulp, CMCe	0.00	1818	1818	1.00	-
UH100, 5 min	0.12	4933	2200	1.21	1.21
UH100, 15 min	0.69	5343	4253	2.34	1.93
UH100, 30 min	0.91	6812	6362	3.50	1.50

Three conclusions can be drawn from the results presented in Table S6. First, on hand of $N_{Ends,NF=1}$, that the number of fibre ends strongly increases by the fibrillation which shortens the fibrils when breaking the intra-fibre fibril-agglomerate. Second, on hand of n^*_{End}, that the number of fibre ends present in the dispersion initially increases slowly and in dependence on the increase of the nanocellulose fraction NF. Third on hand of $n^*_{End,rate}$ that the relative increase of the fibre ends decreases. In Figure 7d in the main document we document an increase of the short CNF fraction from UH100 15 min treatment to 30 min treatment. The increase in fibre ends number by CNF breakage is lower compared to the breakage of the fibrils during the initial fibrillation. n^*_{End} and $n^*_{End,rate}$ depend on the assumed length of the elementary fibril in the

CMCe fibre, taken as 1100 nm for the results shown in Table S6. In Table S7 we give $n^*_{End,rate}$ for different length of the elementary fibril in the CMCe fibre, $l_{Fibril,CMCe}$. For all cases, the relative increase of fibre ends $n^*_{End,rate}$ drops.

Table S7: Relative increase in the number of fibre ends for various assumed values of length of the elementary fibril in the CMCe fibre $l_{Fibril,CMCe}$.

$l_{Fibril,CMCe}$ [nm]	800	1100	1500	2000	3000	5000
	$n^*_{End,rate}$					
UH100, 5 min	1.12	1.21	1.33	1.48	1.78	2.39
UH100, 15 min	1.59	1.93	2.31	2.70	3.27	3.99
UH100, 30 min	1.44	1.50	1.54	1.57	1.60	1.63

4. Modelling of the Cellulose Fibre Fibrillation by Batch Sonication

Bath sonication is the focused treatment within the *active zone* below the sonotrode tip, as shown in Figure 2c of the main document, and mixing, respectively exchange of material between the *active zone* and the bulk which can be considered a *dead volume* (sketched in Figure S26a). The treatment and mixing take place simultaneous sly. The effect is a dispersion of properties what results, for example, to the observed quick increase in *NF* at the beginning, i.e., 5 min, and the presence of cellulose fibres after long exposure, i.e, 30 min. For a better understanding of the process, we model the cellulose fibre fibrillation into *NF*, accounting for one treatment in the *active zone* and perfect mixing per time-step. The model is sketched in Figure S26b. The active zone volume fraction, *tVol*, is measured to $1.29 \cdot 10^3$ and $0.6 \cdot 10^3$ for UH100 and UH40, respectively (see section 3.1 of the main document). The fibrillation efficiency per time-step, i.e., conversion, *con*, is fitted to match the results for CMCe UH100 and resulted to be 1.1 s^{-1}. *con* for UH40 is calculated assuming a scaling of the treatment intensity with the caloric power (Table X of the main document). That is reasonable, as every bubble implosion increases the temperature and has the chance of damaging the fibre. *con* for UH40 calculated to *con* (UH100)$\cdot P_{FS,0.4\%}$(UH40)/ $P_{FS,0.4\%}$(UH100) = 33 W/112 W = 0.32.

The explicit calculation scheme is presented in Figure S26b and the results are presented in Figure 11 of the main document. The modelled *NF* matches well the results of UH100 and UH40 what strengthens our assumption that sonication batch fibrillation includes a mixing problem for what the increase of *NF* with time decreases. Further, the conversion can be scaled between sonication settings, i.e., oscillation amplitude, knowing the *active* volume and the caloric power input.

With the established model we werde then able to probe the impact of the batch size, i.e., size of the glass beaker, and active zone size, i.e., shape of the ultrasonic horn tip, by varriing the active zone volume fraction *tVol*. In Figure S27a we show the normalized sonication time $t^* = t \cdot$con needed to reach a targeted fraction of nanocellulosic material *NF*. The sonication time t^* scales inversely with *tVol* until *tVol* ~ 0.1 (i.e., 1.3 mL bath volume for our case of UH100) and decays faster for higher values of *tVol*. Expectatly, it takes more time to treat a larger batch volume than a smaller batch volume. Fibrillation of fibres by sonication however co-exists with the continued shortening of the fibrils produced from the fibres [12]. Longer treatment times t^*

hence increase the chance for fibril shortening, especially of the fibrils produced early in the treatment. A smaller *tVol* and a lower t^* decreases the number of breakage events on the fibrils and is expected to result in a larger mean fibril length.

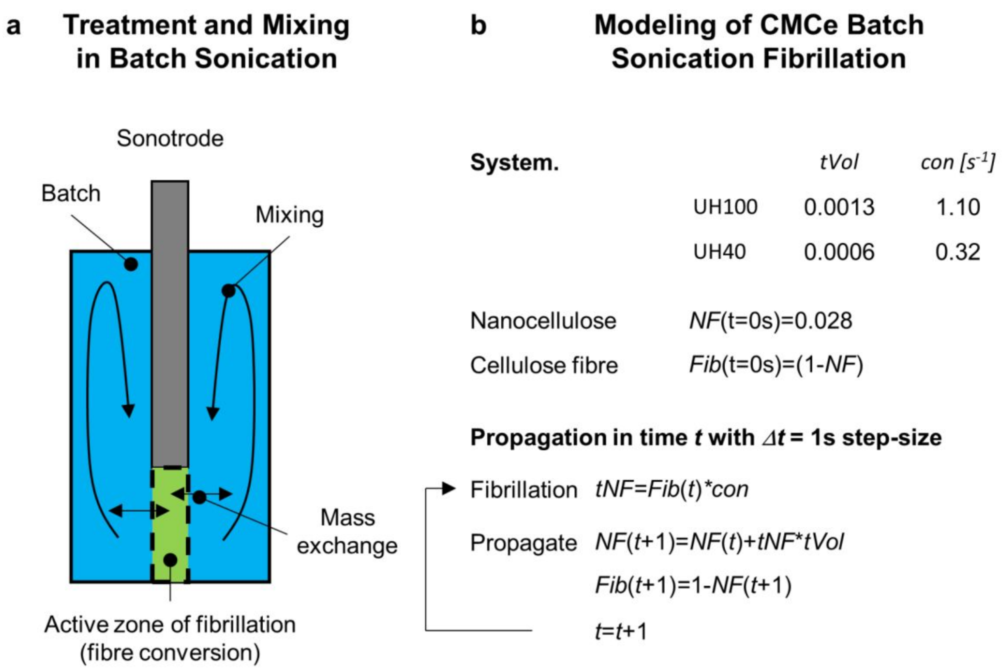

Figure S26: Modelling of the Batch Sonication Fibrillation. a: System definition. b: Explicit propagation of *NF* and the resulting fibre concentration *Fib*=(1-*NF*).

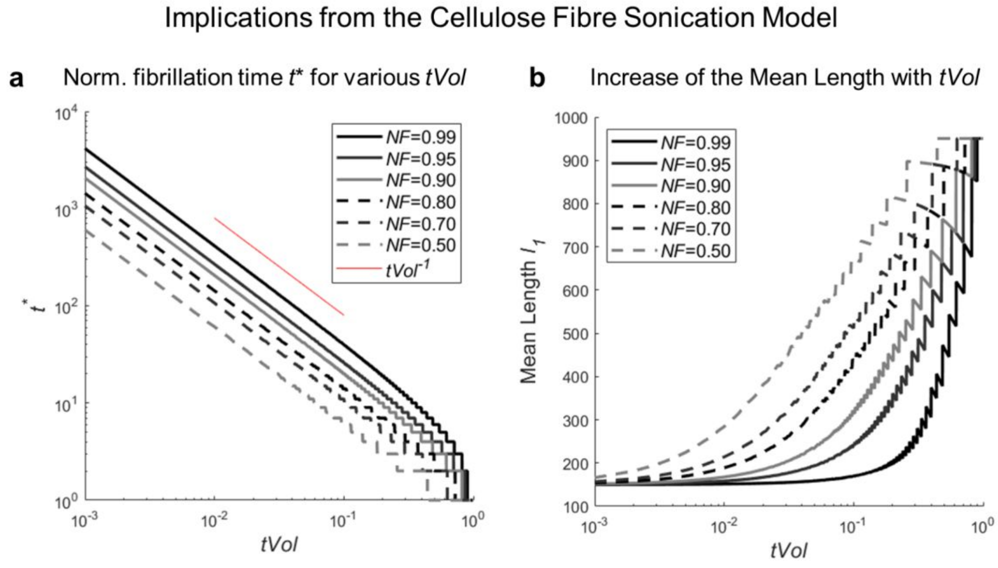

Figure S27: Implications from the cellulose fibre sonication model. a: the dependence of the sonication time on the active zone volume fraction *tVol*. The needed treatment time to reach a target conversion, i.e., *NF* value, scales inversely with *tVol* up to ca. 0.1 when the decay increase. b: Estimations of the cellulose nanofibril length development for various *tVol*.

We tested the hypothesis with a simple commination model, assuming a fibril breakage in half, until a length of 150 nm is reached whereafter no further breakage occurs (i.e., the limiting length is assumed as 150 nm to avoid an unrealistic comminution to atomistic scales). Fibrils at a length of 1000 nm are seeded for increasing *NF* at an increment of 10^{-4}. I.e., for every increase of *NF* of 10^{-4}, one fibril with a length of 1000 nm is added to the fibril population. An arbitrary and low value chosen to allow a quick computation. The dispersion length-weighted mean fibre length l_l is shown in Figure S27b. Indeed, l_l decreases with a decreasing *tVol*, what is most severe if a complete fibrillation, i.e., *NF* = 0.99, is aimed for. The implications are, that short cellulose nanofibrils (CNF) can be produced in a large batch with long sonication. To produce long CNF, small batch sizes (probably in the range of mL for the common laboratory sonotrodes) need to be used. It may hence be more practical to move from batch to continued sonication treatment for the one-pot production of long CNF from cellulose fibres.

5. ADDITIONAL IMAGES OF THE FIBRE AND FIBRILS FROM TREATMENT

5.1. Light Optical Microscopy (LOM) Images

5.1.1. Dissolving cellulose fibre pulp (DCe)

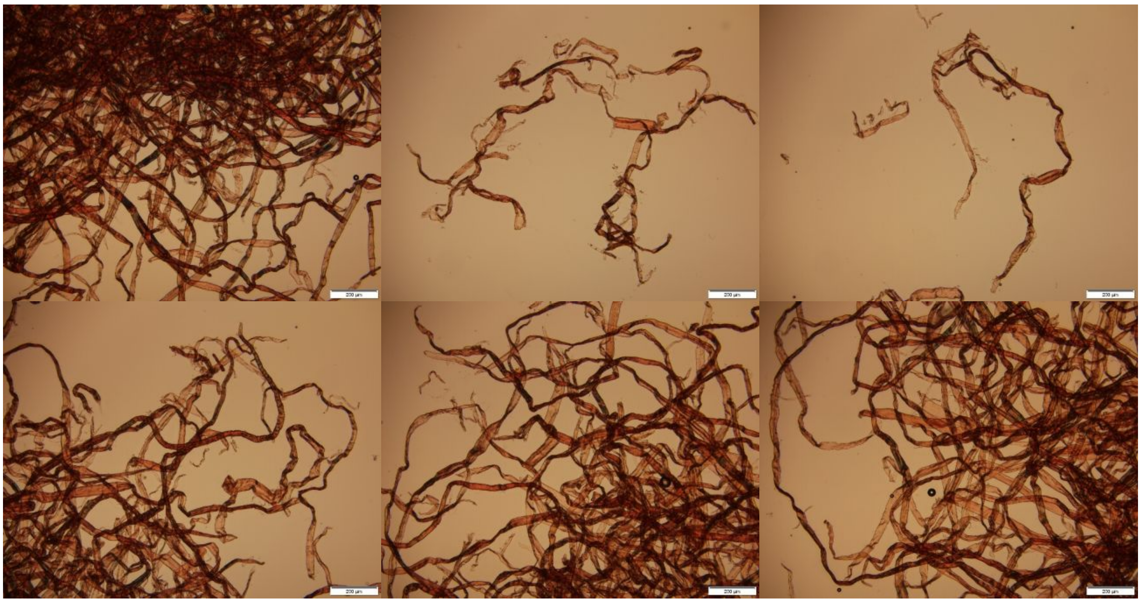

Figure S28: DCe pulp fibre.

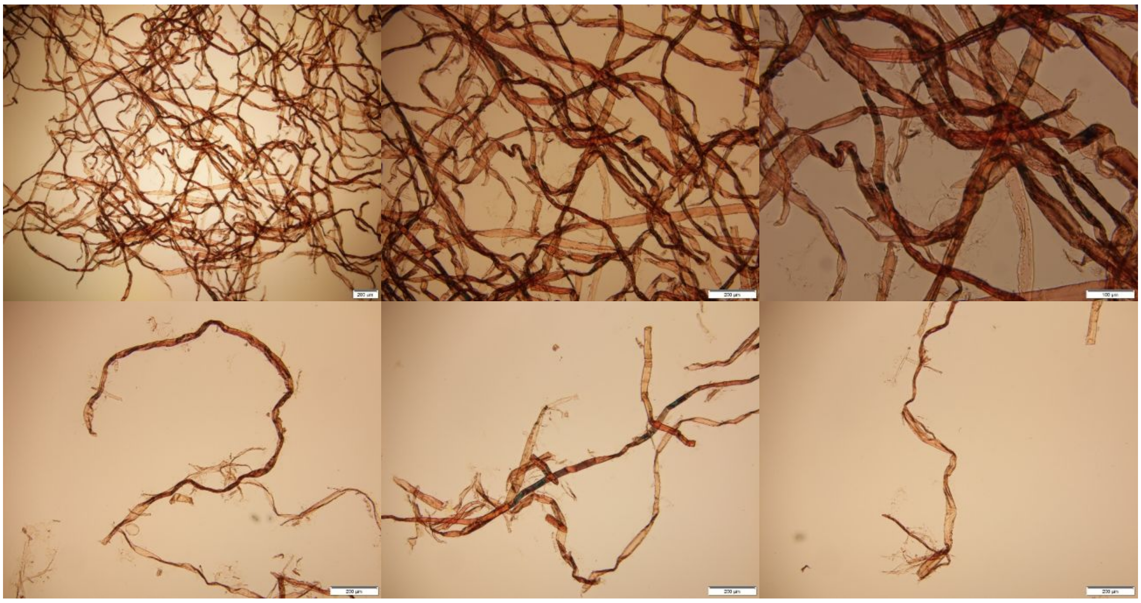

Figure S29: LOM images of DCe after BT 5 bar treatment for 60 min.

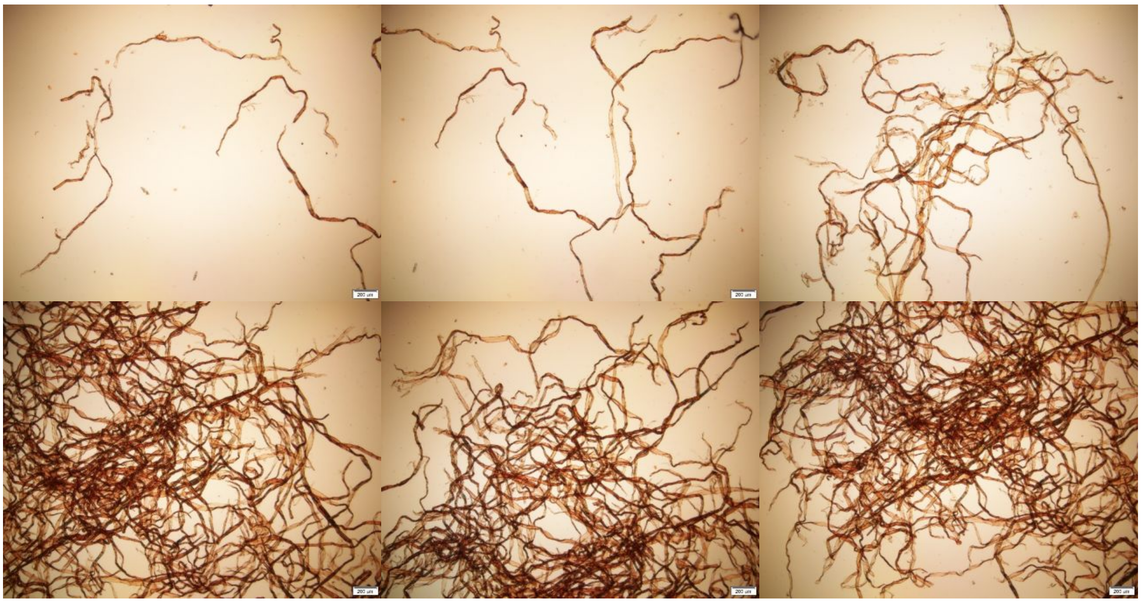

Figure S30: LOM images of DCe after UH40 treatment for 12 min and 30 s.

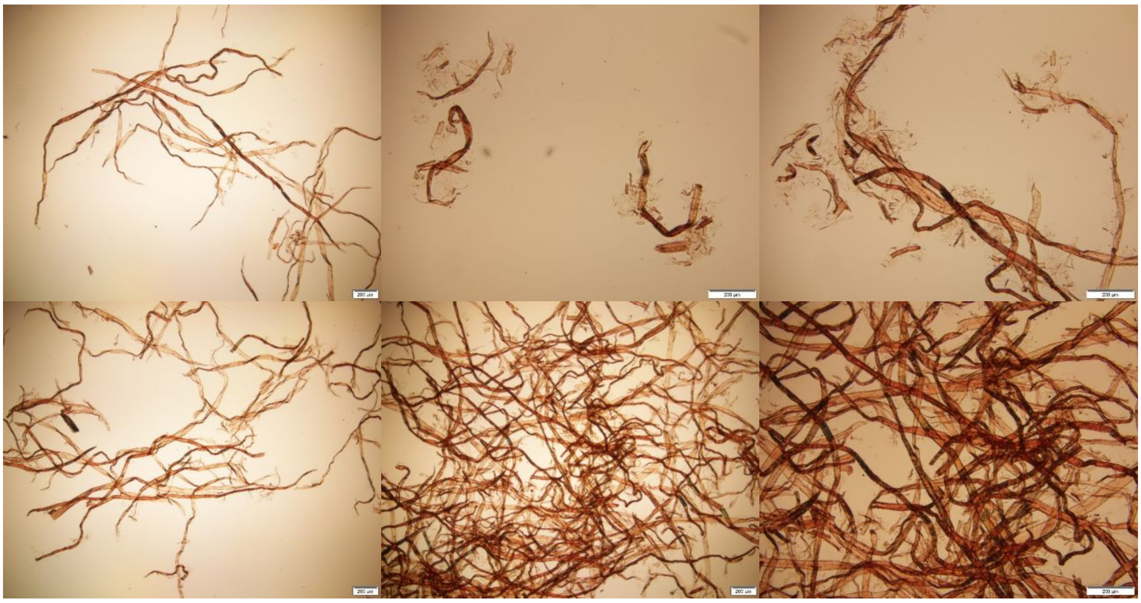

Figure S31: LOM images of DCe after UH100 treatment for 5 min.

5.1.2. Enzymatic Pre-Treated Cellulose (ECe)

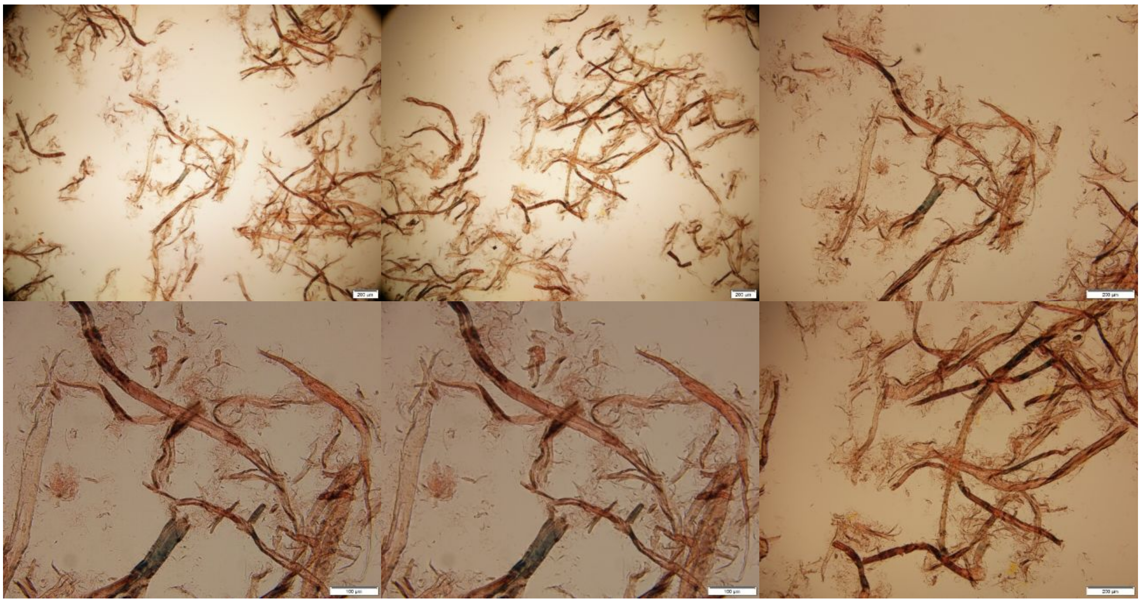

Figure S32: LOM images of ECe pulp fibre.

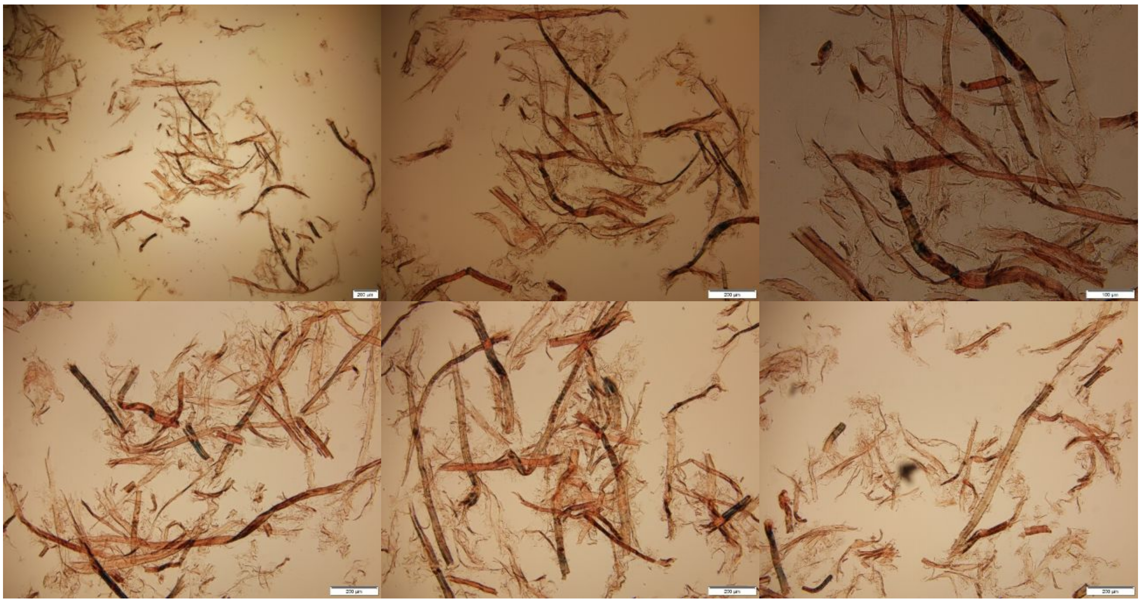

Figure S33: LOM images of ECe after BT 5 bar treatment for 60 min.

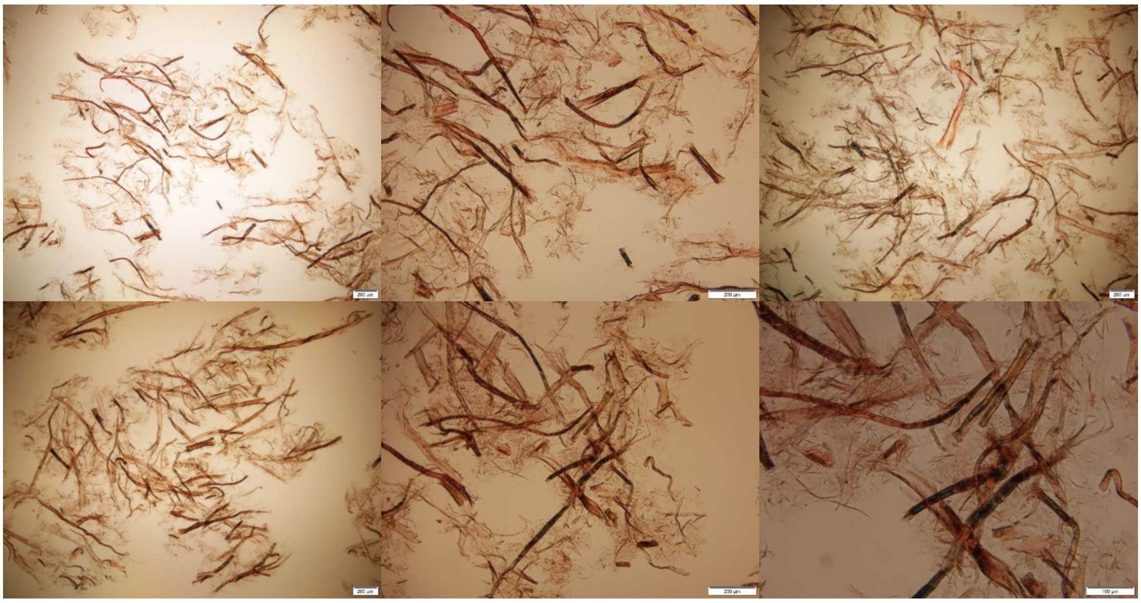

Figure S34: LOM images of ECe after UH40 treatment for 12 min and 30 s.

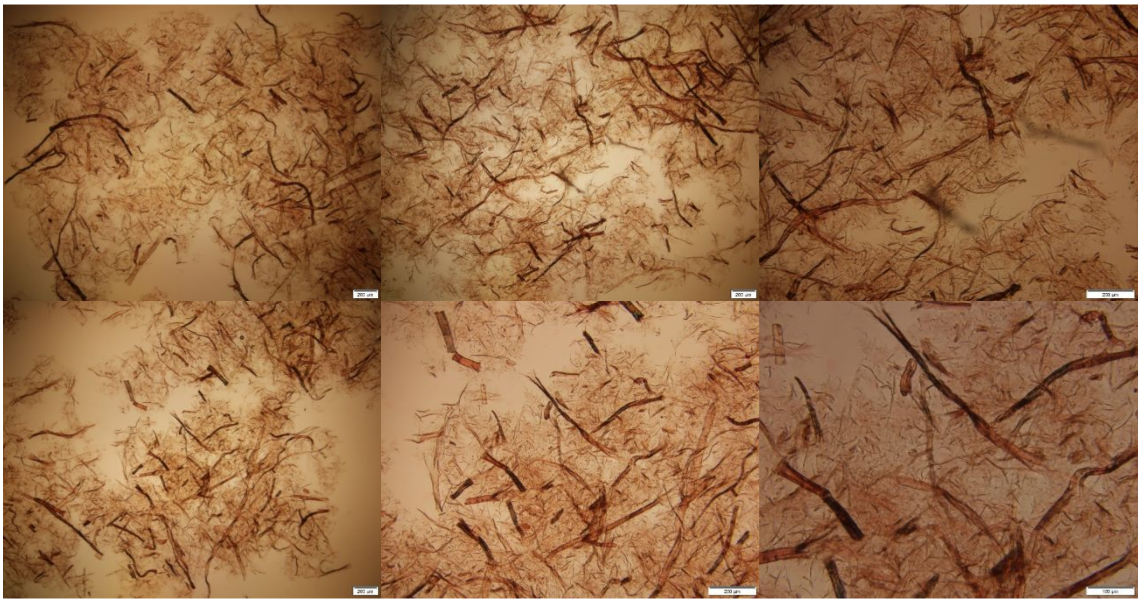

Figure S35: LOM images of ECe after UH100 treatment for 5 min.

5.1.3. Carboxymethylated Cellulose (CMCe)

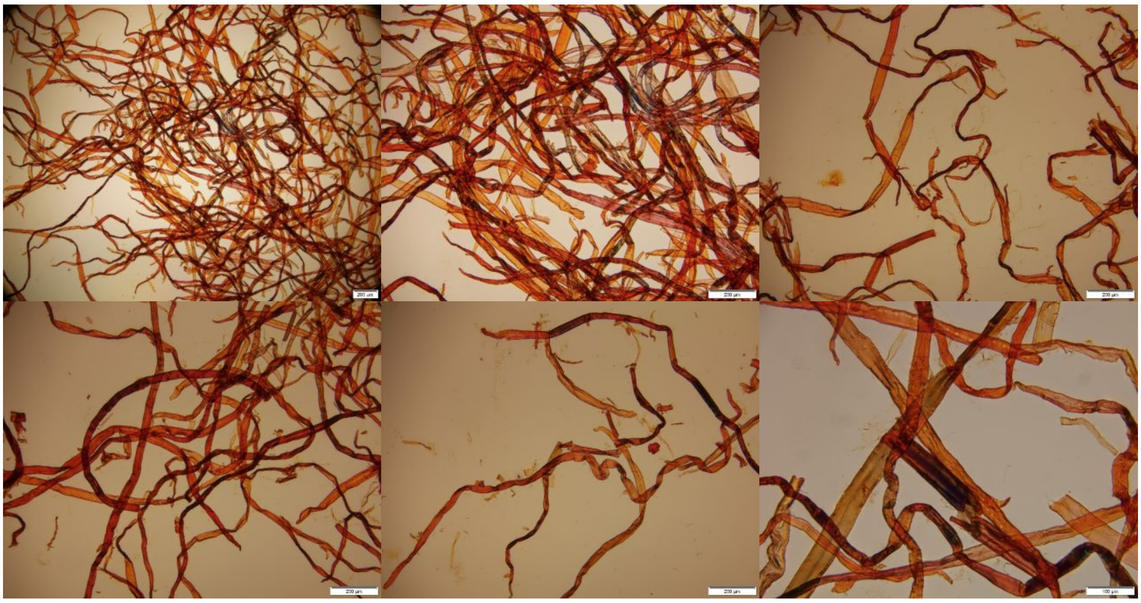

Figure S36: LOM images of CMCe pulp fibre.

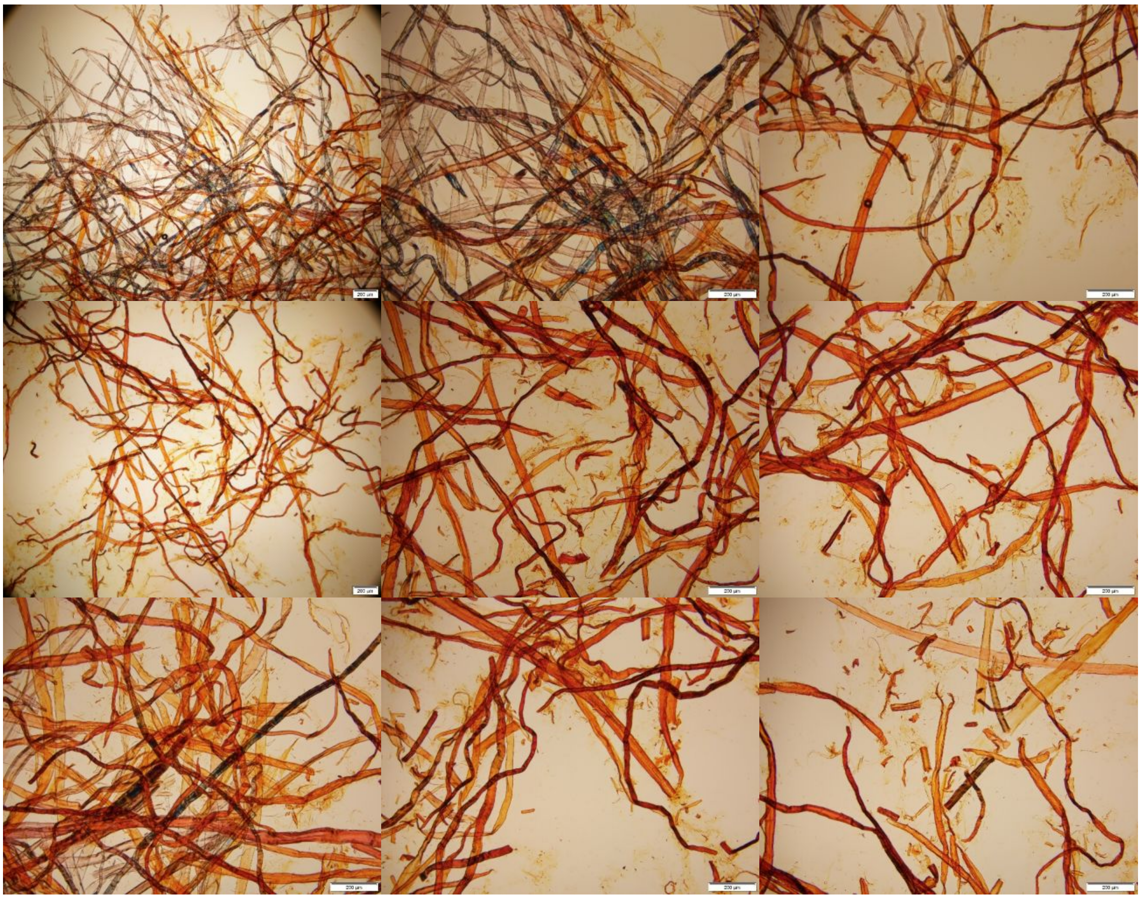

Figure S37: LOM images of CMCe after BT 5 bar treatment for 60 min.

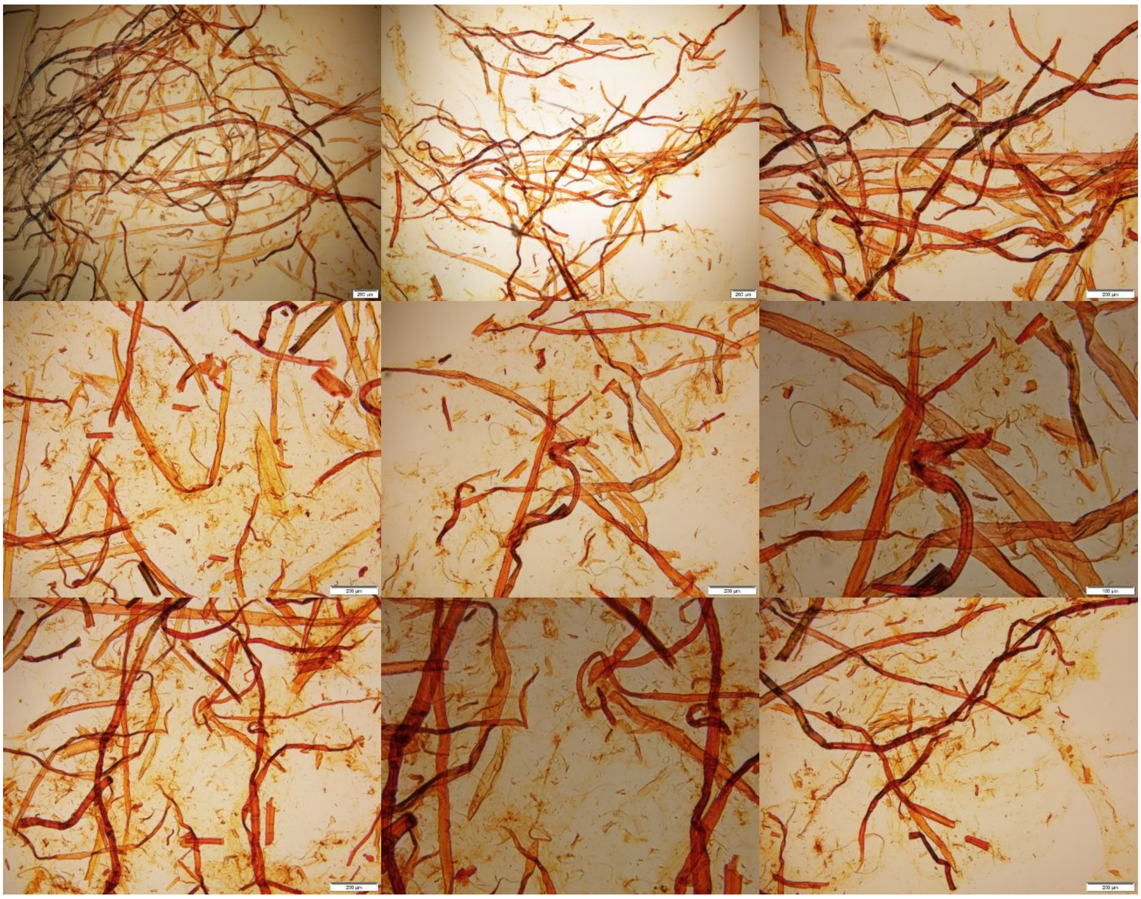

Figure S38: LOM images of CMCe after BT 7 bar treatment for 60 min.

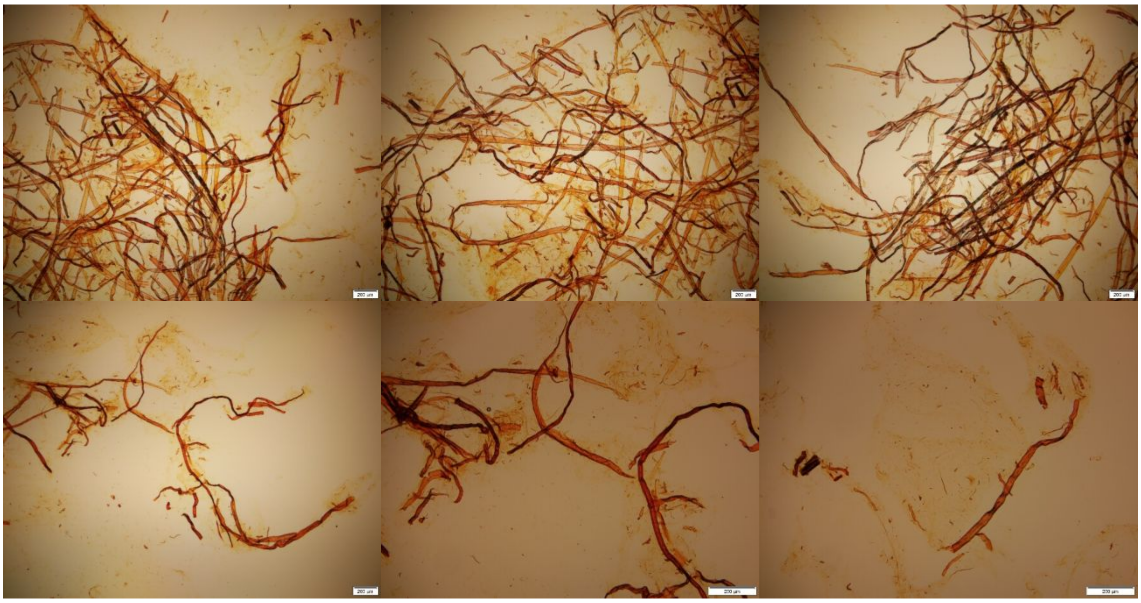

Figure S39: LOM images of CMCe at 2% after BT 5 bar treatment for 60 min.

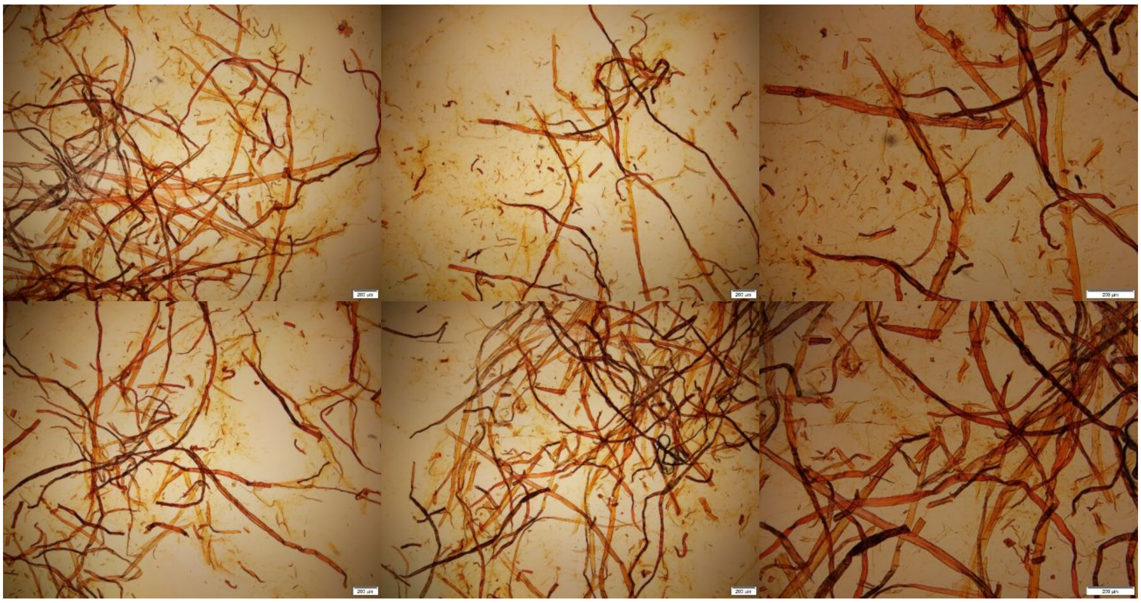

Figure S40: LOM images of CMCe at 2% after BT 5 bar treatment for 105 min.

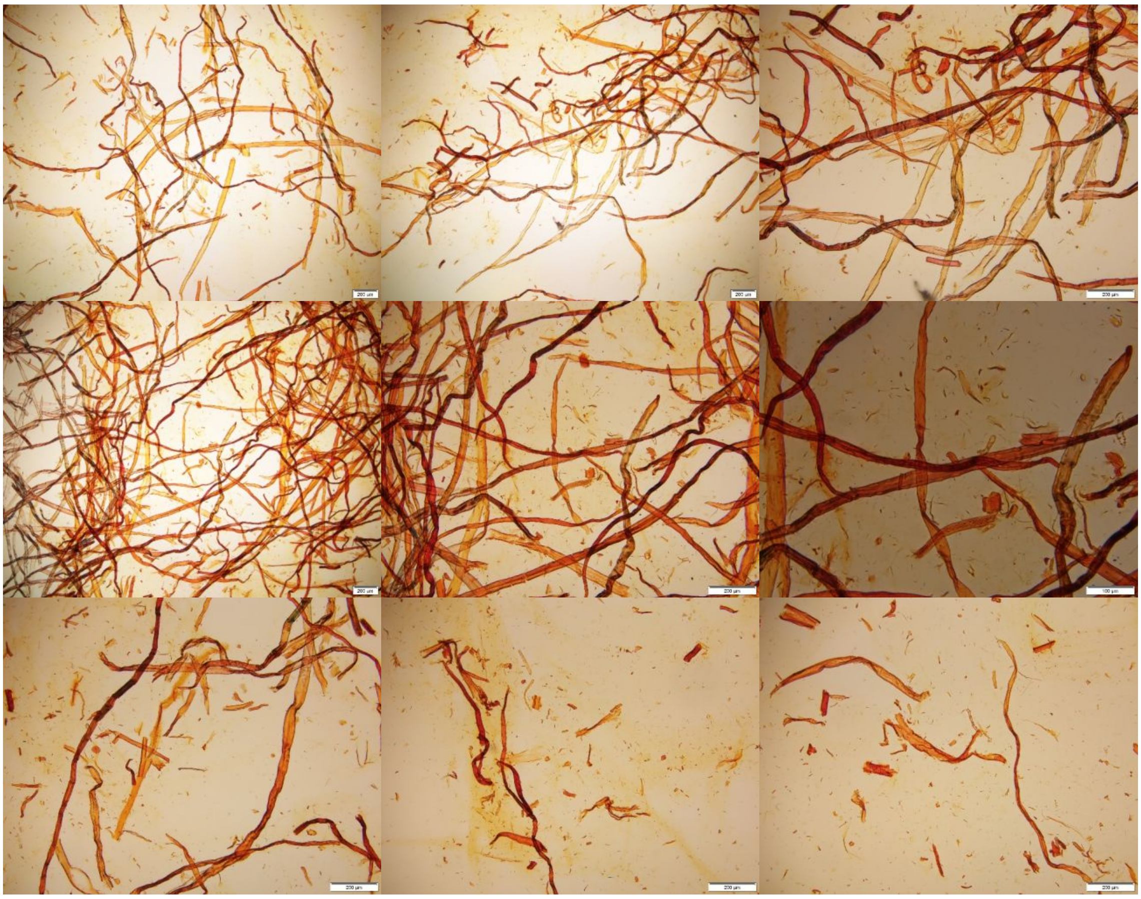

Figure S41: LOM images of CMCe after UH40 treatment for 12 min and 30 s.

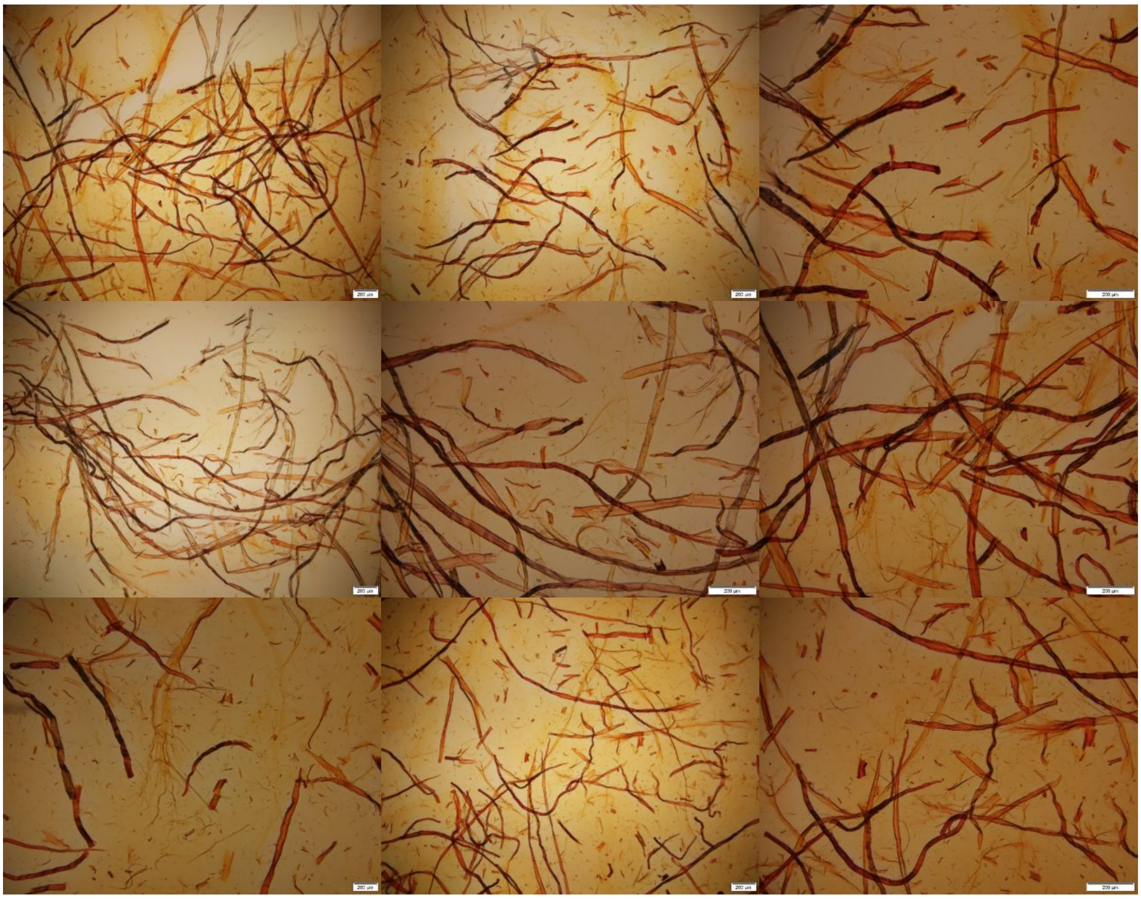

Figure S42: LOM images of CMCe after UH100 treatment for 5 min.

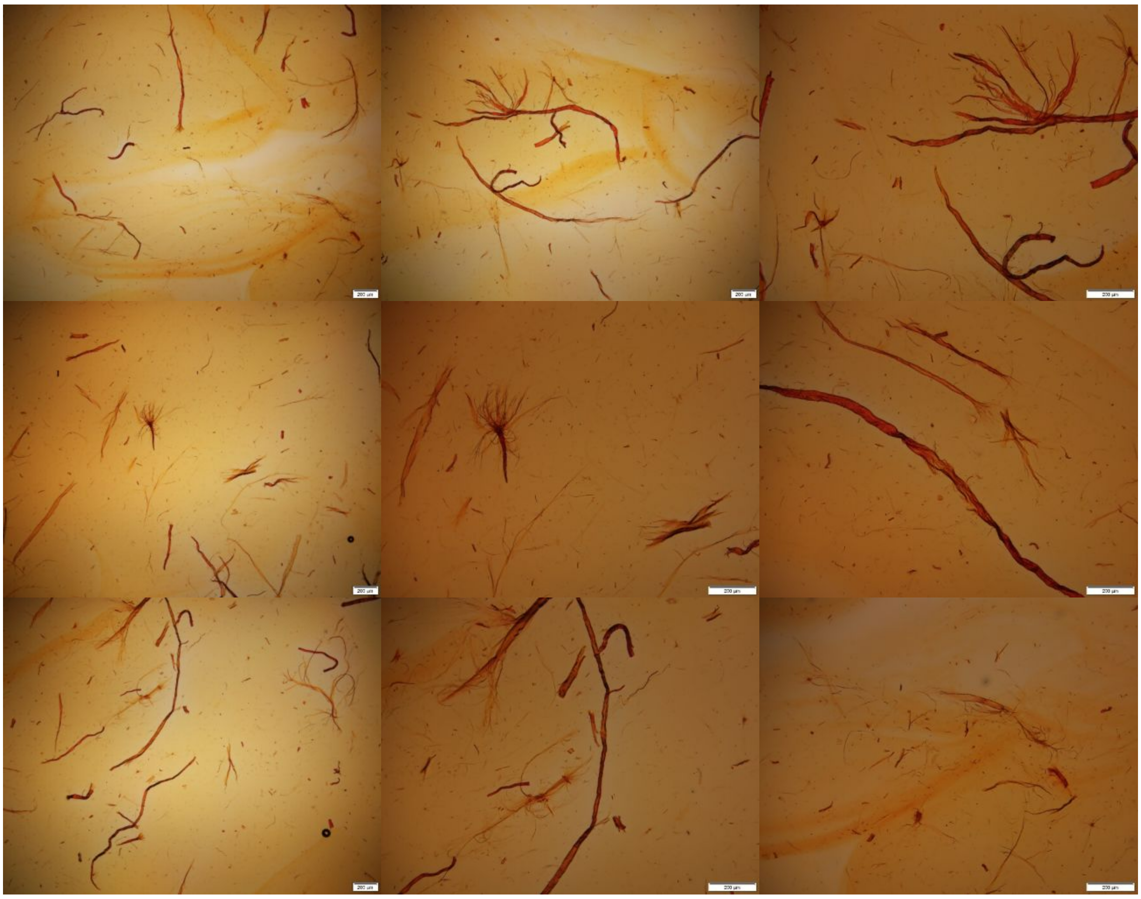

Figure S43: LOM images of CMCe after UH100 treatment for 15 min.

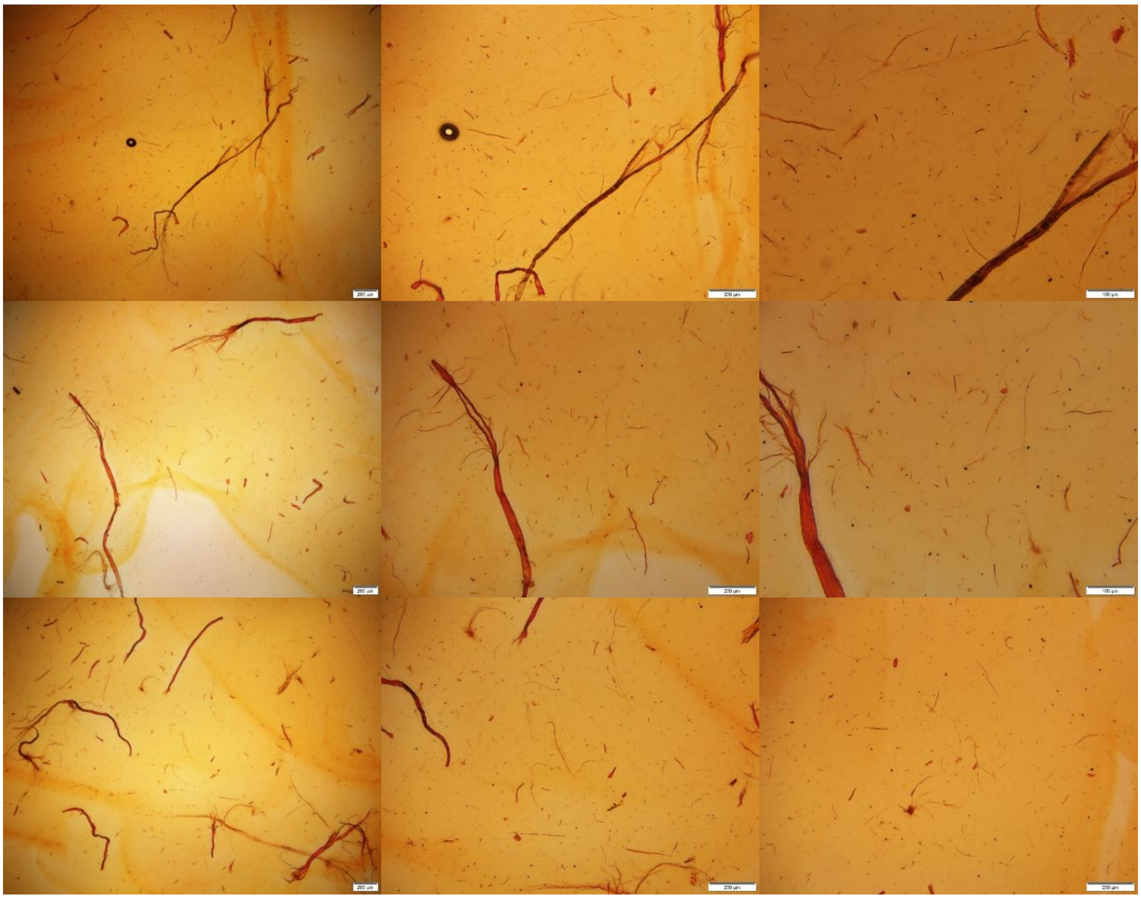

Figure S44: LOM images of CMCe after UH100 treatment for 30 min.

5.1.4. TEMPO oxidized cellulose (TCe)

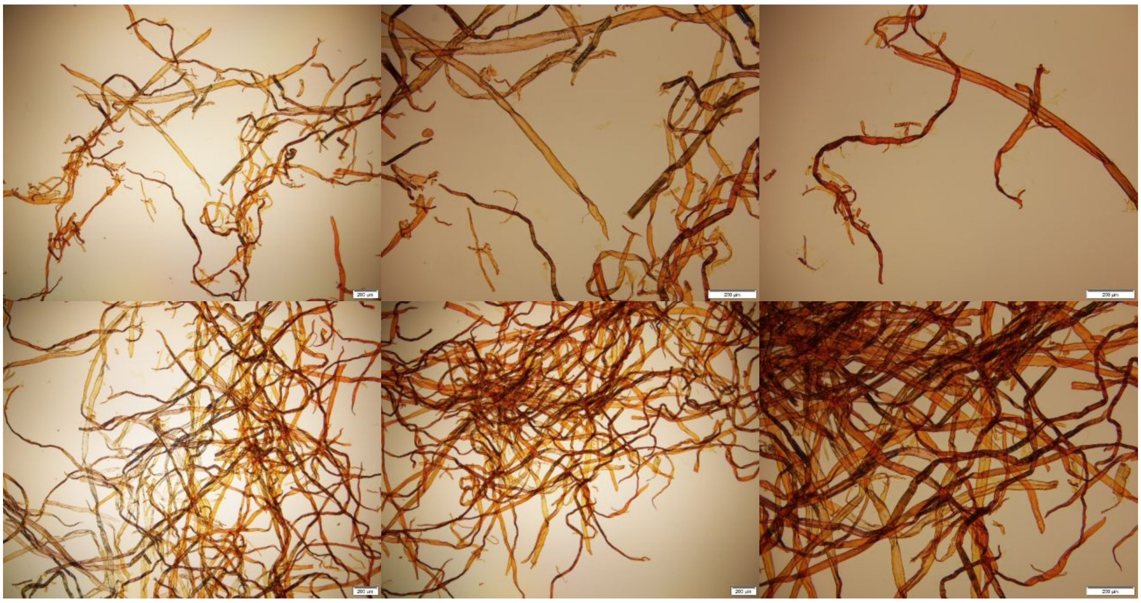

Figure S45:LOM images of TCe pulp fibre.

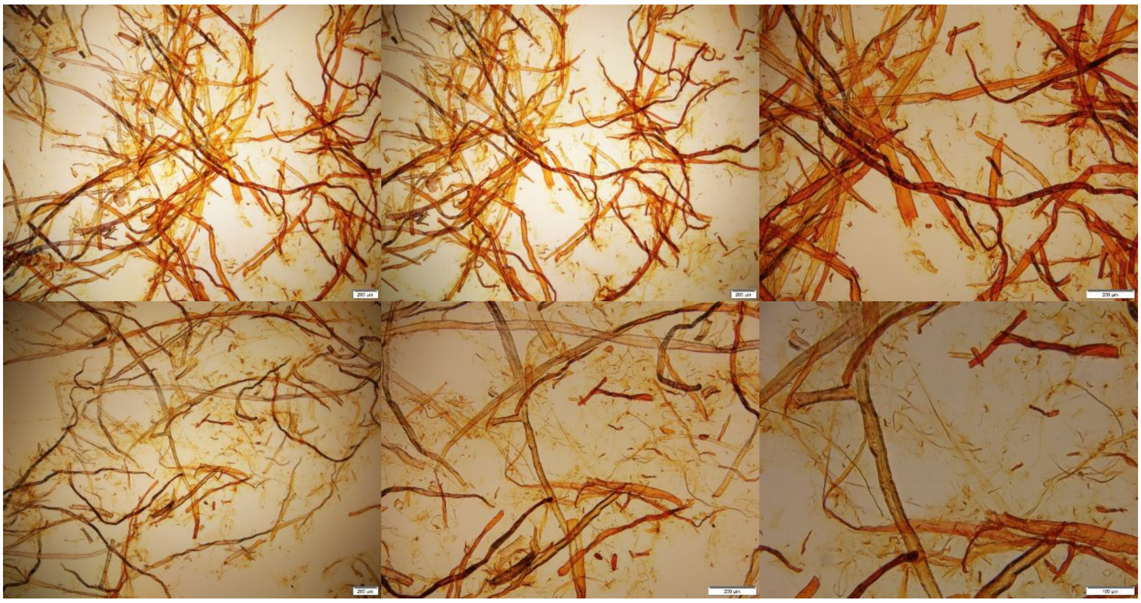

Figure S 46: LOM images of TCe after BT 5 bar treatment for 60 min.

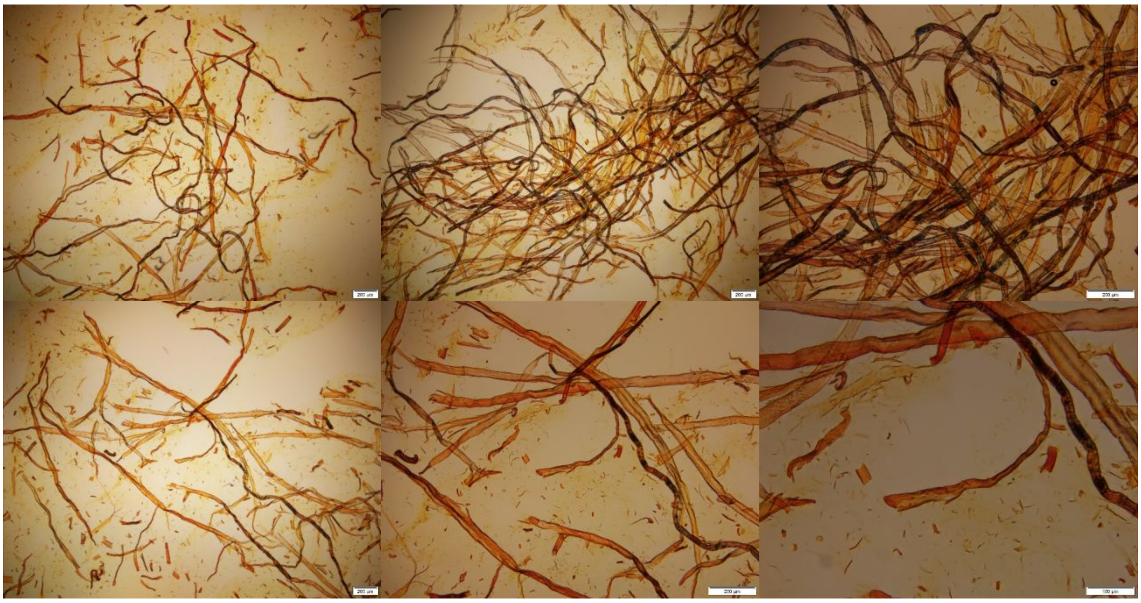

Figure S 47: LOM images of TCe after UH40 treatment for 12 min and 30 s.

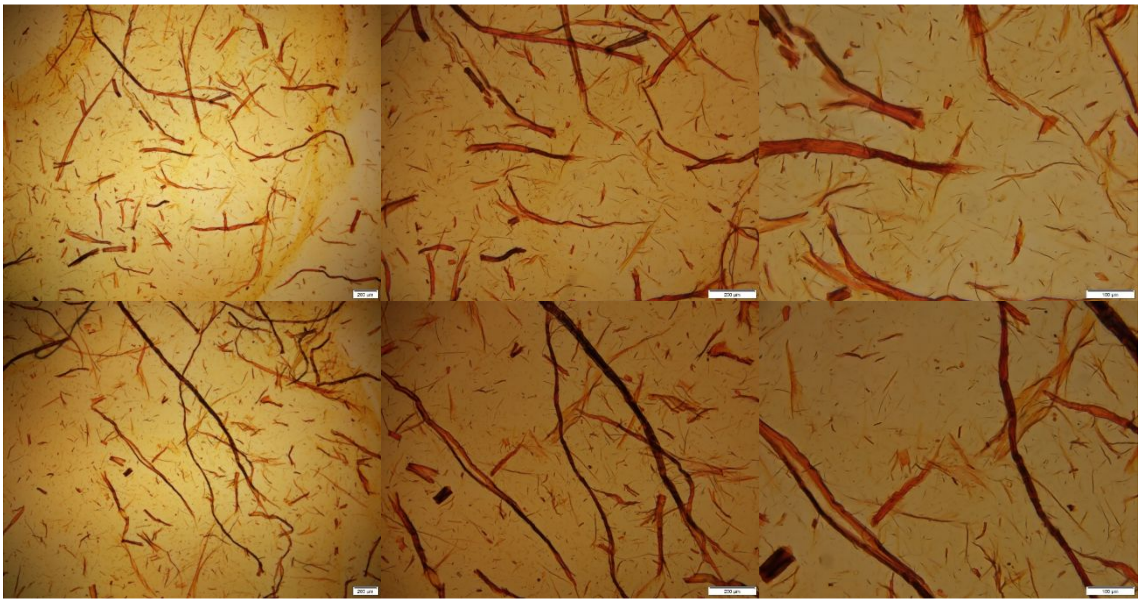

Figure S 48: LOM images of TCe after UH100 treatment for 5 min.

5.2. Scanning Electron Microscopy (SEM) Images

5.2.1. Carboxymethylated Cellulose (CMCe)

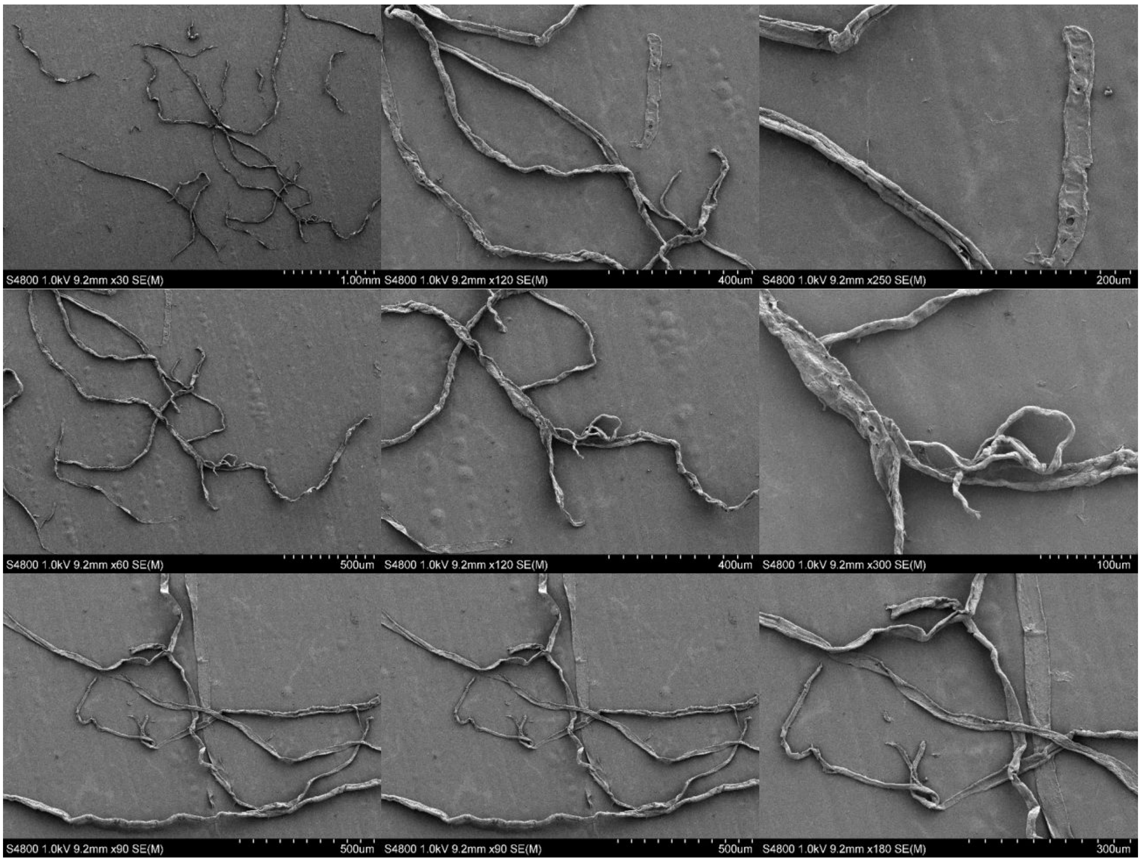

Figure S49: SEM images of CMCe pulp fibre.

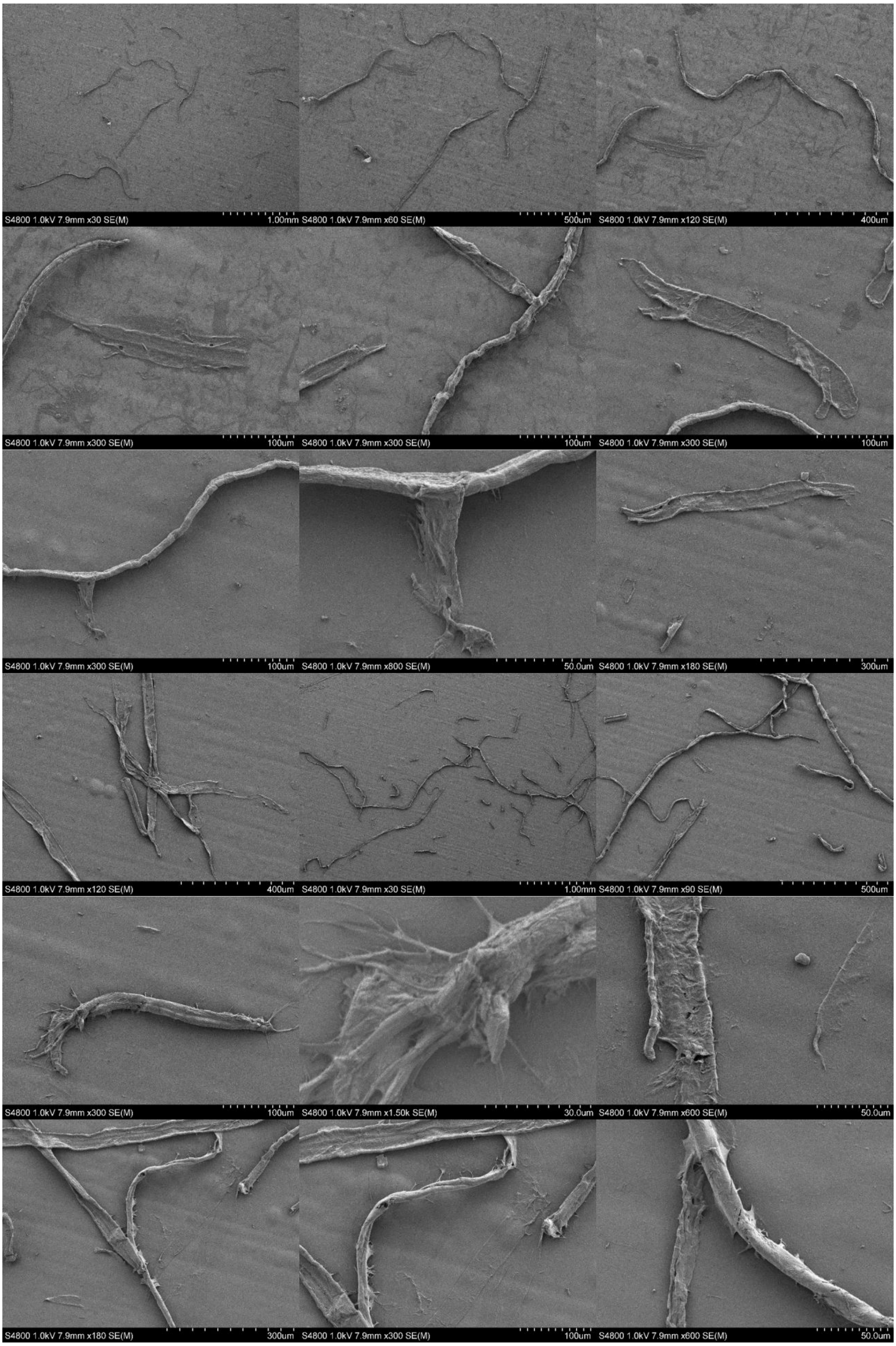

Figure S 50: SEM images of CMCe after BT 5 bar treatment for 60 min.

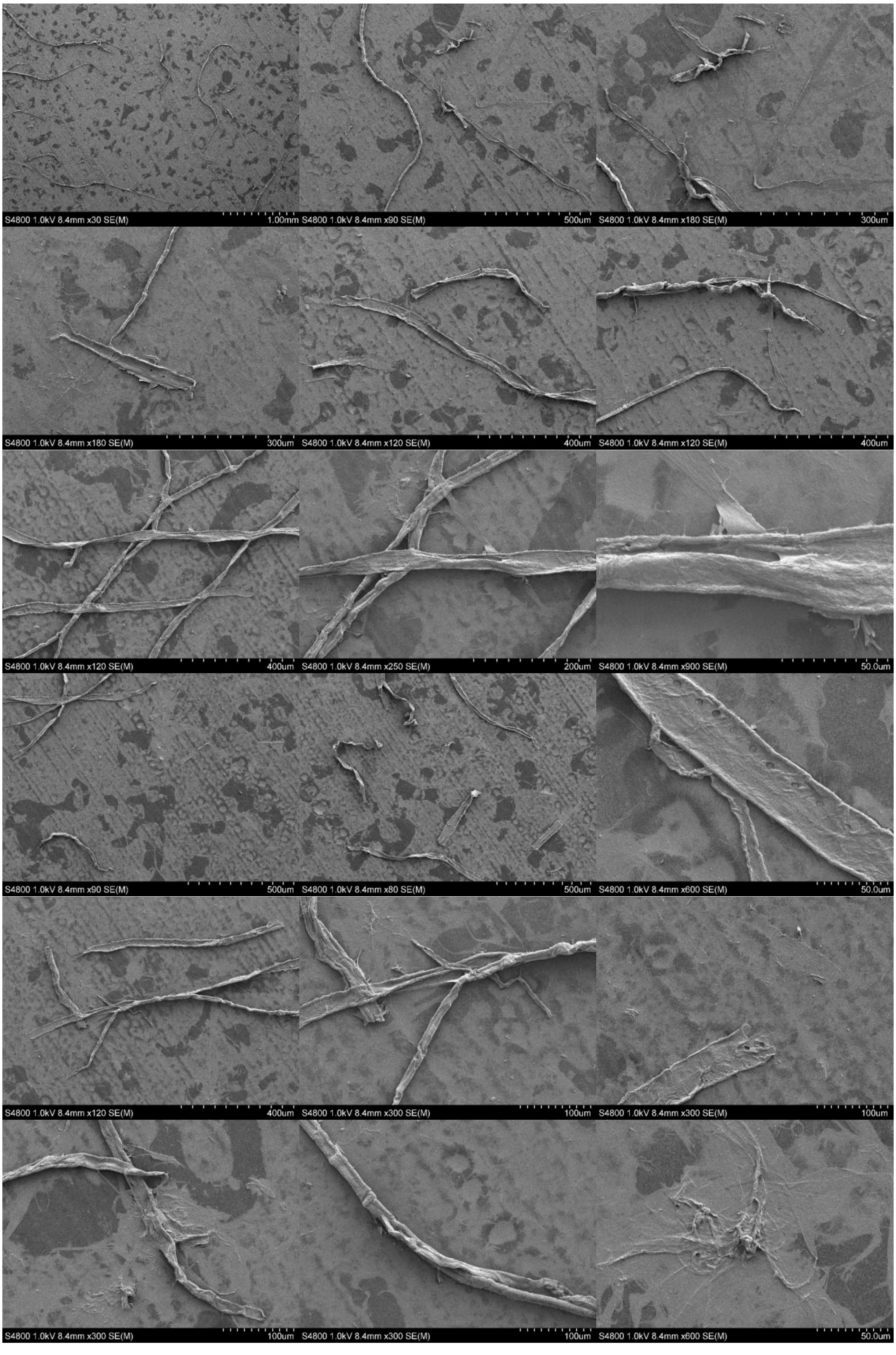

Figure S51: SEM images of CMCe after BT 7 bar treatment for 60 min.

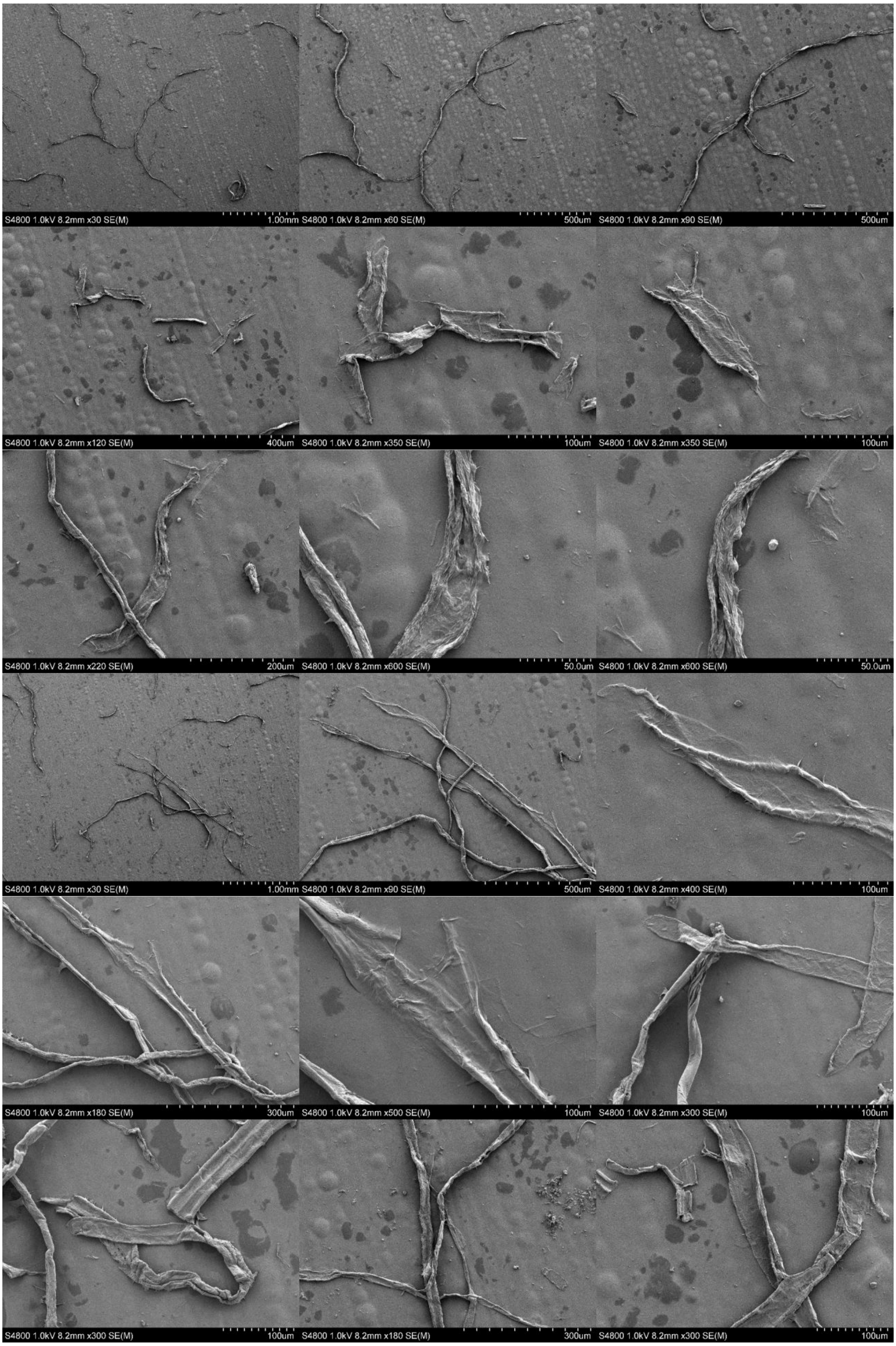

Figure S 52: SEM images of CMCe after UH40 treatment for 12 min and 30 s.

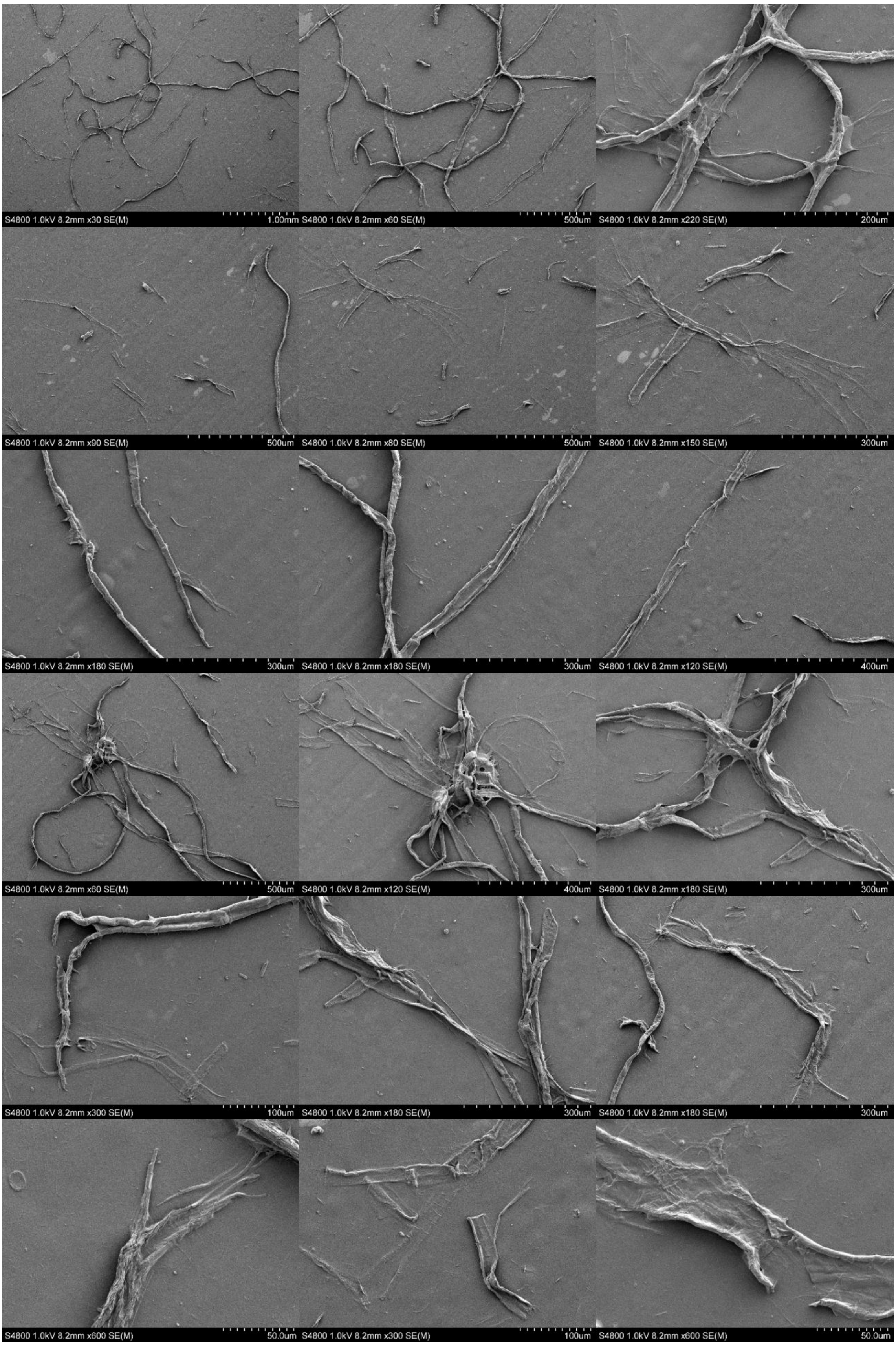

Figure S53: SEM images of CMCe after UH100 treatment for 5 min.

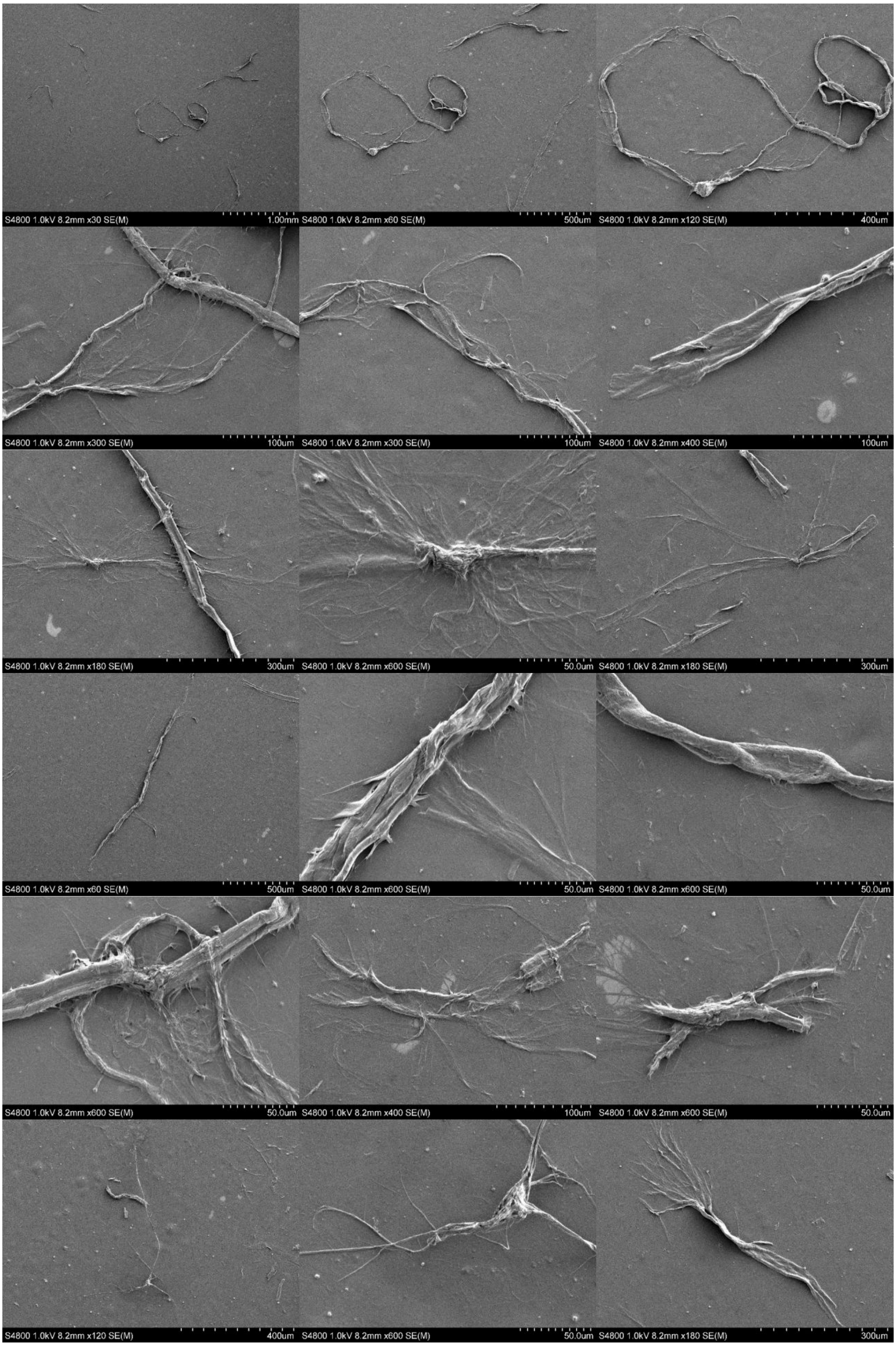

Figure S54: SEM images of CMCe after UH100 treatment for 15 min.

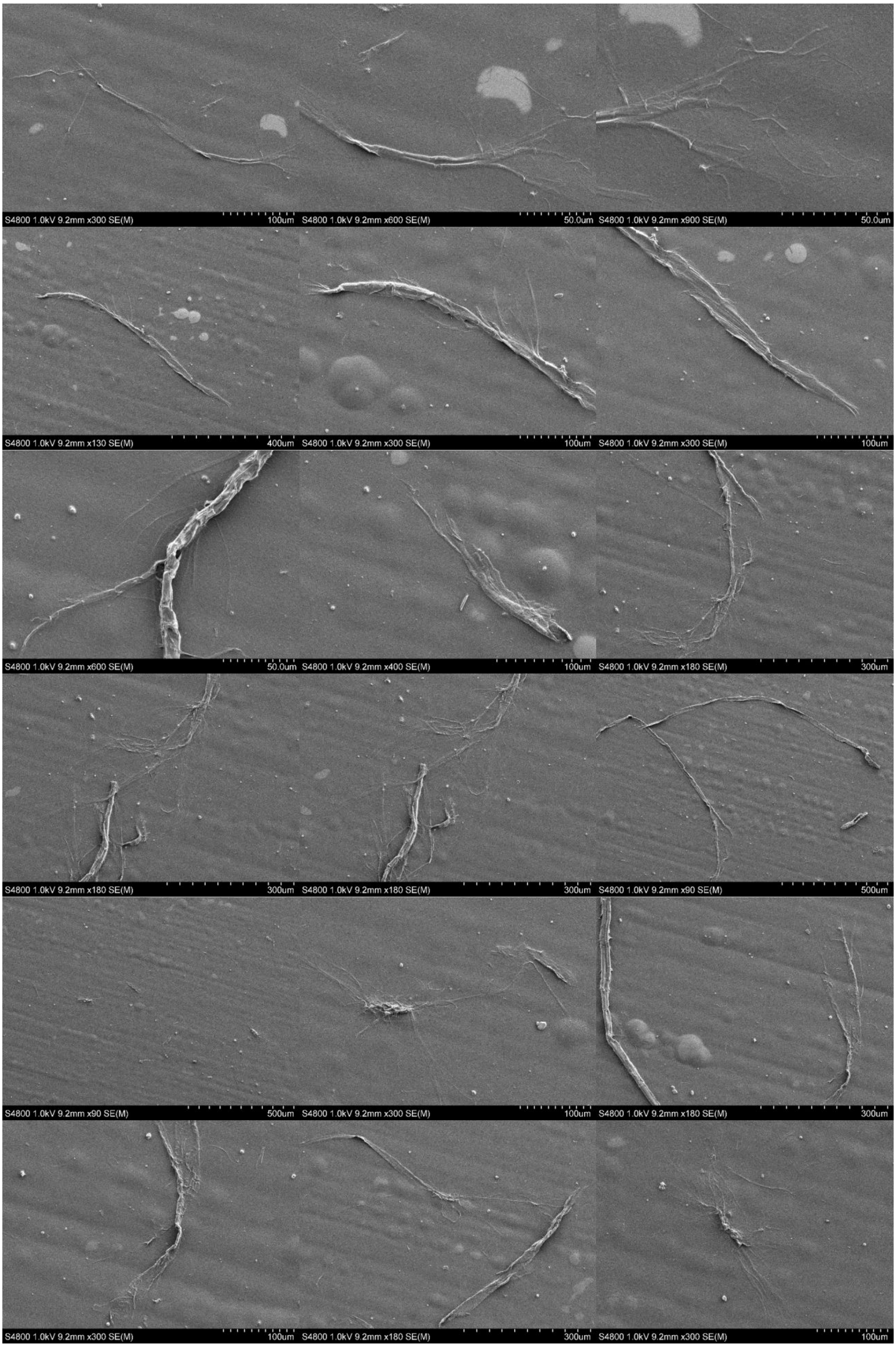

Figure S55: SEM images of CMCe after UH100 treatment for 30 min.

6. References in the Supplement Material

(1) Redlinger-Pohn, J. D.; Brouzet, C.; Aulin, C.; Engström, Å.; Riazanova, A. V.; Holmqvist, C.; Lundell, F.; Söderberg, L. D. Mechanisms of Cellulose Fiber Comminution to Nanocellulose by Hyper Inertia Flows. *ACS Sustain. Chem. Eng.* **2022**, *Accepted f*, acssuschemeng.1c03474. https://doi.org/10.1021/acssuschemeng.1c03474.

(2) Fan, F. G.; Ahmadi, G. A Sublayer Model for Wall Deposition of Ellipsoidal Particles in Turbulent Streams. *J. Aerosol Sci.* **1995**, *26* (5), 813–840. https://doi.org/10.1016/0021-8502(95)00021-4.

(3) Segal, L.; Creely, J. J.; Martin, A. E.; Conrad, C. M. An Empirical Method for Estimating the Degree of Crystallinity of Native Cellulose Using the X-Ray Diffractometer. *Text. Res. J.* **1959**, *29* (10), 786–794. https://doi.org/10.1177/004051755902901003.

(4) French, A. D.; Santiago Cintrón, M. Cellulose Polymorphy, Crystallite Size, and the Segal Crystallinity Index. *Cellulose* **2013**, *20* (1), 583–588. https://doi.org/10.1007/s10570-012-9833-y.

(5) Nam, S.; French, A. D.; Condon, B. D.; Concha, M. Segal Crystallinity Index Revisited by the Simulation of X-Ray Diffraction Patterns of Cotton Cellulose Iβ and Cellulose II. *Carbohydr. Polym.* **2016**, *135*, 1–9. https://doi.org/10.1016/j.carbpol.2015.08.035.

(6) French, A. D. Increment in Evolution of Cellulose Crystallinity Analysis. *Cellulose* **2020**, *27* (10), 5445–5448. https://doi.org/10.1007/s10570-020-03172-z.

(7) Park, S.; Baker, J. O.; Himmel, M. E.; Parilla, P. A.; Johnson, D. K. Cellulose Crystallinity Index: Measurement Techniques and Their Impact on Interpreting Cellulase Performance. *Biotechnol. Biofuels* **2010**, *3* (1), 10. https://doi.org/10.1186/1754-6834-3-10.

(8) del Cerro, D. R.; Koso, T. V.; Kakko, T.; King, A. W. T.; Kilpeläinen, I. Crystallinity Reduction and Enhancement in the Chemical Reactivity of Cellulose by Non-Dissolving Pre-Treatment with Tetrabutylphosphonium Acetate. *Cellulose* **2020**, *27* (10), 5545–5562. https://doi.org/10.1007/s10570-020-03044-6.

(9) Yao, W.; Weng, Y.; Catchmark, J. M. Improved Cellulose X-Ray Diffraction Analysis

Using Fourier Series Modeling. *Cellulose* **2020**, *27* (10), 5563–5579. https://doi.org/10.1007/s10570-020-03177-8.

(10) Wojdyr, M. Fityk : A General-Purpose Peak Fitting Program. *J. Appl. Crystallogr.* **2010**, *43* (5), 1126–1128. https://doi.org/10.1107/S0021889810030499.

(11) Daicho, K.; Saito, T.; Fujisawa, S.; Isogai, A. The Crystallinity of Nanocellulose: Dispersion-Induced Disordering of the Grain Boundary in Biologically Structured Cellulose. *ACS Appl. Nano Mater.* **2018**, *1* (10), 5774–5785. https://doi.org/10.1021/acsanm.8b01438.

(12) Zhou, Y.; Saito, T.; Bergström, L.; Isogai, A. Acid-Free Preparation of Cellulose Nanocrystals by TEMPO Oxidation and Subsequent Cavitation. *Biomacromolecules* **2018**, *19* (2), 633–639. https://doi.org/10.1021/acs.biomac.7b01730.

(13) Abidi, N.; Cabrales, L.; Haigler, C. H. Changes in the Cell Wall and Cellulose Content of Developing Cotton Fibers Investigated by FTIR Spectroscopy. *Carbohydr. Polym.* **2014**, *100*, 9–16. https://doi.org/10.1016/j.carbpol.2013.01.074.

(14) Oh, S. Y.; Yoo, D. Il; Shin, Y.; Seo, G. FTIR Analysis of Cellulose Treated with Sodium Hydroxide and Carbon Dioxide. *Carbohydr. Res.* **2005**, *340* (3), 417–428. https://doi.org/10.1016/j.carres.2004.11.027.

(15) Lichtenstein, K.; Lavoine, N. Toward a Deeper Understanding of the Thermal Degradation Mechanism of Nanocellulose. *Polym. Degrad. Stab.* **2017**, *146*, 53–60. https://doi.org/10.1016/j.polymdegradstab.2017.09.018.

(16) Lavoine, N.; Bras, J.; Saito, T.; Isogai, A. Improvement of the Thermal Stability of TEMPO-Oxidized Cellulose Nanofibrils by Heat-Induced Conversion of Ionic Bonds to Amide Bonds. *Macromol. Rapid Commun.* **2016**, *37* (13), 1033–1039. https://doi.org/10.1002/marc.201600186.

(17) Fujisawa, S.; Okita, Y.; Fukuzumi, H.; Saito, T.; Isogai, A. Preparation and Characterization of TEMPO-Oxidized Cellulose Nanofibril Films with Free Carboxyl Groups. *Carbohydr. Polym.* **2011**, *84* (1), 579–583. https://doi.org/10.1016/j.carbpol.2010.12.029.

(18) Maréchal, Y.; Chanzy, H. The Hydrogen Bond Network in I β Cellulose as Observed by Infrared Spectrometry. *J. Mol. Struct.* **2000**, *523* (1–3), 183–196. https://doi.org/10.1016/S0022-2860(99)00389-0.

(19) S. Dassanayake, R.; Acharya, S.; Abidi, N. Biopolymer-Based Materials from Polysaccharides: Properties, Processing, Characterization and Sorption Applications. In *Advanced Sorption Process Applications*; IntechOpen, 2019. https://doi.org/10.5772/intechopen.80898.

(20) Rosén, T.; He, H. R.; Wang, R.; Zhan, C.; Chodankar, S.; Fall, A.; Aulin, C.; Larsson, P. T.; Lindström, T.; Hsiao, B. S. Cross-Sections of Nanocellulose from Wood Analyzed by Quantized Polydispersity of Elementary Microfibrils. *ACS Nano* **2020**, *14* (12), 16743–16754. https://doi.org/10.1021/acsnano.0c04570.

(21) Zhou, Y.; Ono, Y.; Takeuchi, M.; Isogai, A. Changes to the Contour Length, Molecular Chain Length, and Solid-State Structures of Nanocellulose Resulting from Sonication in Water. *Biomacromolecules* **2020**, *21* (6), 2346–2355. https://doi.org/10.1021/acs.biomac.0c00281.
